# Supplementary material for: Impact of digital finance on enterprise green innovation: From the perspective of information asymmetry, consumer demand and factor market distortions
Source: PLoS One. 2023 Dec 14;18(12):e0295809. doi: 10.1371/journal.pone.0295809 (PMC10721172; doi:10.1371/journal.pone.0295809)
Supplement: S1 Table — (DOCX) [file pone.0295809.s003.docx]

**Table 2 Descriptive statistics.**

| Variable | N | Mean | SD | Min | p50 | Max | Skewness | Kurtosis |
| --- | --- | --- | --- | --- | --- | --- | --- | --- |
| PAT | 22781 | 8.318 | 48.44 | 0 | 0 | 1612 | 18.31 | 441.4 |
| INPAT | 22781 | 4.876 | 33.53 | 0 | 0 | 1381 | 21.43 | 605.1 |
| UPAT | 22781 | 3.442 | 19.02 | 0 | 0 | 709 | 19.09 | 488.9 |
| DIF | 22781 | 213.1 | 77.12 | 21.26 | 222.2 | 359.7 | -0.313 | 2.189 |
| DIFB | 22781 | 213.2 | 76.08 | -10.49 | 219.8 | 371.8 | -0.212 | 2.343 |
| DIFD | 22781 | 208.5 | 78.32 | 12.49 | 216.1 | 354.3 | -0.220 | 1.994 |
| DIFL | 22781 | 221.3 | 90.41 | 3.390 | 246.8 | 581.2 | -0.670 | 2.313 |
| Size | 22781 | 22.39 | 1.398 | 12.24 | 22.23 | 48.31 | 1.182 | 14.01 |
| Age | 22781 | 2.914 | 0.350 | 0.693 | 2.996 | 3.829 | -1.086 | 4.968 |
| lev | 22781 | 0.418 | 0.254 | -1.855 | 0.421 | 9.429 | 2.648 | 83.88 |
| Top1 | 22781 | 0.0920 | 0.166 | 0.00900 | 0.00900 | 0.961 | 1.985 | 6.055 |
| Mfee | 22781 | 0.211 | 14.02 | 0 | 0.0730 | 2115 | 150.5 | 22700 |
| Growth | 22781 | -0.298 | 0.903 | -13.39 | -0.129 | 57.41 | 15.77 | 824.7 |
| ROE | 22781 | 0.0490 | 0.231 | -7.016 | 0.0640 | 14.02 | 7.325 | 702.6 |
| TobinQ | 22781 | 0.213 | 0.838 | 0 | 0.00500 | 25.51 | 8.657 | 132.0 |
| Mshare | 22781 | 0.00800 | 0.0480 | 0 | 0 | 0.692 | 7.737 | 70.38 |
| Indep | 22781 | 0.375 | 0.0560 | 0 | 0.354 | 0.810 | 1.250 | 6.493 |
| Dual | 22781 | 0.232 | 0.422 | 0 | 0 | 1 | 1.268 | 2.607 |
| SOE | 22781 | 0.430 | 0.495 | 0 | 0 | 1 | 0.284 | 1.081 |
| Board | 22781 | 2.141 | 0.203 | 0 | 2.197 | 3.819 | -0.338 | 5.310 |

**Table 3 The impact of digital finance on enterprise green innovation: benchmark regression.**

|  | (1) | (2) | (3) |
| --- | --- | --- | --- |
|  | PAT | INPAT | UPAT |
| main |  |  |  |
| DIF | 0.2412^***^ | 0.2697^***^ | 0.1054^***^ |
|  | (3.3948) | (3.7023) | (2.9687) |
| 2011.year | 0.0000 | 0.0000 | 0.0000 |
|  | (.) | (.) | (.) |
| 2012.year | -5.8124^*^ | -6.7058^**^ | -2.4076 |
|  | (-1.7301) | (-2.1045) | (-1.4073) |
| 2013.year | -13.6271^**^ | -15.1802^***^ | -6.1261^**^ |
|  | (-2.2551) | (-2.6066) | (-2.0093) |
| 2014.year | -10.1259 | -13.7944^**^ | -4.2688 |
|  | (-1.4836) | (-2.1438) | (-1.2268) |
| 2015.year | -12.1784 | -18.6899^**^ | -4.1083 |
|  | (-1.3879) | (-2.2446) | (-0.9203) |
| 2016.year | -9.0948 | -17.6726^*^ | -3.1734 |
|  | (-0.9056) | (-1.8745) | (-0.6261) |
| 2017.year | -7.9624 | -18.5438^*^ | -2.6581 |
|  | (-0.6650) | (-1.6684) | (-0.4394) |
| 2018.year | -5.7761 | -18.8300 | -1.8276 |
|  | (-0.4509) | (-1.5831) | (-0.2806) |
| 2019.year | -5.3574 | -22.2371^*^ | 0.4733 |
|  | (-0.3877) | (-1.7097) | (0.0676) |
| 2020.year | -24.6916^*^ | -29.8259^**^ | -17.9638^**^ |
|  | (-1.6726) | (-2.1293) | (-2.3395) |
| 2021.year | -83.6081^***^ | -78.4912^***^ | -45.6096^***^ |
|  | (-4.2501) | (-3.9353) | (-4.4034) |
| 1.province | 0.0000 | 0.0000 | 0.0000 |
|  | (.) | (.) | (.) |
| 2.province | 14.1136 | 8.2069 | 5.5630 |
|  | (1.2913) | (0.9906) | (1.1059) |
| 3.province | -26.9839^***^ | -24.2115^***^ | -11.0327^***^ |
|  | (-3.5045) | (-3.3030) | (-2.8677) |
| 4.province | -29.5934^**^ | -23.8447^**^ | -8.9540 |
|  | (-2.4215) | (-2.2520) | (-1.5537) |
| 5.province | -15.2651^**^ | -13.3442^**^ | -6.6757^**^ |
|  | (-2.3905) | (-2.3494) | (-2.0895) |
| 6.province | -43.9617^***^ | -29.9300^***^ | -21.8141^***^ |
|  | (-3.4082) | (-2.7369) | (-3.2726) |
| 7.province | -14.8790^*^ | -7.4032 | -10.7122^*^ |
|  | (-1.7685) | (-1.0717) | (-1.9189) |
| 8.province | -53.8960^***^ | -44.4713^***^ | -26.5840^***^ |
|  | (-4.0864) | (-3.7384) | (-4.0626) |
| 9.province | -9.8038 | -6.3562 | -1.7233 |
|  | (-1.1997) | (-0.9262) | (-0.4142) |
| 10.province | -8.5126 | -10.0214 | -3.5670 |
|  | (-1.0448) | (-1.2655) | (-0.9834) |
| 11.province | -28.4348^***^ | -21.2112^**^ | -12.3620^**^ |
|  | (-2.6015) | (-2.2141) | (-2.1875) |
| 12.province | -19.8872^***^ | -13.9858^**^ | -8.3288^**^ |
|  | (-2.6335) | (-2.1712) | (-2.1768) |
| 13.province | -24.5002^***^ | -17.5084^***^ | -12.7308^***^ |
|  | (-3.0700) | (-2.5784) | (-2.9136) |
| 14.province | -43.1259^***^ | -32.8517^***^ | -20.2916^***^ |
|  | (-3.9989) | (-3.4490) | (-3.7088) |
| 15.province | -19.8762^***^ | -15.6602^***^ | -9.0856^***^ |
|  | (-3.0156) | (-2.6609) | (-2.7934) |
| 16.province | -17.1745^**^ | -16.8011^**^ | -6.1681 |
|  | (-2.1108) | (-2.1973) | (-1.4624) |
| 17.province | -25.6669^***^ | -19.4289^**^ | -10.8827^**^ |
|  | (-2.7013) | (-2.2772) | (-2.4316) |
| 18.province | -14.5732 | -10.1208 | -3.6820 |
|  | (-1.5025) | (-1.3498) | (-0.7318) |
| 19.province | -52.3583^***^ | -38.6610^***^ | -29.9047^***^ |
|  | (-2.9947) | (-2.6259) | (-2.9470) |
| 20.province | -25.8423^*^ | -14.5129 | -11.1241 |
|  | (-1.7855) | (-1.2955) | (-1.3273) |
| 21.province | -9.4113 | -8.3175 | -4.4154 |
|  | (-1.3772) | (-1.3981) | (-1.2710) |
| 22.province | -26.5315^***^ | -25.4110^***^ | -8.7947^*^ |
|  | (-2.7884) | (-2.7436) | (-1.9272) |
| 23.province | -20.4630^**^ | -21.9146^***^ | -8.6036^*^ |
|  | (-2.4526) | (-2.7094) | (-1.9101) |
| 24.province | -29.7060^***^ | -23.8453^***^ | -14.1116^***^ |
|  | (-3.7251) | (-3.3821) | (-3.4529) |
| 25.province | -15.0444^**^ | -9.9770 | -7.8207^**^ |
|  | (-2.0186) | (-1.5939) | (-2.0202) |
| 26.province | -30.5928^***^ | -23.7774^***^ | -11.0974^**^ |
|  | (-3.0972) | (-2.7069) | (-2.2458) |
| 27.province | -68.8239^***^ | -49.7654^***^ | -39.6358^***^ |
|  | (-3.1236) | (-2.6388) | (-3.7015) |
| 28.province | -26.7052^*^ | -20.0155 | -10.0246 |
|  | (-1.6479) | (-1.5824) | (-1.2761) |
| 29.province | -16.5681^*^ | -10.6058 | -12.0581^**^ |
|  | (-1.8477) | (-1.2361) | (-2.4506) |
| 30.province | -23.9440^***^ | -20.5936^***^ | -10.7506^***^ |
|  | (-3.8061) | (-3.5945) | (-3.2462) |
| 31.province | -23.3751^**^ | -18.1818^**^ | -11.0838^**^ |
|  | (-2.3080) | (-2.0011) | (-2.0553) |
| _cons | -50.1964^***^ | -56.6347^***^ | -27.8336^***^ |
|  | (-4.8795) | (-4.6024) | (-5.0066) |
| / |  |  |  |
| var(e.PAT) | 5.4e+03^***^ |  |  |
|  | (3.0495) |  |  |
| var(e.INPAT) |  | 3.3e+03^**^ |  |
|  |  | (2.5080) |  |
| var(e.UPAT) |  |  | 1.2e+03^***^ |
|  |  |  | (3.2057) |
| *N* | 22781 | 22781 | 22781 |
| pseudo *R*^2^ | 0.0128 | 0.0141 | 0.0190 |

*t* statistics in parentheses

^*^ *p* < 0.1, ^**^ *p* < 0.05, ^***^ *p* < 0.01

|  | (1) | (2) | (3) |
| --- | --- | --- | --- |
|  | PAT | INPAT | UPAT |
| main |  |  |  |
| DIF | 0.1412^**^ | 0.2053^***^ | 0.0486 |
|  | (2.2559) | (3.2001) | (1.5843) |
| Size | 19.0160^***^ | 15.5699^***^ | 8.2557^***^ |
|  | (4.9287) | (4.2377) | (5.3600) |
| Age | -29.6241^***^ | -20.7352^***^ | -14.6130^***^ |
|  | (-3.7899) | (-3.6727) | (-3.6221) |
| lev | -2.3339 | -8.6100 | 7.4236^*^ |
|  | (-0.2669) | (-1.1429) | (1.9093) |
| Top1 | 6.6467 | -8.9407 | 15.4544^**^ |
|  | (0.3963) | (-0.6518) | (2.0009) |
| Mfee | -12.1058^**^ | -5.2157 | -8.1610^**^ |
|  | (-2.2065) | (-1.5865) | (-2.3993) |
| Growth | 0.5067 | 0.6769 | -0.3611 |
|  | (0.4767) | (0.7370) | (-0.6745) |
| ROE | 5.6204 | 4.2247 | 4.6433^**^ |
|  | (1.5890) | (1.4180) | (2.4021) |
| TobinQ | 3.3534 | 2.3588 | 1.1433 |
|  | (1.0608) | (1.1141) | (0.5866) |
| Mshare | -1.9191 | 7.9416 | -5.1134 |
|  | (-0.1018) | (0.5649) | (-0.4311) |
| Indep | 43.2382 | 27.8085 | 23.7394 |
|  | (1.4495) | (1.3230) | (1.4632) |
| Dual | 4.8229^*^ | 4.6548^**^ | 1.1462 |
|  | (1.7508) | (2.1169) | (0.8807) |
| SOE | 1.5075 | 2.7335 | -0.7720 |
|  | (0.5505) | (1.1431) | (-0.6119) |
| Board | 4.5568 | 8.3589 | -1.8162 |
|  | (0.5237) | (1.0525) | (-0.5076) |
| 2011.year | 0.0000 | 0.0000 | 0.0000 |
|  | (.) | (.) | (.) |
| 2012.year | -0.7657 | -3.4575 | 0.3519 |
|  | (-0.2383) | (-1.1940) | (0.2169) |
| 2013.year | -4.3118 | -9.4325^*^ | -0.9144 |
|  | (-0.7666) | (-1.8134) | (-0.3249) |
| 2014.year | -0.0372 | -7.7258 | 1.4596 |
|  | (-0.0056) | (-1.3169) | (0.4295) |
| 2015.year | -0.5296 | -12.0008 | 2.8608 |
|  | (-0.0624) | (-1.5802) | (0.6525) |
| 2016.year | 2.8571 | -11.4745 | 4.2091 |
|  | (0.2847) | (-1.2996) | (0.8243) |
| 2017.year | 5.7325 | -11.5189 | 5.8770 |
|  | (0.4787) | (-1.1041) | (0.9659) |
| 2018.year | 9.1035 | -10.9583 | 7.2863 |
|  | (0.7083) | (-0.9821) | (1.1113) |
| 2019.year | 11.5457 | -13.4932 | 10.8026 |
|  | (0.8327) | (-1.1104) | (1.5088) |
| 2020.year | -7.0527 | -20.4707 | -7.2912 |
|  | (-0.4894) | (-1.5650) | (-1.0301) |
| 2021.year | -71.6976^***^ | -70.5940^***^ | -38.9985^***^ |
|  | (-3.7626) | (-3.8203) | (-3.8400) |
| 1.province | 0.0000 | 0.0000 | 0.0000 |
|  | (.) | (.) | (.) |
| 2.province | 4.5110 | -0.0236 | 0.8864 |
|  | (0.5238) | (-0.0036) | (0.2360) |
| 3.province | -20.0757^***^ | -19.2511^***^ | -7.4310^**^ |
|  | (-2.8169) | (-2.8787) | (-2.1374) |
| 4.province | -24.0476^**^ | -18.9759^**^ | -6.2191 |
|  | (-2.4006) | (-2.0914) | (-1.3283) |
| 5.province | -9.6998^*^ | -9.0053^*^ | -4.2569 |
|  | (-1.6551) | (-1.8072) | (-1.4332) |
| 6.province | -38.8438^***^ | -25.5330^***^ | -20.4203^***^ |
|  | (-4.0956) | (-3.1885) | (-4.0309) |
| 7.province | -19.0832^*^ | -10.5189 | -12.1275^**^ |
|  | (-1.8521) | (-1.2399) | (-2.0841) |
| 8.province | -34.4243^***^ | -29.1488^***^ | -17.2247^***^ |
|  | (-3.2964) | (-3.1084) | (-3.3353) |
| 9.province | -20.0913^**^ | -14.1130^**^ | -6.9964^*^ |
|  | (-2.4240) | (-2.0299) | (-1.7313) |
| 10.province | -9.6681 | -9.8824 | -4.2382 |
|  | (-1.2459) | (-1.2951) | (-1.2511) |
| 11.province | -26.3025^**^ | -19.6606^**^ | -11.6923^**^ |
|  | (-2.4939) | (-2.2975) | (-2.0551) |
| 12.province | -11.9848^*^ | -8.6601 | -3.8275 |
|  | (-1.7830) | (-1.5060) | (-1.1419) |
| 13.province | -17.1253^**^ | -12.0045^**^ | -9.0661^**^ |
|  | (-2.4234) | (-2.0229) | (-2.3751) |
| 14.province | -38.7495^***^ | -28.8766^***^ | -18.4854^***^ |
|  | (-3.7348) | (-3.2486) | (-3.5592) |
| 15.province | -12.6560^**^ | -10.0362^*^ | -5.6366^**^ |
|  | (-2.1070) | (-1.9028) | (-1.9625) |
| 16.province | -17.5116^**^ | -17.8085^**^ | -5.8051 |
|  | (-2.2829) | (-2.4708) | (-1.5072) |
| 17.province | -27.6874^***^ | -21.5523^***^ | -11.6141^***^ |
|  | (-3.1082) | (-2.7321) | (-2.7455) |
| 18.province | -27.6980^***^ | -21.2318^***^ | -8.9981^**^ |
|  | (-3.2754) | (-3.0087) | (-2.1615) |
| 19.province | -38.0540^**^ | -26.9501^**^ | -23.0924^***^ |
|  | (-2.4858) | (-2.0891) | (-2.6888) |
| 20.province | -33.2457^***^ | -19.3537^**^ | -15.5930^**^ |
|  | (-3.2687) | (-2.3410) | (-2.5399) |
| 21.province | -5.9840 | -5.9342 | -2.4033 |
|  | (-0.9626) | (-1.0858) | (-0.7878) |
| 22.province | -34.6331^***^ | -32.2359^***^ | -13.4410^***^ |
|  | (-3.9901) | (-3.6612) | (-3.4169) |
| 23.province | -13.7401^*^ | -17.5465^***^ | -5.3431 |
|  | (-1.9285) | (-2.7612) | (-1.3620) |
| 24.province | -26.2000^***^ | -23.1235^***^ | -11.3854^***^ |
|  | (-3.4925) | (-3.3219) | (-3.1729) |
| 25.province | -6.5726 | -4.2358 | -3.4817 |
|  | (-1.0263) | (-0.7987) | (-1.0576) |
| 26.province | -31.5613^***^ | -26.0439^***^ | -11.0672^**^ |
|  | (-3.3352) | (-3.1242) | (-2.4064) |
| 27.province | -59.7091^***^ | -42.1920^***^ | -34.6032^***^ |
|  | (-3.4537) | (-2.7554) | (-4.0171) |
| 28.province | -30.6539^**^ | -24.1407^**^ | -11.9742^*^ |
|  | (-2.1451) | (-2.1314) | (-1.7798) |
| 29.province | -19.4107^**^ | -13.9693^*^ | -12.9920^***^ |
|  | (-2.4148) | (-1.7677) | (-3.2989) |
| 30.province | -17.7869^***^ | -15.7789^***^ | -7.7140^***^ |
|  | (-3.1819) | (-3.1819) | (-2.6612) |
| 31.province | -23.4799^**^ | -18.1570^**^ | -11.7263^**^ |
|  | (-2.5302) | (-2.2401) | (-2.3416) |
| _cons | -4.1e+02^***^ | -3.7e+02^***^ | -1.8e+02^***^ |
|  | (-4.9805) | (-4.2052) | (-5.8529) |
| / |  |  |  |
| var(e.PAT) | 4.8e+03^***^ |  |  |
|  | (3.0766) |  |  |
| var(e.INPAT) |  | 3.0e+03^**^ |  |
|  |  | (2.5009) |  |
| var(e.UPAT) |  |  | 978.8469^***^ |
|  |  |  | (3.3472) |
| *N* | 22781 | 22781 | 22781 |
| pseudo *R*^2^ | 0.0310 | 0.0347 | 0.0451 |

*t* statistics in parentheses

^*^ *p* < 0.1, ^**^ *p* < 0.05, ^***^ *p* < 0.01

**Table 4 The impact of digital finance development on enterprise green innovation: indicator dimensionality reduction.**

|  | (1) | (2) | (3) |
| --- | --- | --- | --- |
|  | PAT | INPAT | UPAT |
| main |  |  |  |
| DIFB | 0.1136^**^ | 0.1549^***^ | 0.0439^*^ |
|  | (2.3816) | (3.1593) | (1.8865) |
| Size | 19.0226^***^ | 15.5815^***^ | 8.2564^***^ |
|  | (4.9328) | (4.2417) | (5.3640) |
| Age | -29.5860^***^ | -20.6837^***^ | -14.5989^***^ |
|  | (-3.7850) | (-3.6673) | (-3.6184) |
| lev | -2.4549 | -8.7101 | 7.3486^*^ |
|  | (-0.2798) | (-1.1520) | (1.8866) |
| Top1 | 6.4467 | -9.1658 | 15.3625^**^ |
|  | (0.3837) | (-0.6663) | (1.9885) |
| Mfee | -12.1313^**^ | -5.2231 | -8.1884^**^ |
|  | (-2.2069) | (-1.5888) | (-2.4039) |
| Growth | 0.4853 | 0.6573 | -0.3732 |
|  | (0.4562) | (0.7160) | (-0.6945) |
| ROE | 5.5103 | 4.0658 | 4.6075^**^ |
|  | (1.5622) | (1.3677) | (2.3862) |
| TobinQ | 3.3624 | 2.3675 | 1.1497 |
|  | (1.0639) | (1.1193) | (0.5897) |
| Mshare | -1.5048 | 8.4998 | -4.9440 |
|  | (-0.0797) | (0.6023) | (-0.4161) |
| Indep | 43.2347 | 27.8538 | 23.7672 |
|  | (1.4496) | (1.3257) | (1.4648) |
| Dual | 4.8057^*^ | 4.6352^**^ | 1.1362 |
|  | (1.7474) | (2.1123) | (0.8742) |
| SOE | 1.5325 | 2.7679 | -0.7609 |
|  | (0.5594) | (1.1559) | (-0.6030) |
| Board | 4.5843 | 8.3805 | -1.7858 |
|  | (0.5269) | (1.0551) | (-0.4995) |
| 2011.year | 0.0000 | 0.0000 | 0.0000 |
|  | (.) | (.) | (.) |
| 2012.year | 0.8351 | -0.6816 | 0.6900 |
|  | (0.3317) | (-0.3091) | (0.5374) |
| 2013.year | -0.2846 | -2.8176 | 0.1207 |
|  | (-0.0742) | (-0.8350) | (0.0626) |
| 2014.year | 2.1163 | -3.4947 | 1.6897 |
|  | (0.3819) | (-0.7398) | (0.5963) |
| 2015.year | 3.5538 | -4.7650 | 3.6621 |
|  | (0.5432) | (-0.8555) | (1.0669) |
| 2016.year | 8.4571 | -1.9178 | 5.4725 |
|  | (1.1165) | (-0.3079) | (1.4086) |
| 2017.year | 13.3862 | 1.2443 | 7.7544^*^ |
|  | (1.5383) | (0.1776) | (1.7394) |
| 2018.year | 16.6152^*^ | 1.8004 | 9.0252^*^ |
|  | (1.7317) | (0.2327) | (1.8340) |
| 2019.year | 18.9785^*^ | -0.6400 | 12.4206^**^ |
|  | (1.7902) | (-0.0741) | (2.2397) |
| 2020.year | 0.3509 | -7.5183 | -5.7494 |
|  | (0.0319) | (-0.7933) | (-1.0812) |
| 2021.year | -65.0115^***^ | -58.2581^***^ | -37.9064^***^ |
|  | (-3.9997) | (-3.8676) | (-4.1987) |
| 1.province | 0.0000 | 0.0000 | 0.0000 |
|  | (.) | (.) | (.) |
| 2.province | 3.7803 | -0.6357 | 0.4142 |
|  | (0.4390) | (-0.0985) | (0.1095) |
| 3.province | -20.6989^***^ | -19.8861^***^ | -7.7823^**^ |
|  | (-2.8763) | (-2.9305) | (-2.2235) |
| 4.province | -25.8386^**^ | -21.6322^**^ | -6.8333 |
|  | (-2.5628) | (-2.3364) | (-1.4559) |
| 5.province | -10.7151^*^ | -10.1222^**^ | -4.8056 |
|  | (-1.8135) | (-1.9834) | (-1.6002) |
| 6.province | -39.5348^***^ | -26.6572^***^ | -20.6297^***^ |
|  | (-4.1657) | (-3.3063) | (-4.0775) |
| 7.province | -20.5733^**^ | -12.7060 | -12.6569^**^ |
|  | (-1.9938) | (-1.4876) | (-2.1765) |
| 8.province | -36.0506^***^ | -31.3121^***^ | -17.9098^***^ |
|  | (-3.4087) | (-3.2557) | (-3.4331) |
| 9.province | -20.7997^**^ | -15.3703^**^ | -7.1515^*^ |
|  | (-2.5189) | (-2.2031) | (-1.7784) |
| 10.province | -10.7787 | -11.5264 | -4.6338 |
|  | (-1.3808) | (-1.4866) | (-1.3685) |
| 11.province | -27.1130^**^ | -20.9587^**^ | -11.9289^**^ |
|  | (-2.5754) | (-2.4388) | (-2.1040) |
| 12.province | -12.4792^*^ | -9.2686 | -4.0584 |
|  | (-1.8441) | (-1.5972) | (-1.2037) |
| 13.province | -17.5529^**^ | -12.6523^**^ | -9.2306^**^ |
|  | (-2.4775) | (-2.1152) | (-2.4145) |
| 14.province | -39.8245^***^ | -30.5807^***^ | -18.8159^***^ |
|  | (-3.8382) | (-3.4019) | (-3.6287) |
| 15.province | -13.2380^**^ | -10.6047^**^ | -5.9829^**^ |
|  | (-2.1759) | (-1.9758) | (-2.0632) |
| 16.province | -17.5815^**^ | -17.9954^**^ | -5.8008 |
|  | (-2.2951) | (-2.4981) | (-1.5078) |
| 17.province | -28.5508^***^ | -22.7972^***^ | -11.9231^***^ |
|  | (-3.1953) | (-2.8533) | (-2.8154) |
| 18.province | -29.2139^***^ | -23.5711^***^ | -9.5131^**^ |
|  | (-3.4352) | (-3.2507) | (-2.2898) |
| 19.province | -40.2058^***^ | -30.0310^**^ | -23.8434^***^ |
|  | (-2.6177) | (-2.3019) | (-2.7720) |
| 20.province | -34.9043^***^ | -22.0016^***^ | -16.0993^***^ |
|  | (-3.4296) | (-2.6192) | (-2.6398) |
| 21.province | -6.6455 | -6.8979 | -2.6449 |
|  | (-1.0691) | (-1.2544) | (-0.8675) |
| 22.province | -36.1553^***^ | -34.4779^***^ | -13.9645^***^ |
|  | (-4.1490) | (-3.8343) | (-3.5560) |
| 23.province | -15.0871^**^ | -19.4110^***^ | -5.8806 |
|  | (-2.0925) | (-2.9649) | (-1.4822) |
| 24.province | -25.9032^***^ | -22.2565^***^ | -11.4881^***^ |
|  | (-3.4724) | (-3.2464) | (-3.2313) |
| 25.province | -7.1253 | -5.0856 | -3.6653 |
|  | (-1.1134) | (-0.9571) | (-1.1152) |
| 26.province | -32.0176^***^ | -26.6565^***^ | -11.2589^**^ |
|  | (-3.3736) | (-3.1750) | (-2.4452) |
| 27.province | -61.0874^***^ | -44.1636^***^ | -35.1216^***^ |
|  | (-3.5161) | (-2.8507) | (-4.0696) |
| 28.province | -32.5935^**^ | -26.9245^**^ | -12.6884^*^ |
|  | (-2.2746) | (-2.3311) | (-1.8872) |
| 29.province | -20.4380^**^ | -15.4531^*^ | -13.3729^***^ |
|  | (-2.5114) | (-1.9187) | (-3.3670) |
| 30.province | -17.7541^***^ | -15.3901^***^ | -7.8626^***^ |
|  | (-3.1797) | (-3.1154) | (-2.7238) |
| 31.province | -24.0966^***^ | -19.1369^**^ | -11.9102^**^ |
|  | (-2.5947) | (-2.3468) | (-2.3793) |
| _cons | -4.1e+02^***^ | -3.6e+02^***^ | -1.8e+02^***^ |
|  | (-4.9624) | (-4.1887) | (-5.8423) |
| / |  |  |  |
| var(e.PAT) | 4.8e+03^***^ |  |  |
|  | (3.0768) |  |  |
| var(e.INPAT) |  | 3.0e+03^**^ |  |
|  |  | (2.5014) |  |
| var(e.UPAT) |  |  | 978.8676^***^ |
|  |  |  | (3.3469) |
| *N* | 22781 | 22781 | 22781 |
| pseudo *R*^2^ | 0.0311 | 0.0347 | 0.0452 |

*t* statistics in parentheses

^*^ *p* < 0.1, ^**^ *p* < 0.05, ^***^ *p* < 0.01

|  | (1) | (2) | (3) |
| --- | --- | --- | --- |
|  | PAT | INPAT | UPAT |
| main |  |  |  |
| DIFD | 0.0917 | 0.1567^***^ | 0.0242 |
|  | (1.5471) | (2.7426) | (0.8178) |
| Size | 19.0362^***^ | 15.5838^***^ | 8.2658^***^ |
|  | (4.9279) | (4.2359) | (5.3612) |
| Age | -29.6942^***^ | -20.8276^***^ | -14.6324^***^ |
|  | (-3.7988) | (-3.6820) | (-3.6276) |
| lev | -1.9633 | -8.1122 | 7.5681^*^ |
|  | (-0.2256) | (-1.0863) | (1.9506) |
| Top1 | 7.0092 | -8.4691 | 15.5719^**^ |
|  | (0.4186) | (-0.6197) | (2.0167) |
| Mfee | -11.9972^**^ | -5.1300 | -8.0927^**^ |
|  | (-2.1963) | (-1.5808) | (-2.3888) |
| Growth | 0.5648 | 0.7437 | -0.3371 |
|  | (0.5308) | (0.8057) | (-0.6320) |
| ROE | 5.6170 | 4.2876 | 4.6211^**^ |
|  | (1.5840) | (1.4332) | (2.3946) |
| TobinQ | 3.3223 | 2.3191 | 1.1304 |
|  | (1.0513) | (1.0968) | (0.5805) |
| Mshare | -1.9750 | 7.7632 | -5.1862 |
|  | (-0.1051) | (0.5550) | (-0.4386) |
| Indep | 43.1287 | 27.6745 | 23.6497 |
|  | (1.4455) | (1.3155) | (1.4579) |
| Dual | 4.8744^*^ | 4.7271^**^ | 1.1657 |
|  | (1.7669) | (2.1436) | (0.8941) |
| SOE | 1.4923 | 2.7197 | -0.7790 |
|  | (0.5449) | (1.1375) | (-0.6173) |
| Board | 4.4689 | 8.2774 | -1.8732 |
|  | (0.5134) | (1.0423) | (-0.5227) |
| 2011.year | 0.0000 | 0.0000 | 0.0000 |
|  | (.) | (.) | (.) |
| 2012.year | 1.4867 | -1.2498 | 1.4712 |
|  | (0.4697) | (-0.4496) | (0.9105) |
| 2013.year | -0.4145 | -5.8770 | 1.0940 |
|  | (-0.0731) | (-1.1620) | (0.3796) |
| 2014.year | 6.6861 | 0.1799 | 4.3651 |
|  | (1.2149) | (0.0391) | (1.5295) |
| 2015.year | 8.3825 | -1.4022 | 6.6728^*^ |
|  | (1.2317) | (-0.2452) | (1.8593) |
| 2016.year | 10.7095 | -3.4057 | 7.9779 |
|  | (1.1210) | (-0.4237) | (1.6238) |
| 2017.year | 12.7076 | -6.0307 | 9.7547 |
|  | (0.9897) | (-0.5515) | (1.4881) |
| 2018.year | 18.6415 | -1.5773 | 11.9994^*^ |
|  | (1.4747) | (-0.1494) | (1.8509) |
| 2019.year | 22.2500^*^ | -2.6436 | 15.9890^**^ |
|  | (1.6702) | (-0.2380) | (2.2912) |
| 2020.year | 4.3497 | -8.8735 | -1.7895 |
|  | (0.3231) | (-0.7560) | (-0.2687) |
| 2021.year | -57.7934^***^ | -55.7062^***^ | -32.5147^***^ |
|  | (-3.3791) | (-3.5358) | (-3.5443) |
| 1.province | 0.0000 | 0.0000 | 0.0000 |
|  | (.) | (.) | (.) |
| 2.province | 6.8515 | 2.8926 | 1.8561 |
|  | (0.8010) | (0.4369) | (0.5056) |
| 3.province | -18.4665^***^ | -17.0921^***^ | -6.8067^**^ |
|  | (-2.6262) | (-2.6229) | (-1.9803) |
| 4.province | -21.8362^**^ | -14.7439 | -5.7686 |
|  | (-2.1216) | (-1.6143) | (-1.1949) |
| 5.province | -7.3234 | -5.7177 | -3.3470 |
|  | (-1.2830) | (-1.2111) | (-1.1576) |
| 6.province | -38.2778^***^ | -24.1288^***^ | -20.3736^***^ |
|  | (-3.9930) | (-3.0008) | (-3.9767) |
| 7.province | -17.2164 | -6.9701 | -11.7211^**^ |
|  | (-1.6319) | (-0.8029) | (-1.9749) |
| 8.province | -31.7421^***^ | -24.8893^***^ | -16.4075^***^ |
|  | (-3.0491) | (-2.7198) | (-3.1803) |
| 9.province | -20.1897^**^ | -13.5450^*^ | -7.2392^*^ |
|  | (-2.3877) | (-1.9158) | (-1.7583) |
| 10.province | -8.2208 | -7.1171 | -3.9213 |
|  | (-1.0422) | (-0.9309) | (-1.1282) |
| 11.province | -25.5827^**^ | -17.9882^**^ | -11.6507^**^ |
|  | (-2.3861) | (-2.0863) | (-2.0139) |
| 12.province | -10.9231 | -7.0427 | -3.4766 |
|  | (-1.6347) | (-1.2370) | (-1.0450) |
| 13.province | -16.7120^**^ | -11.1503^*^ | -8.9629^**^ |
|  | (-2.3599) | (-1.8846) | (-2.3432) |
| 14.province | -38.1807^***^ | -27.3172^***^ | -18.4970^***^ |
|  | (-3.6162) | (-3.0435) | (-3.5039) |
| 15.province | -11.1469^*^ | -8.1075 | -5.0112^*^ |
|  | (-1.8753) | (-1.5608) | (-1.7794) |
| 16.province | -17.5799^**^ | -17.7195^**^ | -5.8737 |
|  | (-2.2769) | (-2.4366) | (-1.5189) |
| 17.province | -26.8364^***^ | -19.8925^**^ | -11.4254^***^ |
|  | (-2.9852) | (-2.5299) | (-2.6724) |
| 18.province | -25.6463^***^ | -17.2817^**^ | -8.5976^**^ |
|  | (-2.8933) | (-2.4044) | (-1.9647) |
| 19.province | -36.2530^**^ | -23.2067^*^ | -22.7950^***^ |
|  | (-2.3471) | (-1.8074) | (-2.6292) |
| 20.province | -32.1184^***^ | -16.4857^**^ | -15.5249^**^ |
|  | (-3.0596) | (-1.9788) | (-2.4533) |
| 21.province | -5.2566 | -4.5600 | -2.2413 |
|  | (-0.8312) | (-0.8259) | (-0.7239) |
| 22.province | -32.8659^***^ | -28.7904^***^ | -13.0786^***^ |
|  | (-3.6767) | (-3.2617) | (-3.2049) |
| 23.province | -11.6830 | -14.0393^**^ | -4.7697 |
|  | (-1.6355) | (-2.2519) | (-1.2128) |
| 24.province | -25.0953^***^ | -22.4851^***^ | -10.7031^***^ |
|  | (-3.3827) | (-3.2750) | (-2.9803) |
| 25.province | -6.1016 | -3.2424 | -3.4087 |
|  | (-0.9392) | (-0.6040) | (-1.0229) |
| 26.province | -30.8660^***^ | -24.9408^***^ | -10.8496^**^ |
|  | (-3.2600) | (-3.0093) | (-2.3563) |
| 27.province | -57.8358^***^ | -38.7804^**^ | -34.1601^***^ |
|  | (-3.3249) | (-2.5449) | (-3.9335) |
| 28.province | -28.3311^**^ | -19.9128^*^ | -11.4197^*^ |
|  | (-1.9604) | (-1.7672) | (-1.6750) |
| 29.province | -18.2697^**^ | -11.8005 | -12.7079^***^ |
|  | (-2.2867) | (-1.5330) | (-3.2124) |
| 30.province | -16.9693^***^ | -15.2804^***^ | -7.2015^**^ |
|  | (-3.0466) | (-3.1141) | (-2.4765) |
| 31.province | -23.0799^**^ | -17.1614^**^ | -11.7067^**^ |
|  | (-2.4671) | (-2.1090) | (-2.3217) |
| _cons | -4.1e+02^***^ | -3.6e+02^***^ | -1.7e+02^***^ |
|  | (-4.9837) | (-4.2102) | (-5.8385) |
| / |  |  |  |
| var(e.PAT) | 4.8e+03^***^ |  |  |
|  | (3.0771) |  |  |
| var(e.INPAT) |  | 3.0e+03^**^ |  |
|  |  | (2.5011) |  |
| var(e.UPAT) |  |  | 978.8877^***^ |
|  |  |  | (3.3479) |
| *N* | 22781 | 22781 | 22781 |
| pseudo *R*^2^ | 0.0310 | 0.0345 | 0.0451 |

*t* statistics in parentheses

^*^ *p* < 0.1, ^**^ *p* < 0.05, ^***^ *p* < 0.01

|  | (1) | (2) | (3) |
| --- | --- | --- | --- |
|  | PAT | INPAT | UPAT |
| main |  |  |  |
| DIFL | 0.0071 | 0.0354 | -0.0136 |
|  | (0.2329) | (1.3041) | (-0.9605) |
| Size | 19.0630^***^ | 15.6056^***^ | 8.2790^***^ |
|  | (4.9328) | (4.2402) | (5.3691) |
| Age | -29.7327^***^ | -20.8873^***^ | -14.6380^***^ |
|  | (-3.8041) | (-3.6905) | (-3.6300) |
| lev | -1.7551 | -7.7335 | 7.6272^**^ |
|  | (-0.2019) | (-1.0422) | (1.9648) |
| Top1 | 7.0509 | -8.3882 | 15.5640^**^ |
|  | (0.4210) | (-0.6138) | (2.0156) |
| Mfee | -11.8498^**^ | -4.9656 | -8.0434^**^ |
|  | (-2.1838) | (-1.6021) | (-2.3843) |
| Growth | 0.5937 | 0.7855 | -0.3270 |
|  | (0.5565) | (0.8466) | (-0.6119) |
| ROE | 5.4658 | 4.1260 | 4.5379^**^ |
|  | (1.5459) | (1.3805) | (2.3604) |
| TobinQ | 3.3042 | 2.2921 | 1.1238 |
|  | (1.0445) | (1.0822) | (0.5769) |
| Mshare | -1.5981 | 8.0528 | -5.0254 |
|  | (-0.0852) | (0.5782) | (-0.4259) |
| Indep | 42.8817 | 27.3079 | 23.5608 |
|  | (1.4379) | (1.3000) | (1.4525) |
| Dual | 4.8773^*^ | 4.7234^**^ | 1.1696 |
|  | (1.7678) | (2.1438) | (0.8969) |
| SOE | 1.4758 | 2.6856 | -0.7751 |
|  | (0.5387) | (1.1235) | (-0.6134) |
| Board | 4.3083 | 7.9985 | -1.9119 |
|  | (0.4948) | (1.0086) | (-0.5333) |
| 2011.year | 0.0000 | 0.0000 | 0.0000 |
|  | (.) | (.) | (.) |
| 2012.year | 5.3809^**^ | 4.1446^*^ | 3.3457^***^ |
|  | (2.2684) | (1.8677) | (2.8361) |
| 2013.year | 7.0575 | 3.9834 | 5.0127^**^ |
|  | (1.6277) | (1.0222) | (2.4047) |
| 2014.year | 13.2790^***^ | 8.7403^**^ | 7.9165^***^ |
|  | (2.8733) | (2.1490) | (3.4619) |
| 2015.year | 16.3966^**^ | 7.8179 | 11.7788^***^ |
|  | (2.4291) | (1.3327) | (3.5299) |
| 2016.year | 22.6572^***^ | 12.3559^*^ | 14.2287^***^ |
|  | (2.9989) | (1.9400) | (3.7654) |
| 2017.year | 29.5879^***^ | 17.7286^**^ | 17.6007^***^ |
|  | (3.4405) | (2.4117) | (4.1295) |
| 2018.year | 34.6548^***^ | 19.8584^**^ | 20.1734^***^ |
|  | (3.5447) | (2.4040) | (4.2074) |
| 2019.year | 39.1154^***^ | 20.0479^**^ | 24.5279^***^ |
|  | (3.8401) | (2.4160) | (4.6475) |
| 2020.year | 22.1367^**^ | 15.2928^*^ | 7.0599^*^ |
|  | (2.4219) | (1.9005) | (1.6793) |
| 2021.year | -38.5594^***^ | -29.1972^***^ | -23.1834^***^ |
|  | (-3.1011) | (-2.9113) | (-3.4577) |
| 1.province | 0.0000 | 0.0000 | 0.0000 |
|  | (.) | (.) | (.) |
| 2.province | 8.6849 | 5.9902 | 2.3679 |
|  | (1.0267) | (0.8891) | (0.6571) |
| 3.province | -17.7759^**^ | -15.8647^**^ | -6.6156^*^ |
|  | (-2.5311) | (-2.4509) | (-1.9278) |
| 4.province | -25.8597^**^ | -21.7118^**^ | -6.7918 |
|  | (-2.5586) | (-2.3649) | (-1.4422) |
| 5.province | -6.5062 | -4.2850 | -3.1377 |
|  | (-1.1550) | (-0.9242) | (-1.0971) |
| 6.province | -40.4328^***^ | -27.8201^***^ | -20.9306^***^ |
|  | (-4.2274) | (-3.3793) | (-4.1140) |
| 7.province | -20.4411^*^ | -12.5232 | -12.5424^**^ |
|  | (-1.9518) | (-1.4321) | (-2.1383) |
| 8.province | -33.2231^***^ | -27.4164^***^ | -16.8023^***^ |
|  | (-3.1908) | (-2.9536) | (-3.2639) |
| 9.province | -22.9340^***^ | -18.0940^**^ | -8.0550^**^ |
|  | (-2.7472) | (-2.5193) | (-1.9997) |
| 10.province | -10.6591 | -11.3106 | -4.5298 |
|  | (-1.3600) | (-1.4586) | (-1.3288) |
| 11.province | -28.2636^***^ | -22.6680^***^ | -12.3401^**^ |
|  | (-2.6512) | (-2.5947) | (-2.1533) |
| 12.province | -11.1919^*^ | -7.5320 | -3.5177 |
|  | (-1.6742) | (-1.3180) | (-1.0567) |
| 13.province | -17.4212^**^ | -12.2623^**^ | -9.1849^**^ |
|  | (-2.4565) | (-2.0491) | (-2.4033) |
| 14.province | -41.1301^***^ | -32.2287^***^ | -19.3707^***^ |
|  | (-3.9064) | (-3.4856) | (-3.7072) |
| 15.province | -10.1228^*^ | -6.3560 | -4.7500^*^ |
|  | (-1.6905) | (-1.2100) | (-1.7025) |
| 16.province | -18.2670^**^ | -18.9286^**^ | -6.0319 |
|  | (-2.3571) | (-2.5689) | (-1.5573) |
| 17.province | -28.4262^***^ | -22.4552^***^ | -11.8916^***^ |
|  | (-3.1678) | (-2.7962) | (-2.8016) |
| 18.province | -29.7432^***^ | -24.4829^***^ | -9.5584^**^ |
|  | (-3.4341) | (-3.3086) | (-2.2593) |
| 19.province | -40.6799^***^ | -30.2775^**^ | -24.1561^***^ |
|  | (-2.6741) | (-2.3483) | (-2.8114) |
| 20.province | -36.9902^***^ | -24.7621^***^ | -16.8168^***^ |
|  | (-3.6298) | (-2.9655) | (-2.7157) |
| 21.province | -6.5438 | -6.7463 | -2.6022 |
|  | (-1.0442) | (-1.2127) | (-0.8495) |
| 22.province | -36.2271^***^ | -34.4898^***^ | -13.9465^***^ |
|  | (-4.1075) | (-3.7809) | (-3.5059) |
| 23.province | -13.6679^*^ | -17.3811^***^ | -5.2710 |
|  | (-1.9242) | (-2.7302) | (-1.3492) |
| 24.province | -21.5081^***^ | -16.4193^***^ | -9.7059^***^ |
|  | (-3.1482) | (-2.7585) | (-2.9180) |
| 25.province | -7.4408 | -5.5576 | -3.7779 |
|  | (-1.1493) | (-1.0328) | (-1.1402) |
| 26.province | -31.3571^***^ | -25.7705^***^ | -10.9793^**^ |
|  | (-3.3041) | (-3.0814) | (-2.3820) |
| 27.province | -60.6276^***^ | -43.6373^***^ | -34.8822^***^ |
|  | (-3.4868) | (-2.8206) | (-4.0274) |
| 28.province | -31.6234^**^ | -25.4370^**^ | -12.3432^*^ |
|  | (-2.2062) | (-2.2197) | (-1.8318) |
| 29.province | -20.3357^**^ | -15.2786^**^ | -13.2474^***^ |
|  | (-2.5751) | (-1.9918) | (-3.3847) |
| 30.province | -14.2203^***^ | -10.4904^**^ | -6.5202^**^ |
|  | (-2.6948) | (-2.3307) | (-2.3792) |
| 31.province | -24.7550^***^ | -19.9772^**^ | -12.1749^**^ |
|  | (-2.6439) | (-2.4137) | (-2.4207) |
| _cons | -4.0e+02^***^ | -3.5e+02^***^ | -1.7e+02^***^ |
|  | (-4.9392) | (-4.1686) | (-5.7813) |
| / |  |  |  |
| var(e.PAT) | 4.8e+03^***^ |  |  |
|  | (3.0777) |  |  |
| var(e.INPAT) |  | 3.0e+03^**^ |  |
|  |  | (2.5021) |  |
| var(e.UPAT) |  |  | 978.9576^***^ |
|  |  |  | (3.3483) |
| *N* | 22781 | 22781 | 22781 |
| pseudo *R*^2^ | 0.0309 | 0.0343 | 0.0451 |

*t* statistics in parentheses

^*^ *p* < 0.1, ^**^ *p* < 0.05, ^***^ *p* < 0.01

**Table 5 Regression results of instrumental variables: provincial distance.**

|  | (1) | (2) | (3) |
| --- | --- | --- | --- |
|  | PAT | PAT | PAT |
| PAT |  |  |  |
| DIF | 0.1180^**^ |  | 0.0584^***^ |
|  | (2.0716) |  | (9.9834) |
| Size | 6.8610^***^ | 8.6032^***^ | 7.7626^***^ |
|  | (5.8554) | (9.6836) | (8.7265) |
| Age | -17.7492^***^ | -6.9383^***^ | -12.1880^***^ |
|  | (-3.1053) | (-4.6794) | (-6.9835) |
| lev | -0.3505 | -1.5197 | -0.9047 |
|  | (-0.1520) | (-0.6749) | (-0.4038) |
| Top1 | -4.7943 | -6.3128 | -5.1923 |
|  | (-1.1333) | (-1.5004) | (-1.2329) |
| Mfee | -4.2350^**^ | -4.0911^**^ | -4.2379^**^ |
|  | (-2.1644) | (-2.0763) | (-2.1657) |
| Growth | -0.1126 | -0.2624 | -0.1369 |
|  | (-0.2318) | (-0.5381) | (-0.2846) |
| ROE | 5.8103^***^ | 2.1382 | 4.2408^***^ |
|  | (2.6943) | (1.3514) | (2.6277) |
| TobinQ | -8.7966^***^ | -5.5988^***^ | -7.5154^***^ |
|  | (-5.8400) | (-7.5155) | (-8.7719) |
| Mshare | -7.8776 | -2.2404 | -3.5936 |
|  | (-0.8022) | (-0.2737) | (-0.4204) |
| Indep | 16.6394^*^ | 13.4423 | 15.1598 |
|  | (1.7401) | (1.4179) | (1.6008) |
| Dual | 1.6811 | 2.1475^**^ | 1.9233^**^ |
|  | (1.6411) | (2.2181) | (1.9698) |
| SOE | 1.2374 | -1.4396 | -0.1129 |
|  | (0.7488) | (-1.4145) | (-0.1070) |
| Board | 5.8764 | 0.5832 | 3.2029 |
|  | (1.4641) | (0.1961) | (1.0522) |
| distance |  | -0.0079^**^ | -0.0040 |
|  |  | (-2.1161) | (-1.0590) |
| _cons | -1.5e+02^***^ | -1.8e+02^***^ | -1.7e+02^***^ |
|  | (-6.6390) | (-10.5517) | (-9.5721) |
| DIF |  |  |  |
| Size | 15.1325^***^ |  |  |
|  | (16.0590) |  |  |
| Age | 93.3387^***^ |  |  |
|  | (34.4412) |  |  |
| lev | -9.3010^**^ |  |  |
|  | (-2.3798) |  |  |
| Top1 | -6.6799 |  |  |
|  | (-1.1295) |  |  |
| Mfee | -0.0525^***^ |  |  |
|  | (-11.2239) |  |  |
| Growth | -0.4078 |  |  |
|  | (-0.2659) |  |  |
| ROE | -26.3425^***^ |  |  |
|  | (-3.9229) |  |  |
| TobinQ | 21.5033^***^ |  |  |
|  | (14.5368) |  |  |
| Mshare | 71.9016^***^ |  |  |
|  | (3.9833) |  |  |
| Indep | -24.8329 |  |  |
|  | (-1.4672) |  |  |
| Dual | 4.0652^**^ |  |  |
|  | (2.2796) |  |  |
| SOE | -22.6645^***^ |  |  |
|  | (-12.9103) |  |  |
| Board | -44.8720^***^ |  |  |
|  | (-8.7669) |  |  |
| distance | -0.0664^***^ |  |  |
|  | (-9.8601) |  |  |
| _cons | -2.8e+02^***^ |  |  |
|  | (-12.7295) |  |  |
| / |  |  |  |
| athrho2_1 | -0.1537 |  |  |
|  | (-1.0588) |  |  |
| lnsigma1 | 3.1670^***^ |  |  |
|  | (72.0908) |  |  |
| lnsigma2 | 4.1067^***^ |  |  |
|  | (697.8699) |  |  |
| var(e.PAT) |  | 559.1980^***^ | 550.2754^***^ |
|  |  | (13.4279) | (13.3237) |
| *N* | 22781 | 22781 | 22781 |
| pseudo *R*^2^ |  | 0.0365 | 0.0398 |

*t* statistics in parentheses

^*^ *p* < 0.1, ^**^ *p* < 0.05, ^***^ *p* < 0.01

. xi: ivtobit PAT Size Age lev Top1 Mfee Growth ROE TobinQ Mshare Indep Dual SOE Board (DIF=distance), ll(0) ul(100) vce(cluste

> r id)

Fitting exogenous tobit model

Fitting full model

Iteration 0: log pseudolikelihood = -179530.21

Iteration 1: log pseudolikelihood = -179529.15

Iteration 2: log pseudolikelihood = -179527.3

Iteration 3: log pseudolikelihood = -179527.3

Iteration 4: log pseudolikelihood = -179527.3

Tobit model with endogenous regressors Number of obs = 22,781

Uncensored = 10,229

Limits: Lower = 0 Left-censored = 12,254

Upper = 100 Right-censored = 298

Wald chi2(14) = 229.41

Log pseudolikelihood = -179527.3 Prob > chi2 = 0.0000

(Std. err. adjusted for 2,071 clusters in id)

-----------------------------------------------------------------------------------

| Robust

| Coefficient std. err. z P>|z| [95% conf. interval]

------------------+----------------------------------------------------------------

DIF | .1180033 .0569633 2.07 0.038 .0063574 .2296492

Size | 6.861004 1.171747 5.86 0.000 4.564423 9.157586

Age | -17.74924 5.715813 -3.11 0.002 -28.95203 -6.546455

lev | -.3505031 2.305793 -0.15 0.879 -4.869773 4.168767

Top1 | -4.794275 4.230277 -1.13 0.257 -13.08547 3.496916

Mfee | -4.235007 1.956676 -2.16 0.030 -8.070021 -.3999925

Growth | -.1126416 .4859391 -0.23 0.817 -1.065065 .8397816

ROE | 5.81032 2.156524 2.69 0.007 1.583611 10.03703

TobinQ | -8.796618 1.506282 -5.84 0.000 -11.74888 -5.84436

Mshare | -7.877555 9.819916 -0.80 0.422 -27.12424 11.36913

Indep | 16.63939 9.562403 1.74 0.082 -2.10258 35.38135

Dual | 1.681105 1.024358 1.64 0.101 -.3266 3.68881

SOE | 1.237432 1.652594 0.75 0.454 -2.001593 4.476457

Board | 5.876394 4.013779 1.46 0.143 -1.990468 13.74326

_cons | -151.446 22.81159 -6.64 0.000 -196.1559 -106.7361

------------------+----------------------------------------------------------------

corr(e.DIF,e.PAT)| -.1524869 .1417798 -.4121381 .1300719

sd(e.PAT)| 23.73553 1.04271 21.77737 25.86977

sd(e.DIF)| 60.74672 .3574726 60.05011 61.45141

-----------------------------------------------------------------------------------

Instrumented: DIF

Instruments: Size Age lev Top1 Mfee Growth ROE TobinQ Mshare Indep Dual SOE

Board distance

Wald test of exogeneity (corr = 0): chi2(1) = 1.12 Prob > chi2 = 0.2897

.

. est store m1

.

. xi:tobit PAT distance Size Age lev Top1 Mfee Growth ROE TobinQ Mshare Indep Dual SOE Board ,vce(cluster id) ll(0) ul(100)

Refining starting values:

Grid node 0: log likelihood = -59789.584

Fitting full model:

Iteration 0: log pseudolikelihood = -59789.584

Iteration 1: log pseudolikelihood = -54880.146

Iteration 2: log pseudolikelihood = -53881.157

Iteration 3: log pseudolikelihood = -53833.892

Iteration 4: log pseudolikelihood = -53830.413

Iteration 5: log pseudolikelihood = -53829.886

Iteration 6: log pseudolikelihood = -53829.884

Tobit regression Number of obs = 22,781

Uncensored = 10,229

Limits: Lower = 0 Left-censored = 12,254

Upper = 100 Right-censored = 298

F(14, 22767) = 15.78

Prob > F = 0.0000

Log pseudolikelihood = -53829.884 Pseudo R2 = 0.0365

(Std. err. adjusted for 2,071 clusters in id)

------------------------------------------------------------------------------

| Robust

PAT | Coefficient std. err. t P>|t| [95% conf. interval]

-------------+----------------------------------------------------------------

distance | -.0079041 .0037352 -2.12 0.034 -.0152254 -.0005827

Size | 8.603186 .8884325 9.68 0.000 6.861798 10.34457

Age | -6.938305 1.482749 -4.68 0.000 -9.844594 -4.032016

lev | -1.51972 2.251702 -0.67 0.500 -5.93321 2.89377

Top1 | -6.312821 4.207289 -1.50 0.134 -14.55939 1.933752

Mfee | -4.091122 1.97035 -2.08 0.038 -7.953143 -.229101

Growth | -.2623675 .4875685 -0.54 0.591 -1.218035 .6933001

ROE | 2.138174 1.582201 1.35 0.177 -.9630474 5.239395

TobinQ | -5.598837 .7449707 -7.52 0.000 -7.05903 -4.138643

Mshare | -2.240365 8.183991 -0.27 0.784 -18.28155 13.80082

Indep | 13.44227 9.48059 1.42 0.156 -5.140338 32.02487

Dual | 2.147468 .9681471 2.22 0.027 .2498341 4.045103

SOE | -1.439555 1.017696 -1.41 0.157 -3.434308 .5551981

Board | .5832001 2.973563 0.20 0.845 -5.245186 6.411586

_cons | -182.4091 17.28725 -10.55 0.000 -216.2933 -148.525

-------------+----------------------------------------------------------------

var(e.PAT)| 559.198 41.64457 483.2497 647.0825

------------------------------------------------------------------------------

.

. est store m2

.

. xi:tobit PAT DIF distance Size Age lev Top1 Mfee Growth ROE TobinQ Mshare Indep Dual SOE Board ,vce(cluster id) ll(0) ul(100)

Refining starting values:

Grid node 0: log likelihood = -59689.904

Fitting full model:

Iteration 0: log pseudolikelihood = -59689.904

Iteration 1: log pseudolikelihood = -54703.184

Iteration 2: log pseudolikelihood = -53703.226

Iteration 3: log pseudolikelihood = -53651.939

Iteration 4: log pseudolikelihood = -53648.056

Iteration 5: log pseudolikelihood = -53647.431

Iteration 6: log pseudolikelihood = -53647.43

Tobit regression Number of obs = 22,781

Uncensored = 10,229

Limits: Lower = 0 Left-censored = 12,254

Upper = 100 Right-censored = 298

F(15, 22766) = 27.94

Prob > F = 0.0000

Log pseudolikelihood = -53647.43 Pseudo R2 = 0.0398

(Std. err. adjusted for 2,071 clusters in id)

------------------------------------------------------------------------------

| Robust

PAT | Coefficient std. err. t P>|t| [95% conf. interval]

-------------+----------------------------------------------------------------

DIF | .0584222 .0058519 9.98 0.000 .0469519 .0698924

distance | -.0039549 .0037346 -1.06 0.290 -.011275 .0033652

Size | 7.762618 .8895497 8.73 0.000 6.019039 9.506196

Age | -12.18801 1.74527 -6.98 0.000 -15.60886 -8.767165

lev | -.9046565 2.240312 -0.40 0.686 -5.295822 3.486509

Top1 | -5.192269 4.211489 -1.23 0.218 -13.44708 3.062537

Mfee | -4.237885 1.956784 -2.17 0.030 -8.073315 -.4024549

Growth | -.1369426 .4812486 -0.28 0.776 -1.080223 .8063375

ROE | 4.240831 1.613866 2.63 0.009 1.077544 7.404117

TobinQ | -7.515426 .8567609 -8.77 0.000 -9.194736 -5.836116

Mshare | -3.593561 8.546999 -0.42 0.674 -20.34626 13.15914

Indep | 15.15981 9.470075 1.60 0.109 -3.402182 33.7218

Dual | 1.923315 .9764209 1.97 0.049 .0094637 3.837167

SOE | -.112943 1.055894 -0.11 0.915 -2.182568 1.956682

Board | 3.202868 3.043837 1.05 0.293 -2.76326 9.168996

_cons | -167.9654 17.54731 -9.57 0.000 -202.3593 -133.5715

-------------+----------------------------------------------------------------

var(e.PAT)| 550.2754 41.30062 474.9963 637.485

------------------------------------------------------------------------------

.

. est store m3

.

. esttab m1 m2 m3 using distance.rtf, replace pr2(%6.4f) compress nogap b(%6.4f) t(%6.4f) star(* 0.1 ** 0.05 *** 0.01)

(file distance.rtf not found)

(output written to distance.rtf)

.

.

.

. ******Anderson-Rubin test

.

. weakiv ivtobit PAT (DIF=distance), ll(0)

Estimating model for Wald tests using ivtobit...

Estimating confidence sets over 100 grid points

----+--- 1 ---+--- 2 ---+--- 3 ---+--- 4 ---+--- 5

.................................................. 50

.................................................. 100

Weak instrument robust tests and confidence sets for IV tobit

H0: beta[PAT:DIF] = 0

------------------------------------------------------------------------------

Test | Statistic p-value | Conf. level Conf. Set

------+---------------------------------+-------------------------------------

AR | chi2(1) = 37.89 0.0000 | 95% [ .281473, .54556]

------+---------------------------------+-------------------------------------

Wald | chi2(1) = 35.96 0.0000 | 95% [ .274737, .541518]

------------------------------------------------------------------------------

Confidence sets estimated for 100 points in [ .141346, .674908].

Number of obs N = 22781.

Method = minimum distance (MD).

Tests assume i.i.d. errors. Small sample adjustments were used.

Wald statistic in last row is based on ivtobit estimation and is not robust to weak instruments.

.

.

.

. ******Cragg-Donald test

.

. xi: ivreg2 PAT Size Age lev Top1 Mfee Growth ROE TobinQ Mshare Indep Dual SOE Board (DIF=distance), r

IV (2SLS) estimation

--------------------

Estimates efficient for homoskedasticity only

Statistics robust to heteroskedasticity

Number of obs = 22781

F( 14, 22766) = 21.92

Prob > F = 0.0000

Total (centered) SS = 53460008.7 Centered R2 = 0.0393

Total (uncentered) SS = 55036300 Uncentered R2 = 0.0668

Residual SS = 51358320.52 Root MSE = 47.48

------------------------------------------------------------------------------

| Robust

PAT | Coefficient std. err. z P>|z| [95% conf. interval]

-------------+----------------------------------------------------------------

DIF | .1704115 .0299198 5.70 0.000 .1117699 .2290532

Size | 5.990556 .6375275 9.40 0.000 4.741025 7.240087

Age | -26.74272 3.94539 -6.78 0.000 -34.47555 -19.0099

lev | 1.540327 1.84494 0.83 0.404 -2.075688 5.156343

Top1 | 13.18316 3.949239 3.34 0.001 5.442791 20.92352

Mfee | .0107083 .0019538 5.48 0.000 .006879 .0145376

Growth | .4424259 .2901024 1.53 0.127 -.1261643 1.011016

ROE | 5.264989 1.167833 4.51 0.000 2.97608 7.553899

TobinQ | -4.736128 .7461415 -6.35 0.000 -6.198539 -3.273718

Mshare | -32.29974 5.214961 -6.19 0.000 -42.52087 -22.0786

Indep | 43.15862 9.97194 4.33 0.000 23.61398 62.70327

Dual | 1.946186 .8613577 2.26 0.024 .2579555 3.634416

SOE | 4.151716 1.007088 4.12 0.000 2.177861 6.125571

Board | 5.941492 2.898715 2.05 0.040 .2601151 11.62287

_cons | -116.0386 13.14764 -8.83 0.000 -141.8075 -90.26968

------------------------------------------------------------------------------

Underidentification test (Kleibergen-Paap rk LM statistic): 356.657

Chi-sq(1) P-val = 0.0000

------------------------------------------------------------------------------

Weak identification test (Cragg-Donald Wald F statistic): 342.587

(Kleibergen-Paap rk Wald F statistic): 366.551

Stock-Yogo weak ID test critical values: 10% maximal IV size 16.38

15% maximal IV size 8.96

20% maximal IV size 6.66

25% maximal IV size 5.53

Source: Stock-Yogo (2005). Reproduced by permission.

NB: Critical values are for Cragg-Donald F statistic and i.i.d. errors.

------------------------------------------------------------------------------

Hansen J statistic (overidentification test of all instruments): 0.000

(equation exactly identified)

------------------------------------------------------------------------------

Instrumented: DIF

Included instruments: Size Age lev Top1 Mfee Growth ROE TobinQ Mshare Indep

Dual SOE Board

Excluded instruments: distance

------------------------------------------------------------------------------

.

**Table 6 Robustness test results.**

|  | (1) | (2) | (3) |
| --- | --- | --- | --- |
|  | PAT | INPAT | UPAT |
| main |  |  |  |
| DIF | 0.1513^**^ | 0.2130^***^ | 0.0483 |
|  | (2.5074) | (3.5005) | (1.5579) |
| Size | 16.3627^***^ | 13.3914^***^ | 7.4971^***^ |
|  | (5.0888) | (4.4441) | (5.2660) |
| Age | -28.1419^***^ | -19.4302^***^ | -14.2960^***^ |
|  | (-3.7145) | (-3.6408) | (-3.5624) |
| lev | -0.5322 | -6.9219 | 7.9207^**^ |
|  | (-0.0661) | (-1.0188) | (2.0972) |
| Top1 | 2.3529 | -12.4383 | 14.2299^*^ |
|  | (0.1434) | (-0.9146) | (1.8707) |
| Mfee | -12.4227^**^ | -5.3614^*^ | -8.3010^**^ |
|  | (-2.3722) | (-1.6519) | (-2.4690) |
| Growth | 0.3034 | 0.5343 | -0.4525 |
|  | (0.3032) | (0.6254) | (-0.8541) |
| ROE | 6.4640^**^ | 4.8269^*^ | 4.8321^**^ |
|  | (1.9682) | (1.7627) | (2.5739) |
| TobinQ | 2.6790 | 1.8250 | 0.8002 |
|  | (0.8815) | (0.9055) | (0.4052) |
| Mshare | 10.1503 | 17.2597 | -0.6150 |
|  | (0.5508) | (1.1795) | (-0.0540) |
| Indep | 27.9144 | 15.4250 | 19.2691 |
|  | (0.9903) | (0.7974) | (1.2200) |
| Dual | 4.5992^*^ | 4.4149^**^ | 1.0994 |
|  | (1.7798) | (2.1549) | (0.8770) |
| SOE | 1.3238 | 2.5494 | -0.8091 |
|  | (0.5100) | (1.1313) | (-0.6554) |
| Board | 0.8898 | 5.2296 | -2.8769 |
|  | (0.1120) | (0.7366) | (-0.8273) |
| sciedugdp | -0.0000^**^ | -0.0000^**^ | -0.0000^***^ |
|  | (-2.4800) | (-2.1184) | (-2.7623) |
| GDP2 | 0.0112 | 0.0437 | -0.0402 |
|  | (0.0683) | (0.3252) | (-0.4918) |
| Market | -0.4937 | -0.4691 | -0.1744 |
|  | (-0.7399) | (-0.8156) | (-0.5198) |
| FDI | 121.5999^**^ | 100.1263^**^ | 82.3251^**^ |
|  | (2.0098) | (2.0909) | (2.4245) |
| sub | 0.0000^***^ | 0.0000^***^ | 0.0000^***^ |
|  | (3.1981) | (2.8572) | (3.4886) |
| 2011.year | 0.0000 | 0.0000 | 0.0000 |
|  | (.) | (.) | (.) |
| 2012.year | 0.0061 | -2.7368 | 0.7227 |
|  | (0.0019) | (-0.9914) | (0.4424) |
| 2013.year | -2.9328 | -8.1316^*^ | -0.1998 |
|  | (-0.5351) | (-1.6670) | (-0.0702) |
| 2014.year | -0.2577 | -7.6307 | 1.7451 |
|  | (-0.0399) | (-1.3667) | (0.5099) |
| 2015.year | 1.1574 | -10.2527 | 3.7991 |
|  | (0.1399) | (-1.4301) | (0.8593) |
| 2016.year | 5.6808 | -8.7073 | 5.5835 |
|  | (0.5748) | (-1.0392) | (1.0782) |
| 2017.year | 8.1666 | -9.0759 | 7.2067 |
|  | (0.6947) | (-0.9151) | (1.1691) |
| 2018.year | 12.1817 | -7.9035 | 9.0365 |
|  | (0.9598) | (-0.7425) | (1.3528) |
| 2019.year | 14.5458 | -10.2658 | 12.5232^*^ |
|  | (1.0677) | (-0.8866) | (1.7235) |
| 2020.year | -4.2590 | -17.4789 | -5.5790 |
|  | (-0.3026) | (-1.4009) | (-0.7881) |
| 2021.year | -60.1101^***^ | -60.9623^***^ | -32.2828^***^ |
|  | (-3.5101) | (-3.7603) | (-3.4466) |
| 1.province | 0.0000 | 0.0000 | 0.0000 |
|  | (.) | (.) | (.) |
| 2.province | 3.5477 | -0.0389 | -0.2578 |
|  | (0.4397) | (-0.0061) | (-0.0691) |
| 3.province | -16.1679^**^ | -15.7468^**^ | -5.6388 |
|  | (-2.3549) | (-2.4889) | (-1.6444) |
| 4.province | -18.4909^*^ | -14.2806 | -3.7487 |
|  | (-1.8577) | (-1.6018) | (-0.7804) |
| 5.province | -6.7137 | -6.3917 | -2.9307 |
|  | (-1.1690) | (-1.3164) | (-0.9895) |
| 6.province | -32.3071^***^ | -19.9364^***^ | -17.4257^***^ |
|  | (-3.5856) | (-2.6077) | (-3.5850) |
| 7.province | -11.3926 | -3.9852 | -8.9301 |
|  | (-1.1684) | (-0.4947) | (-1.5762) |
| 8.province | -30.4583^***^ | -24.7905^**^ | -17.0221^***^ |
|  | (-2.7757) | (-2.5721) | (-3.0195) |
| 9.province | -15.8642^**^ | -10.4667 | -5.3428 |
|  | (-2.0490) | (-1.6217) | (-1.3734) |
| 10.province | -6.7998 | -7.4247 | -3.2230 |
|  | (-0.9144) | (-1.0249) | (-0.9751) |
| 11.province | -21.0102^**^ | -14.6431^*^ | -10.3889^*^ |
|  | (-2.0374) | (-1.7495) | (-1.8266) |
| 12.province | -8.5648 | -5.7742 | -2.2657 |
|  | (-1.3305) | (-1.0520) | (-0.6871) |
| 13.province | -15.1010^**^ | -10.3062^*^ | -8.3982^**^ |
|  | (-2.2058) | (-1.8009) | (-2.2365) |
| 14.province | -33.3019^***^ | -24.3607^***^ | -16.2794^***^ |
|  | (-3.4225) | (-2.9446) | (-3.2555) |
| 15.province | -9.2738 | -7.0687 | -4.3209 |
|  | (-1.6271) | (-1.4236) | (-1.5475) |
| 16.province | -16.0963^**^ | -16.3999^**^ | -5.6907 |
|  | (-2.1751) | (-2.3863) | (-1.5045) |
| 17.province | -25.9520^***^ | -19.7524^***^ | -11.7378^***^ |
|  | (-3.0379) | (-2.6408) | (-2.8112) |
| 18.province | -23.8441^***^ | -17.7117^**^ | -7.3422^*^ |
|  | (-2.8920) | (-2.5562) | (-1.7819) |
| 19.province | -33.4468^**^ | -23.2979^*^ | -20.5898^**^ |
|  | (-2.2514) | (-1.8735) | (-2.4430) |
| 20.province | -26.2829^***^ | -13.6481^*^ | -12.5750^**^ |
|  | (-2.7207) | (-1.7384) | (-2.0846) |
| 21.province | -2.4981 | -2.9250 | -0.9119 |
|  | (-0.4158) | (-0.5597) | (-0.3027) |
| 22.province | -27.3408^***^ | -25.6681^***^ | -10.6588^***^ |
|  | (-3.4221) | (-3.2384) | (-2.8218) |
| 23.province | -10.0740 | -13.8752^**^ | -3.9914 |
|  | (-1.4457) | (-2.2463) | (-1.0271) |
| 24.province | -26.0557^***^ | -22.5716^***^ | -12.0982^***^ |
|  | (-3.3635) | (-3.1510) | (-3.2317) |
| 25.province | -2.4957 | -0.6893 | -1.7223 |
|  | (-0.4035) | (-0.1344) | (-0.5328) |
| 26.province | -34.5551^***^ | -28.2600^***^ | -14.0055^***^ |
|  | (-3.2843) | (-3.0924) | (-2.7623) |
| 27.province | -53.3379^***^ | -36.8023^**^ | -31.7024^***^ |
|  | (-3.1988) | (-2.5045) | (-3.7539) |
| 28.province | -25.0921^*^ | -19.2314^*^ | -9.6479 |
|  | (-1.8058) | (-1.7576) | (-1.4528) |
| 29.province | -13.8595^*^ | -9.0744 | -10.5076^***^ |
|  | (-1.7666) | (-1.1823) | (-2.6979) |
| 30.province | -14.7297^***^ | -13.0526^***^ | -6.4299^**^ |
|  | (-2.7384) | (-2.7651) | (-2.2803) |
| 31.province | -19.0703^**^ | -14.3614^*^ | -9.6949^**^ |
|  | (-2.1676) | (-1.8904) | (-1.9793) |
| _cons | -3.5e+02^***^ | -3.2e+02^***^ | -1.6e+02^***^ |
|  | (-5.1736) | (-4.4597) | (-5.5868) |
| / |  |  |  |
| var(e.PAT) | 4.5e+03^***^ |  |  |
|  | (3.1304) |  |  |
| var(e.INPAT) |  | 2.8e+03^**^ |  |
|  |  | (2.5565) |  |
| var(e.UPAT) |  |  | 956.4178^***^ |
|  |  |  | (3.3358) |
| *N* | 22781 | 22781 | 22781 |
| pseudo *R*^2^ | 0.0354 | 0.0398 | 0.0476 |

*t* statistics in parentheses

^*^ *p* < 0.1, ^**^ *p* < 0.05, ^***^ *p* < 0.01

|  | (1) | (2) | (3) |
| --- | --- | --- | --- |
|  | PAT | INPAT | UPAT |
| DIF | 0.0778^***^ | 0.0653^***^ | 0.0126 |
|  | (2.7056) | (2.8491) | (1.2562) |
| Size | 1.4328^**^ | 0.8836^**^ | 0.5491^**^ |
|  | (2.2223) | (2.0739) | (1.9761) |
| Age | 14.6349 | 8.6521 | 5.9827 |
|  | (1.6423) | (1.5049) | (1.3615) |
| lev | 0.0414 | -0.6564 | 0.6978 |
|  | (0.0429) | (-0.9632) | (1.3250) |
| Top1 | 3.2342 | -0.6906 | 3.9248^*^ |
|  | (0.5190) | (-0.1441) | (1.7394) |
| Mfee | 0.0008 | 0.0005 | 0.0003 |
|  | (1.3498) | (1.4391) | (1.0131) |
| Growth | 0.2578 | 0.1682 | 0.0896 |
|  | (1.3151) | (1.4589) | (0.9454) |
| ROE | 0.2087 | -0.1167 | 0.3253 |
|  | (0.4461) | (-0.3813) | (1.5420) |
| TobinQ | 1.3811^***^ | 0.7587^***^ | 0.6224^***^ |
|  | (3.4079) | (3.0531) | (3.6118) |
| Mshare | 7.6129^**^ | 4.7280^*^ | 2.8850^**^ |
|  | (2.0349) | (1.7408) | (2.0847) |
| Indep | -11.2187 | -4.7445 | -6.4742 |
|  | (-1.0586) | (-0.7486) | (-1.1720) |
| Dual | 0.0881 | 0.3586 | -0.2705 |
|  | (0.1278) | (0.7008) | (-0.9618) |
| SOE | 1.7576^*^ | 0.8645 | 0.8931 |
|  | (1.9232) | (1.3085) | (1.3872) |
| Board | 1.3626 | 1.9047 | -0.5421 |
|  | (0.4703) | (0.8425) | (-0.4886) |
| 2011.year | 0.0000 | 0.0000 | 0.0000 |
|  | (.) | (.) | (.) |
| 2012.year | -4.0092^**^ | -3.3834^***^ | -0.6258 |
|  | (-2.5528) | (-2.7919) | (-0.9592) |
| 2013.year | -8.0687^***^ | -6.4229^***^ | -1.6459 |
|  | (-2.7331) | (-2.8475) | (-1.3432) |
| 2014.year | -8.7235^**^ | -6.9768^**^ | -1.7467 |
|  | (-2.4171) | (-2.5752) | (-1.1173) |
| 2015.year | -10.9589^**^ | -9.0404^**^ | -1.9185 |
|  | (-2.3413) | (-2.5167) | (-0.9991) |
| 2016.year | -10.7814^**^ | -9.0171^**^ | -1.7643 |
|  | (-1.9875) | (-2.1877) | (-0.7657) |
| 2017.year | -11.1150^*^ | -9.9393^**^ | -1.1757 |
|  | (-1.7453) | (-2.0416) | (-0.4399) |
| 2018.year | -10.8986 | -9.9250^*^ | -0.9735 |
|  | (-1.6001) | (-1.9201) | (-0.3378) |
| 2019.year | -11.5382 | -11.7726^**^ | 0.2345 |
|  | (-1.5782) | (-2.1194) | (0.0754) |
| 2020.year | -21.0871^***^ | -15.1825^**^ | -5.9047^*^ |
|  | (-2.6234) | (-2.5501) | (-1.6701) |
| 2021.year | -34.9018^***^ | -24.0559^***^ | -10.8459^**^ |
|  | (-3.4145) | (-3.2308) | (-2.5247) |
| 1.province | 0.0000 | 0.0000 | 0.0000 |
|  | (.) | (.) | (.) |
| 2.province | 0.0000 | 0.0000 | 0.0000 |
|  | (.) | (.) | (.) |
| 3.province | 0.0000 | 0.0000 | 0.0000 |
|  | (.) | (.) | (.) |
| 4.province | 0.0000 | 0.0000 | 0.0000 |
|  | (.) | (.) | (.) |
| 5.province | 0.0000 | 0.0000 | 0.0000 |
|  | (.) | (.) | (.) |
| 6.province | 0.0000 | 0.0000 | 0.0000 |
|  | (.) | (.) | (.) |
| 7.province | 0.0000 | 0.0000 | 0.0000 |
|  | (.) | (.) | (.) |
| 8.province | 0.0000 | 0.0000 | 0.0000 |
|  | (.) | (.) | (.) |
| 9.province | 0.0000 | 0.0000 | 0.0000 |
|  | (.) | (.) | (.) |
| 10.province | 0.0000 | 0.0000 | 0.0000 |
|  | (.) | (.) | (.) |
| 11.province | 0.0000 | 0.0000 | 0.0000 |
|  | (.) | (.) | (.) |
| 12.province | 0.0000 | 0.0000 | 0.0000 |
|  | (.) | (.) | (.) |
| 13.province | 0.0000 | 0.0000 | 0.0000 |
|  | (.) | (.) | (.) |
| 14.province | 0.0000 | 0.0000 | 0.0000 |
|  | (.) | (.) | (.) |
| 15.province | 0.0000 | 0.0000 | 0.0000 |
|  | (.) | (.) | (.) |
| 16.province | 0.0000 | 0.0000 | 0.0000 |
|  | (.) | (.) | (.) |
| 17.province | 0.0000 | 0.0000 | 0.0000 |
|  | (.) | (.) | (.) |
| 18.province | 0.0000 | 0.0000 | 0.0000 |
|  | (.) | (.) | (.) |
| 19.province | 0.0000 | 0.0000 | 0.0000 |
|  | (.) | (.) | (.) |
| 20.province | 0.0000 | 0.0000 | 0.0000 |
|  | (.) | (.) | (.) |
| 21.province | 0.0000 | 0.0000 | 0.0000 |
|  | (.) | (.) | (.) |
| 22.province | 0.0000 | 0.0000 | 0.0000 |
|  | (.) | (.) | (.) |
| 23.province | 0.0000 | 0.0000 | 0.0000 |
|  | (.) | (.) | (.) |
| 24.province | 0.0000 | 0.0000 | 0.0000 |
|  | (.) | (.) | (.) |
| 25.province | 0.0000 | 0.0000 | 0.0000 |
|  | (.) | (.) | (.) |
| 26.province | 0.0000 | 0.0000 | 0.0000 |
|  | (.) | (.) | (.) |
| 27.province | 0.0000 | 0.0000 | 0.0000 |
|  | (.) | (.) | (.) |
| 28.province | 0.0000 | 0.0000 | 0.0000 |
|  | (.) | (.) | (.) |
| 29.province | 0.0000 | 0.0000 | 0.0000 |
|  | (.) | (.) | (.) |
| 30.province | 0.0000 | 0.0000 | 0.0000 |
|  | (.) | (.) | (.) |
| 31.province | 0.0000 | 0.0000 | 0.0000 |
|  | (.) | (.) | (.) |
| _cons | -71.0917^***^ | -46.9828^***^ | -24.1089^***^ |
|  | (-3.4305) | (-3.1981) | (-2.6196) |
| *N* | 22781 | 22781 | 22781 |
| pseudo *R*^2^ |  |  |  |

*t* statistics in parentheses

^*^ *p* < 0.1, ^**^ *p* < 0.05, ^***^ *p* < 0.01

|  | (1) | (2) | (3) |
| --- | --- | --- | --- |
|  | PAT | INPAT | UPAT |
| main |  |  |  |
| DIF | 0.1559^**^ | 0.2134^***^ | 0.0612^**^ |
|  | (2.4811) | (3.3093) | (2.0141) |
| Size | 19.2558^***^ | 15.7184^***^ | 8.4285^***^ |
|  | (4.9147) | (4.2953) | (5.2220) |
| Age | -29.7629^***^ | -20.9060^***^ | -14.7972^***^ |
|  | (-3.7072) | (-3.6424) | (-3.5751) |
| lev | -2.2794 | -8.6563 | 7.4485^*^ |
|  | (-0.2593) | (-1.1520) | (1.8874) |
| Top1 | 7.1827 | -8.6584 | 15.9459^**^ |
|  | (0.4261) | (-0.6366) | (2.0319) |
| Mfee | -13.1566^**^ | -5.8094 | -8.8542^**^ |
|  | (-2.2439) | (-1.6125) | (-2.3611) |
| Growth | 0.5183 | 0.8437 | -0.4365 |
|  | (0.4739) | (0.8889) | (-0.7806) |
| ROE | 5.1601 | 3.5602 | 4.5430^**^ |
|  | (1.4439) | (1.1671) | (2.3027) |
| TobinQ | 3.4598 | 2.4322 | 1.2058 |
|  | (1.1118) | (1.1567) | (0.6390) |
| Mshare | -0.2066 | 8.3645 | -3.3885 |
|  | (-0.0113) | (0.6020) | (-0.2952) |
| Indep | 44.6418 | 29.5349 | 24.1656 |
|  | (1.4190) | (1.3233) | (1.4302) |
| Dual | 4.9278^*^ | 4.6799^**^ | 0.9855 |
|  | (1.7289) | (2.0646) | (0.7316) |
| SOE | 1.7006 | 2.9100 | -0.8286 |
|  | (0.6109) | (1.2021) | (-0.6451) |
| Board | 4.0719 | 8.2636 | -2.1104 |
|  | (0.4477) | (0.9997) | (-0.5676) |
| 2011.year | 0.0000 | 0.0000 | 0.0000 |
|  | (.) | (.) | (.) |
| 2012.year | -1.3880 | -3.7528 | -0.2083 |
|  | (-0.4308) | (-1.2840) | (-0.1301) |
| 2013.year | -5.5303 | -10.0414^*^ | -1.9876 |
|  | (-0.9829) | (-1.9158) | (-0.7190) |
| 2014.year | -1.3846 | -8.3981 | 0.2596 |
|  | (-0.2085) | (-1.4207) | (0.0786) |
| 2016.year | 0.8671 | -12.5063 | 2.4389 |
|  | (0.0865) | (-1.4072) | (0.4886) |
| 2017.year | 3.3743 | -12.7200 | 3.7586 |
|  | (0.2820) | (-1.2104) | (0.6323) |
| 2018.year | 6.5428 | -12.2772 | 4.9859 |
|  | (0.5096) | (-1.0925) | (0.7790) |
| 2019.year | 8.8031 | -14.9296 | 8.3589 |
|  | (0.6356) | (-1.2206) | (1.1971) |
| 2020.year | -10.1293 | -22.0300^*^ | -10.1265 |
|  | (-0.7034) | (-1.6742) | (-1.4519) |
| 2021.year | -76.4636^***^ | -73.1983^***^ | -42.9292^***^ |
|  | (-3.9439) | (-3.9193) | (-4.1255) |
| 1.province | 0.0000 | 0.0000 | 0.0000 |
|  | (.) | (.) | (.) |
| 2.province | 4.4813 | 0.1490 | 0.6662 |
|  | (0.5118) | (0.0226) | (0.1744) |
| 3.province | -20.0380^***^ | -18.9128^***^ | -7.4609^**^ |
|  | (-2.7683) | (-2.8364) | (-2.0962) |
| 4.province | -22.9167^**^ | -17.8313^**^ | -5.4776 |
|  | (-2.2856) | (-2.0001) | (-1.1592) |
| 5.province | -9.9539^*^ | -8.9414^*^ | -4.3433 |
|  | (-1.6629) | (-1.7787) | (-1.4319) |
| 6.province | -38.2915^***^ | -24.9467^***^ | -20.0042^***^ |
|  | (-3.9203) | (-3.0468) | (-3.8776) |
| 7.province | -20.0085^*^ | -12.0274 | -12.3181^**^ |
|  | (-1.9077) | (-1.3703) | (-2.0278) |
| 8.province | -34.8262^***^ | -28.4103^***^ | -17.3419^***^ |
|  | (-3.2348) | (-3.0631) | (-3.1756) |
| 9.province | -19.3680^**^ | -13.7693^**^ | -6.3894 |
|  | (-2.3241) | (-1.9818) | (-1.5710) |
| 10.province | -9.5188 | -10.1152 | -3.8736 |
|  | (-1.2167) | (-1.3138) | (-1.1199) |
| 11.province | -25.7580^**^ | -18.7705^**^ | -10.8417^*^ |
|  | (-2.3769) | (-2.1549) | (-1.9101) |
| 12.province | -12.5280^*^ | -9.0972 | -3.8829 |
|  | (-1.8199) | (-1.5575) | (-1.1266) |
| 13.province | -16.5565^**^ | -11.0538^*^ | -8.9220^**^ |
|  | (-2.2999) | (-1.8602) | (-2.2780) |
| 14.province | -37.6904^***^ | -27.3315^***^ | -17.8880^***^ |
|  | (-3.5793) | (-3.1619) | (-3.3587) |
| 15.province | -13.1111^**^ | -10.4846^*^ | -5.8147^**^ |
|  | (-2.1351) | (-1.9558) | (-1.9778) |
| 16.province | -17.3276^**^ | -17.2164^**^ | -5.7122 |
|  | (-2.2237) | (-2.3836) | (-1.4471) |
| 17.province | -28.2370^***^ | -22.3563^***^ | -11.5645^***^ |
|  | (-3.0820) | (-2.7519) | (-2.6706) |
| 18.province | -27.3379^***^ | -21.5871^***^ | -8.2405^**^ |
|  | (-3.2566) | (-3.1303) | (-2.0078) |
| 19.province | -35.4317^**^ | -25.2877^*^ | -22.2810^***^ |
|  | (-2.2844) | (-1.9293) | (-2.5928) |
| 20.province | -35.1895^***^ | -21.6725^**^ | -16.4167^**^ |
|  | (-3.2586) | (-2.4026) | (-2.4252) |
| 21.province | -5.6002 | -5.5259 | -1.9681 |
|  | (-0.8862) | (-1.0076) | (-0.6309) |
| 22.province | -34.9819^***^ | -32.8669^***^ | -13.4382^***^ |
|  | (-3.9700) | (-3.6371) | (-3.3651) |
| 23.province | -13.1296^*^ | -16.5108^**^ | -4.9375 |
|  | (-1.8064) | (-2.5456) | (-1.2270) |
| 24.province | -26.5638^***^ | -23.2191^***^ | -11.8173^***^ |
|  | (-3.5143) | (-3.3643) | (-3.2093) |
| 25.province | -6.2450 | -4.3819 | -3.0514 |
|  | (-0.9638) | (-0.8162) | (-0.9105) |
| 26.province | -31.5704^***^ | -26.8624^***^ | -10.9167^**^ |
|  | (-3.2928) | (-3.1878) | (-2.3094) |
| 27.province | -59.1893^***^ | -39.7194^**^ | -35.7471^***^ |
|  | (-3.5560) | (-2.5746) | (-4.1384) |
| 28.province | -30.8685^**^ | -23.8459^**^ | -11.8926^*^ |
|  | (-2.1724) | (-2.1368) | (-1.7898) |
| 29.province | -18.2517^**^ | -13.0655 | -12.2294^***^ |
|  | (-2.2015) | (-1.6034) | (-3.0852) |
| 30.province | -17.8909^***^ | -15.7436^***^ | -7.8987^***^ |
|  | (-3.1511) | (-3.1711) | (-2.6586) |
| 31.province | -23.2824^**^ | -18.5035^**^ | -11.8398^**^ |
|  | (-2.5081) | (-2.2831) | (-2.3426) |
| _cons | -4.2e+02^***^ | -3.7e+02^***^ | -1.8e+02^***^ |
|  | (-5.0329) | (-4.2974) | (-5.7859) |
| / |  |  |  |
| var(e.PAT) | 4.9e+03^***^ |  |  |
|  | (3.0916) |  |  |
| var(e.INPAT) |  | 3.0e+03^**^ |  |
|  |  | (2.5757) |  |
| var(e.UPAT) |  |  | 1.0e+03^***^ |
|  |  |  | (3.2729) |
| *N* | 20710 | 20710 | 20710 |
| pseudo *R*^2^ | 0.0315 | 0.0353 | 0.0463 |

*t* statistics in parentheses

^*^ *p* < 0.1, ^**^ *p* < 0.05, ^***^ *p* < 0.01

|  | (1) | (2) | (3) |
| --- | --- | --- | --- |
|  | PAT | INPAT | UPAT |
| main |  |  |  |
| DIF | 0.1306^**^ | 0.1592^***^ | 0.0469^**^ |
|  | (2.5627) | (3.1375) | (2.0733) |
| Size | 14.1518^***^ | 11.6273^***^ | 6.1367^***^ |
|  | (4.4015) | (4.5718) | (4.4312) |
| Age | -9.8227^***^ | -8.1234^***^ | -4.1287^***^ |
|  | (-3.2473) | (-2.8824) | (-3.1258) |
| lev | -7.2493 | -8.1283 | 0.2829 |
|  | (-0.9266) | (-1.3418) | (0.0906) |
| Top1 | -19.0197^**^ | -17.5433^**^ | -4.4881 |
|  | (-2.1242) | (-2.3405) | (-1.2491) |
| Mfee | -4.7159^*^ | -2.1795^*^ | -2.8441^*^ |
|  | (-1.7303) | (-1.8717) | (-1.7532) |
| Growth | 0.2042 | 0.3708 | -0.2256 |
|  | (0.2304) | (0.4987) | (-0.5070) |
| ROE | 4.1522^*^ | 3.0257 | 2.8799^**^ |
|  | (1.6647) | (1.3943) | (2.0872) |
| TobinQ | 1.3353 | 0.9335 | 0.4205 |
|  | (1.4870) | (1.3518) | (0.6588) |
| Mshare | 8.5545 | 7.3629 | 5.7315 |
|  | (0.6343) | (0.7509) | (0.7021) |
| Indep | -19.7332 | -10.5656 | -8.2271 |
|  | (-0.9568) | (-0.6801) | (-0.7957) |
| Dual | 6.4394^**^ | 5.3928^**^ | 2.1867^*^ |
|  | (2.1588) | (2.3919) | (1.6756) |
| SOE | 0.0985 | 1.3420 | -1.2698 |
|  | (0.0388) | (0.6279) | (-1.1813) |
| Board | -4.4122 | -0.8644 | -3.5045 |
|  | (-0.6243) | (-0.1472) | (-1.1632) |
| 2011.year | 0.0000 | 0.0000 | 0.0000 |
|  | (.) | (.) | (.) |
| 2012.year | -2.5830 | -3.2424 | -0.8465 |
|  | (-1.0876) | (-1.4607) | (-0.7623) |
| 2013.year | -6.9133 | -8.5206^**^ | -2.5290 |
|  | (-1.6261) | (-2.1059) | (-1.2900) |
| 2014.year | -5.3859 | -8.2504^*^ | -1.7024 |
|  | (-1.0987) | (-1.7977) | (-0.7294) |
| 2015.year | -7.1214 | -11.8463^**^ | -1.6672 |
|  | (-1.1157) | (-1.9684) | (-0.5517) |
| 2016.year | -6.9486 | -12.4776^*^ | -1.7628 |
|  | (-0.9408) | (-1.7741) | (-0.5197) |
| 2017.year | -6.4568 | -13.5131 | -1.3333 |
|  | (-0.7373) | (-1.6286) | (-0.3298) |
| 2018.year | -3.9937 | -13.0534 | -0.1532 |
|  | (-0.4340) | (-1.5002) | (-0.0354) |
| 2019.year | -3.2840 | -15.2103 | 1.7009 |
|  | (-0.3322) | (-1.6121) | (0.3695) |
| 2020.year | -17.4327 | -20.9708^**^ | -10.8363^**^ |
|  | (-1.5983) | (-2.0474) | (-2.0547) |
| 2021.year | -53.6896^***^ | -50.6802^***^ | -27.4098^***^ |
|  | (-3.7874) | (-3.7931) | (-3.9936) |
| 1.province | 0.0000 | 0.0000 | 0.0000 |
|  | (.) | (.) | (.) |
| 3.province | -16.5863^***^ | -14.7104^***^ | -6.4853^***^ |
|  | (-3.0662) | (-3.0850) | (-2.5976) |
| 4.province | -17.3107^**^ | -13.6701^**^ | -4.4853 |
|  | (-2.4177) | (-2.2015) | (-1.3421) |
| 5.province | -6.5964 | -6.0136^*^ | -2.8700 |
|  | (-1.5054) | (-1.6679) | (-1.3802) |
| 6.province | -26.6001^***^ | -17.9398^***^ | -12.9550^***^ |
|  | (-3.9599) | (-3.2652) | (-3.8743) |
| 7.province | -13.6671^*^ | -7.8200 | -8.0171^**^ |
|  | (-1.8858) | (-1.3225) | (-1.9684) |
| 8.province | -25.2372^***^ | -20.7828^***^ | -12.0627^***^ |
|  | (-3.5282) | (-3.3304) | (-3.6013) |
| 9.province | -12.1507^**^ | -9.1333^*^ | -3.7555 |
|  | (-2.0894) | (-1.9106) | (-1.3654) |
| 10.province | -6.3721 | -5.9339 | -3.1302 |
|  | (-1.0167) | (-0.9923) | (-1.3103) |
| 11.province | -17.9708^**^ | -13.3945^**^ | -7.5120^**^ |
|  | (-2.5657) | (-2.2894) | (-2.1354) |
| 12.province | -11.2802^**^ | -7.7972^*^ | -4.2266^*^ |
|  | (-2.2151) | (-1.8758) | (-1.7452) |
| 13.province | -12.1356^**^ | -8.3579^**^ | -6.0952^**^ |
|  | (-2.4378) | (-2.0395) | (-2.3885) |
| 14.province | -27.5152^***^ | -20.5424^***^ | -12.6105^***^ |
|  | (-3.7245) | (-3.4667) | (-3.5951) |
| 15.province | -10.3320^**^ | -7.5675^*^ | -4.8210^**^ |
|  | (-2.1800) | (-1.9281) | (-2.3015) |
| 16.province | -14.0196^**^ | -13.4655^***^ | -4.8050^*^ |
|  | (-2.5169) | (-2.7456) | (-1.7364) |
| 17.province | -19.7722^***^ | -15.3064^***^ | -8.3368^***^ |
|  | (-3.0827) | (-2.8352) | (-2.8911) |
| 18.province | -20.4249^***^ | -15.6617^***^ | -6.8663^**^ |
|  | (-3.2713) | (-3.1803) | (-2.2425) |
| 19.province | -26.9529^***^ | -18.9196^**^ | -15.6119^***^ |
|  | (-2.6889) | (-2.2383) | (-2.8499) |
| 20.province | -22.2446^***^ | -13.5962^**^ | -9.7301^**^ |
|  | (-3.2914) | (-2.4707) | (-2.4454) |
| 21.province | -4.8926 | -4.1411 | -2.2836 |
|  | (-0.9982) | (-1.0184) | (-1.0022) |
| 22.province | -23.5274^***^ | -22.1977^***^ | -8.5602^***^ |
|  | (-3.9746) | (-3.8859) | (-3.3094) |
| 23.province | -8.2896 | -11.0748^**^ | -2.6601 |
|  | (-1.5796) | (-2.4708) | (-0.9724) |
| 25.province | -6.3469 | -3.8011 | -3.3840 |
|  | (-1.3190) | (-0.9687) | (-1.4468) |
| 27.province | -42.0899^***^ | -29.7170^***^ | -23.5821^***^ |
|  | (-4.0030) | (-3.2470) | (-4.5517) |
| 28.province | -18.3644 | -15.1659^*^ | -6.6429 |
|  | (-1.5659) | (-1.7568) | (-1.2359) |
| 29.province | -15.2888^***^ | -10.7947^**^ | -9.4367^***^ |
|  | (-2.6730) | (-1.9788) | (-3.4067) |
| 30.province | -15.1140^***^ | -12.5907^***^ | -6.4315^***^ |
|  | (-3.4593) | (-3.4755) | (-3.0039) |
| _cons | -2.9e+02^***^ | -2.6e+02^***^ | -1.3e+02^***^ |
|  | (-4.6263) | (-4.7072) | (-4.6944) |
| / |  |  |  |
| var(e.PAT) | 2.0e+03^***^ |  |  |
|  | (2.8012) |  |  |
| var(e.INPAT) |  | 1.3e+03^***^ |  |
|  |  | (2.9337) |  |
| var(e.UPAT) |  |  | 374.9175^***^ |
|  |  |  | (2.9684) |
| *N* | 18271 | 18271 | 18271 |
| pseudo *R*^2^ | 0.0306 | 0.0351 | 0.0440 |

*t* statistics in parentheses

^*^ *p* < 0.1, ^**^ *p* < 0.05, ^***^ *p* < 0.01

|  | (1) | (2) | (3) | (4) |
| --- | --- | --- | --- | --- |
|  | GPR | GPR | GPR | GPR |
| GPR |  |  |  |  |
| DIF | 1.5224^**^ |  |  |  |
|  | (2.0931) |  |  |  |
| Size | 139.7481^***^ | 139.8216^***^ | 139.8864^***^ | 140.0588^***^ |
|  | (2.9093) | (2.9103) | (2.9097) | (2.9112) |
| Age | -1.1e+02^**^ | -1.1e+02^**^ | -1.2e+02^**^ | -1.2e+02^**^ |
|  | (-2.3727) | (-2.3688) | (-2.3827) | (-2.3922) |
| lev | -19.6325 | -20.3767 | -15.9010 | -13.7373 |
|  | (-0.2945) | (-0.3049) | (-0.2409) | (-0.2093) |
| Top1 | -1.7e+02 | -1.7e+02 | -1.7e+02 | -1.7e+02 |
|  | (-0.9237) | (-0.9307) | (-0.9051) | (-0.8997) |
| Mfee | -16.0702 | -16.1141 | -15.8559 | -15.8343 |
|  | (-1.0792) | (-1.0794) | (-1.0647) | (-1.0593) |
| Growth | -3.2270 | -3.4833 | -2.6589 | -2.3086 |
|  | (-0.2722) | (-0.2926) | (-0.2248) | (-0.1948) |
| ROE | -7.3382 | -8.4202 | -7.2107 | -8.0766 |
|  | (-0.1871) | (-0.2139) | (-0.1831) | (-0.2042) |
| TobinQ | 0.2425 | 0.2887 | -0.0908 | -0.2726 |
|  | (0.0150) | (0.0179) | (-0.0056) | (-0.0168) |
| Mshare | -65.0912 | -60.3226 | -66.6731 | -64.1110 |
|  | (-0.4606) | (-0.4257) | (-0.4734) | (-0.4564) |
| Indep | 281.6543 | 281.3484 | 281.3310 | 277.9628 |
|  | (1.4648) | (1.4638) | (1.4616) | (1.4453) |
| Dual | 35.7475^*^ | 35.6478^*^ | 36.3008^*^ | 36.2602^*^ |
|  | (1.7052) | (1.7027) | (1.7269) | (1.7265) |
| SOE | 35.4454 | 35.8088 | 35.2100 | 34.9742 |
|  | (1.0905) | (1.0996) | (1.0848) | (1.0785) |
| Board | 117.4827 | 117.5129 | 117.1725 | 115.2168 |
|  | (1.4081) | (1.4081) | (1.4050) | (1.3851) |
| 2011.year | 0.0000 | 0.0000 | 0.0000 | 0.0000 |
|  | (.) | (.) | (.) | (.) |
| 2012.year | 55.9913 | 76.2149^**^ | 74.5460^*^ | 109.9458^***^ |
|  | (1.4356) | (2.1177) | (1.8058) | (2.6144) |
| 2013.year | 81.6303 | 130.1967^**^ | 111.8975 | 175.7413^**^ |
|  | (1.2106) | (2.1836) | (1.5531) | (2.4235) |
| 2014.year | 127.6811 | 158.4030^**^ | 189.7053^**^ | 244.5363^***^ |
|  | (1.5297) | (2.0319) | (2.2307) | (2.7072) |
| 2015.year | 136.8260 | 189.6280^**^ | 219.9456^**^ | 275.6564^**^ |
|  | (1.3512) | (2.0746) | (2.1837) | (2.5092) |
| 2016.year | 154.7421 | 224.8116^**^ | 220.6159^*^ | 322.9194^***^ |
|  | (1.3463) | (2.2058) | (1.8479) | (2.6607) |
| 2017.year | 156.1917 | 249.6913^**^ | 205.4715 | 363.8364^***^ |
|  | (1.1888) | (2.2266) | (1.4318) | (2.7396) |
| 2018.year | 183.1660 | 276.3369^**^ | 261.0200^*^ | 401.0036^***^ |
|  | (1.2933) | (2.2672) | (1.7702) | (2.7636) |
| 2019.year | 203.8866 | 297.5549^**^ | 293.0051^*^ | 441.3136^***^ |
|  | (1.3457) | (2.2758) | (1.8777) | (2.8709) |
| 2020.year | 203.1737 | 297.5071^**^ | 298.3600^*^ | 456.9458^***^ |
|  | (1.2933) | (2.2104) | (1.8585) | (2.9316) |
| 2021.year | -2.9e+02^*^ | -2.0e+02 | -1.7e+02 | 3.6579 |
|  | (-1.7429) | (-1.5781) | (-1.1867) | (0.0362) |
| 1.province | 0.0000 | 0.0000 | 0.0000 | 0.0000 |
|  | (.) | (.) | (.) | (.) |
| 2.province | 100.4548 | 95.7070 | 122.9527 | 145.1187 |
|  | (1.0883) | (1.0449) | (1.2905) | (1.4783) |
| 3.province | -1.4e+02^*^ | -1.5e+02^*^ | -1.3e+02^*^ | -1.2e+02 |
|  | (-1.8254) | (-1.8630) | (-1.6667) | (-1.5673) |
| 4.province | -2.9e+02^*^ | -3.1e+02^*^ | -2.6e+02^*^ | -3.1e+02^*^ |
|  | (-1.8228) | (-1.9134) | (-1.6511) | (-1.9227) |
| 5.province | -2.8011 | -11.4130 | 21.6883 | 31.9744 |
|  | (-0.0532) | (-0.2125) | (0.4264) | (0.6343) |
| 6.province | -2.0e+02^*^ | -2.1e+02^*^ | -1.9e+02 | -2.2e+02^*^ |
|  | (-1.6490) | (-1.7030) | (-1.5817) | (-1.7762) |
| 7.province | -25.0873 | -42.3061 | 0.1317 | -40.4663 |
|  | (-0.2751) | (-0.4626) | (0.0014) | (-0.4388) |
| 8.province | -2.3e+02^*^ | -2.5e+02^*^ | -2.0e+02 | -2.2e+02 |
|  | (-1.6590) | (-1.7465) | (-1.4595) | (-1.5779) |
| 9.province | -83.3632 | -92.6049 | -80.6492 | -1.1e+02 |
|  | (-1.1108) | (-1.2304) | (-1.0600) | (-1.4725) |
| 10.province | 42.2807 | 30.2710 | 60.8543 | 29.8859 |
|  | (0.5372) | (0.3823) | (0.7675) | (0.3789) |
| 11.province | -78.8781 | -88.7756 | -66.9239 | -1.0e+02 |
|  | (-0.7383) | (-0.8300) | (-0.6199) | (-0.9270) |
| 12.province | -33.6836 | -38.4449 | -21.5484 | -25.3610 |
|  | (-0.5158) | (-0.5836) | (-0.3338) | (-0.3912) |
| 13.province | -49.8890 | -54.6190 | -44.4327 | -53.1075 |
|  | (-0.7685) | (-0.8353) | (-0.6872) | (-0.8119) |
| 14.province | -1.9e+02 | -2.0e+02^*^ | -1.8e+02 | -2.1e+02^*^ |
|  | (-1.6195) | (-1.7143) | (-1.5264) | (-1.7963) |
| 15.province | -39.5635 | -44.0934 | -24.3676 | -12.3391 |
|  | (-0.7029) | (-0.7703) | (-0.4434) | (-0.2237) |
| 16.province | -1.4e+02 | -1.4e+02 | -1.4e+02 | -1.5e+02 |
|  | (-1.5534) | (-1.5673) | (-1.5375) | (-1.6106) |
| 17.province | -71.8046 | -81.2346 | -61.1784 | -80.4141 |
|  | (-0.8392) | (-0.9399) | (-0.7178) | (-0.9309) |
| 18.province | -1.7e+02^*^ | -1.9e+02^**^ | -1.5e+02 | -2.0e+02^**^ |
|  | (-1.9543) | (-2.1003) | (-1.6337) | (-2.1359) |
| 19.province | -26.9712 | -50.8845 | -0.8926 | -52.8846 |
|  | (-0.2722) | (-0.5139) | (-0.0088) | (-0.5461) |
| 20.province | -1.4e+02 | -1.6e+02 | -1.2e+02 | -1.8e+02^*^ |
|  | (-1.4272) | (-1.6143) | (-1.2023) | (-1.7230) |
| 21.province | 16.4549 | 9.1336 | 26.1875 | 10.2853 |
|  | (0.3252) | (0.1802) | (0.5105) | (0.2013) |
| 22.province | -2.2e+02^**^ | -2.4e+02^**^ | -2.0e+02^**^ | -2.4e+02^**^ |
|  | (-2.2188) | (-2.3337) | (-2.0059) | (-2.3552) |
| 23.province | -1.3e+02 | -1.4e+02 | -1.1e+02 | -1.3e+02 |
|  | (-1.4684) | (-1.5887) | (-1.2185) | (-1.4882) |
| 24.province | -1.3e+02^*^ | -1.2e+02^*^ | -1.2e+02 | -75.3618 |
|  | (-1.7107) | (-1.6608) | (-1.6365) | (-1.2096) |
| 25.province | 57.3582 | 51.0411 | 63.8731 | 46.9084 |
|  | (0.9762) | (0.8747) | (1.0713) | (0.8004) |
| 26.province | -1.8e+02^*^ | -1.9e+02^*^ | -1.7e+02^*^ | -1.8e+02^*^ |
|  | (-1.8753) | (-1.9091) | (-1.8086) | (-1.8574) |
| 27.province | -2.1e+02 | -2.2e+02 | -1.8e+02 | -2.2e+02 |
|  | (-1.1520) | (-1.2187) | (-1.0149) | (-1.2084) |
| 28.province | -2.3e+02^*^ | -2.5e+02^**^ | -2.0e+02^*^ | -2.4e+02^*^ |
|  | (-1.8847) | (-1.9988) | (-1.6700) | (-1.9378) |
| 29.province | -49.0211 | -60.1835 | -33.8632 | -59.2587 |
|  | (-0.6113) | (-0.7411) | (-0.4272) | (-0.7506) |
| 30.province | -1.0e+02^*^ | -1.0e+02^*^ | -98.7307^*^ | -65.0314 |
|  | (-1.8686) | (-1.8328) | (-1.8031) | (-1.3036) |
| 31.province | -87.7380 | -95.0783 | -81.0715 | -1.0e+02 |
|  | (-0.8828) | (-0.9516) | (-0.8147) | (-1.0069) |
| DIFB |  | 1.1562^**^ |  |  |
|  |  | (2.0790) |  |  |
| DIFD |  |  | 1.1198^*^ |  |
|  |  |  | (1.7807) |  |
| DIFL |  |  |  | 0.3041 |
|  |  |  |  | (1.1499) |
| _cons | -3.8e+03^***^ | -3.8e+03^***^ | -3.8e+03^***^ | -3.8e+03^***^ |
|  | (-2.9601) | (-2.9540) | (-2.9613) | (-2.9437) |
| / |  |  |  |  |
| var(e.GPR) | 4.3e+05 | 4.3e+05 | 4.3e+05 | 4.3e+05 |
|  | (1.5300) | (1.5300) | (1.5301) | (1.5303) |
| *N* | 22781 | 22781 | 22781 | 22781 |
| pseudo *R*^2^ | 0.0169 | 0.0169 | 0.0168 | 0.0168 |

*t* statistics in parentheses

^*^ *p* < 0.1, ^**^ *p* < 0.05, ^***^ *p* < 0.01

**Table 7 Sample results of enterprise size.**

|  | (1) | (2) | (3) | (4) | (5) | (6) |
| --- | --- | --- | --- | --- | --- | --- |
|  | PAT | INPAT | UPAT | PAT | INPAT | UPAT |
| main |  |  |  |  |  |  |
| DIF | 0.0900^***^ | 0.0993^***^ | 0.0450^***^ | 0.0774 | 0.1074 | 0.0005 |
|  | (3.9953) | (5.2167) | (2.6851) | (0.6532) | (1.0253) | (0.0094) |
| Size | 5.0211^***^ | 4.0359^***^ | 2.9856^***^ | 22.8359^***^ | 17.4735^***^ | 9.2383^***^ |
|  | (6.9853) | (7.1664) | (4.9667) | (3.5280) | (3.1988) | (3.6281) |
| Age | -4.1815^***^ | -3.1552^***^ | -2.7942^***^ | -39.3230^***^ | -26.0445^***^ | -18.1873^***^ |
|  | (-3.1394) | (-2.9601) | (-2.9126) | (-3.2610) | (-3.0467) | (-3.1027) |
| lev | 0.4122 | -1.5080 | 2.1526^**^ | -6.1613 | -10.9188 | 10.0086 |
|  | (0.2666) | (-1.0895) | (2.0676) | (-0.3680) | (-0.8298) | (1.4315) |
| Top1 | -10.1395^**^ | -9.2213^**^ | -5.0874^*^ | 15.3134 | -4.0871 | 24.0205^**^ |
|  | (-2.1498) | (-2.0865) | (-1.7684) | (0.6453) | (-0.2181) | (2.3193) |
| Mfee | -1.5810^*^ | -0.4034 | -2.1578^**^ | -13.4107 | -5.6443 | -9.1673 |
|  | (-1.7236) | (-0.7642) | (-2.5253) | (-1.0353) | (-1.0902) | (-1.2520) |
| Growth | 0.1051 | 0.1132 | -0.0356 | 1.3850 | 1.3630 | -0.4755 |
|  | (0.3103) | (0.4444) | (-0.1454) | (0.5034) | (0.5947) | (-0.3882) |
| ROE | 1.1072 | 0.4531 | 1.3235^*^ | 11.4301 | 11.6706 | 5.2759 |
|  | (1.2668) | (0.6180) | (1.8845) | (1.2182) | (1.5678) | (1.1627) |
| TobinQ | 0.1061 | 0.0250 | -0.0616 | 4.0579 | 2.9463 | 0.8404 |
|  | (0.3025) | (0.0899) | (-0.1640) | (0.6122) | (0.6902) | (0.2269) |
| Mshare | -5.2098 | -1.8872 | -4.1640 | 17.8136 | 22.2840 | 2.6955 |
|  | (-1.1137) | (-0.4990) | (-1.0805) | (0.4697) | (0.8061) | (0.1249) |
| Indep | -1.7168 | -3.7284 | 1.4026 | 89.8087^*^ | 58.3434^*^ | 44.6776^*^ |
|  | (-0.1944) | (-0.7024) | (0.1782) | (1.7555) | (1.6580) | (1.6668) |
| Dual | 0.2117 | 0.4597 | -0.2282 | 9.4362 | 8.7767^**^ | 2.9466 |
|  | (0.3255) | (0.8602) | (-0.4625) | (1.5998) | (1.9698) | (1.1065) |
| SOE | -0.4949 | 0.3361 | -1.0080^*^ | 4.3528 | 4.4613 | 0.4191 |
|  | (-0.5585) | (0.4295) | (-1.6650) | (0.8363) | (1.0569) | (0.1780) |
| Board | 0.8471 | 0.0629 | 1.3103 | 7.8584 | 14.3666 | -4.6861 |
|  | (0.3503) | (0.0370) | (0.6728) | (0.5059) | (1.0649) | (-0.7636) |
| 2011.year | 0.0000 | 0.0000 | 0.0000 | 0.0000 | 0.0000 | 0.0000 |
|  | (.) | (.) | (.) | (.) | (.) | (.) |
| 2012.year | -2.5220^**^ | -2.8650^***^ | -1.1552 | 2.5683 | 0.5237 | 2.9595 |
|  | (-2.2167) | (-3.0717) | (-1.2833) | (0.4280) | (0.1007) | (1.0288) |
| 2013.year | -6.1239^***^ | -7.0205^***^ | -2.9700^*^ | 3.7875 | 2.4347 | 4.1312 |
|  | (-3.0273) | (-4.1358) | (-1.9069) | (0.3555) | (0.2632) | (0.8240) |
| 2014.year | -5.5446^**^ | -7.0214^***^ | -2.4557 | 9.9229 | 6.0657 | 7.6247 |
|  | (-2.2811) | (-3.7032) | (-1.2286) | (0.7889) | (0.5677) | (1.2661) |
| 2015.year | -7.2026^**^ | -9.6670^***^ | -2.4967 | 11.6142 | 5.1717 | 10.3269 |
|  | (-2.2769) | (-3.9530) | (-0.9355) | (0.7207) | (0.3767) | (1.3409) |
| 2016.year | -7.9622^**^ | -10.7778^***^ | -3.1486 | 19.5676 | 10.5801 | 14.2213 |
|  | (-2.3310) | (-3.8614) | (-1.1399) | (1.0278) | (0.6582) | (1.5602) |
| 2017.year | -9.1145^**^ | -12.2707^***^ | -3.9595 | 27.7392 | 14.9528 | 19.7138^*^ |
|  | (-2.2196) | (-3.6794) | (-1.2091) | (1.2193) | (0.7792) | (1.8045) |
| 2018.year | -9.3983^**^ | -13.2432^***^ | -3.7673 | 34.5421 | 18.8607 | 22.0900^*^ |
|  | (-2.1284) | (-3.6941) | (-1.0591) | (1.4180) | (0.9209) | (1.8798) |
| 2019.year | -10.0276^**^ | -15.1053^***^ | -3.2051 | 37.8638 | 18.5887 | 27.2086^**^ |
|  | (-2.0928) | (-3.8692) | (-0.8175) | (1.4475) | (0.8441) | (2.1379) |
| 2020.year | -15.9845^***^ | -17.4083^***^ | -10.2039^***^ | 16.2050 | 10.7002 | 5.8095 |
|  | (-3.1742) | (-4.1849) | (-2.7364) | (0.5911) | (0.4606) | (0.4548) |
| 2021.year | -27.4880^***^ | -26.5592^***^ | -17.6764^***^ | -69.0987^**^ | -53.7181^*^ | -33.8884^**^ |
|  | (-4.7762) | (-5.3161) | (-4.3919) | (-2.0814) | (-1.9070) | (-2.1182) |
| 1.province | 0.0000 | 0.0000 | 0.0000 | 0.0000 | 0.0000 | 0.0000 |
|  | (.) | (.) | (.) | (.) | (.) | (.) |
| 2.province | -6.2194^***^ | -5.0130^**^ | -3.8783^**^ | 14.6917 | 8.3654 | 4.7446 |
|  | (-2.7153) | (-2.4632) | (-2.5655) | (0.9827) | (0.7216) | (0.7481) |
| 3.province | -5.3751^**^ | -5.0051^**^ | -2.7770^*^ | -25.6042^*^ | -22.1534^*^ | -8.4395 |
|  | (-2.0566) | (-2.1191) | (-1.6747) | (-1.8442) | (-1.8958) | (-1.3221) |
| 4.province | -5.2302 | -4.4016 | -0.3070 | -30.0044^*^ | -28.5482^*^ | -10.6206 |
|  | (-1.2906) | (-1.5047) | (-0.1074) | (-1.8353) | (-1.8208) | (-1.3703) |
| 5.province | -2.5103 | -3.2893^**^ | -0.4208 | -12.1181 | -7.9604 | -6.9749 |
|  | (-1.3125) | (-2.0743) | (-0.3052) | (-1.0154) | (-0.8487) | (-1.2189) |
| 6.province | -14.1675^***^ | -9.8485^***^ | -10.4555^***^ | -27.4659^*^ | -16.3375 | -14.8072^**^ |
|  | (-5.2075) | (-4.4144) | (-3.9918) | (-1.8237) | (-1.2886) | (-1.9954) |
| 7.province | -2.7868 | -1.1024 | -2.3036 | -35.2646^*^ | -23.7936 | -20.1309^*^ |
|  | (-0.9240) | (-0.4417) | (-0.9881) | (-1.8885) | (-1.5996) | (-1.9436) |
| 8.province | -11.9556^***^ | -9.6602^***^ | -7.5141^***^ | -23.2154 | -20.5419 | -15.0736 |
|  | (-3.5848) | (-3.1089) | (-2.7584) | (-1.2380) | (-1.5504) | (-1.6357) |
| 9.province | -2.6901 | -1.4861 | -0.5135 | -28.0974^**^ | -22.5506^**^ | -11.1306 |
|  | (-0.8792) | (-0.6111) | (-0.2402) | (-1.9808) | (-1.9856) | (-1.5992) |
| 10.province | -6.4637^**^ | -5.1718^**^ | -2.8640 | 0.9387 | -5.9503 | -2.3525 |
|  | (-2.4743) | (-2.4258) | (-1.4857) | (0.0703) | (-0.4634) | (-0.4152) |
| 11.province | -5.7458^*^ | -4.7976^*^ | -2.2840 | -44.1849^**^ | -30.0019^**^ | -24.0604^**^ |
|  | (-1.7278) | (-1.6980) | (-0.9774) | (-2.3045) | (-2.1655) | (-2.2639) |
| 12.province | -4.5979^**^ | -3.3459^*^ | -2.0218 | -10.2262 | -7.9521 | -3.2738 |
|  | (-2.0037) | (-1.6487) | (-1.2810) | (-0.8483) | (-0.8302) | (-0.5478) |
| 13.province | -4.6492^**^ | -3.1587^*^ | -2.8822^*^ | -22.4829^*^ | -17.2794 | -12.6999^*^ |
|  | (-2.1038) | (-1.6506) | (-1.7882) | (-1.6506) | (-1.5878) | (-1.6719) |
| 14.province | -10.2264^***^ | -7.2711^***^ | -5.6724^**^ | -44.2428^***^ | -35.8162^**^ | -23.4060^***^ |
|  | (-3.2479) | (-3.0196) | (-2.3623) | (-2.7176) | (-2.4733) | (-2.9281) |
| 15.province | -5.7929^***^ | -4.8642^***^ | -2.7455^**^ | -7.7249 | -4.8669 | -4.7183 |
|  | (-3.1584) | (-3.0449) | (-2.1334) | (-0.6140) | (-0.4619) | (-0.8472) |
| 16.province | -4.7660^*^ | -5.2453^**^ | -0.9428 | -22.1510^*^ | -21.6511^*^ | -9.6652 |
|  | (-1.7760) | (-2.3352) | (-0.4753) | (-1.6995) | (-1.8213) | (-1.4930) |
| 17.province | -8.0017^**^ | -7.1487^***^ | -3.3511 | -24.3140^*^ | -17.8998 | -12.3645^*^ |
|  | (-2.4656) | (-2.9187) | (-1.3387) | (-1.6926) | (-1.4453) | (-1.9027) |
| 18.province | -11.0517^***^ | -6.7215^*^ | -7.9625^***^ | -23.7704^*^ | -21.3796^**^ | -5.9392 |
|  | (-2.9337) | (-1.7310) | (-2.7563) | (-1.8429) | (-2.1135) | (-0.9519) |
| 19.province | -11.7124^***^ | -7.5590^**^ | -10.5274^**^ | -27.5884 | -22.7095 | -11.4356 |
|  | (-2.7553) | (-2.0965) | (-2.5370) | (-0.7309) | (-0.8697) | (-0.7742) |
| 20.province | -10.0477^**^ | -4.2463 | -10.7936^***^ | -28.6860^*^ | -19.8774 | -12.6076 |
|  | (-2.2863) | (-1.1815) | (-2.7198) | (-1.9440) | (-1.6213) | (-1.5181) |
| 21.province | -2.3438 | -1.1090 | -1.9280 | -3.9942 | -8.4079 | -0.2710 |
|  | (-1.1505) | (-0.6076) | (-1.4332) | (-0.3100) | (-0.7772) | (-0.0453) |
| 22.province | -8.8520^***^ | -8.9360^***^ | -4.2868^**^ | -42.1605^***^ | -36.1311^***^ | -16.9784^**^ |
|  | (-3.6457) | (-3.5144) | (-2.4213) | (-2.8549) | (-2.7475) | (-2.5514) |
| 23.province | -0.3581 | -2.8480 | 1.0515 | -28.1997^**^ | -23.2861^**^ | -14.2934^*^ |
|  | (-0.1194) | (-1.1928) | (0.5247) | (-2.1253) | (-2.2623) | (-1.9319) |
| 24.province | -8.6407^***^ | -7.3938^***^ | -4.4682^***^ | -33.1760^**^ | -27.5044^**^ | -14.0675^**^ |
|  | (-4.0456) | (-4.0689) | (-2.8124) | (-2.4427) | (-2.3982) | (-2.2313) |
| 25.province | -3.5008 | -2.5664 | -1.2882 | -2.8204 | -1.3831 | -5.5758 |
|  | (-1.5656) | (-1.4115) | (-0.8039) | (-0.2283) | (-0.1418) | (-0.8827) |
| 26.province | -9.4435^***^ | -8.1112^***^ | -4.2692^*^ | -39.2404^**^ | -28.1772^**^ | -14.4849^*^ |
|  | (-3.0875) | (-3.0060) | (-1.9201) | (-2.4021) | (-2.2108) | (-1.7707) |
| 27.province | -20.5202^***^ | -14.3271^***^ | -17.0077^***^ | -47.8357^*^ | -33.9816 | -31.4926^***^ |
|  | (-5.8070) | (-4.2266) | (-3.2825) | (-1.6544) | (-1.3779) | (-2.7647) |
| 28.province | -17.0440^***^ | -13.2043^***^ | -10.7624^***^ | -14.7583 | -13.5572 | -4.9357 |
|  | (-5.3044) | (-5.5570) | (-3.6347) | (-0.7017) | (-0.8570) | (-0.5037) |
| 29.province | -4.8838 | -3.5707 | -3.9640^*^ | -22.3274^*^ | -14.7932 | -15.6790^**^ |
|  | (-1.5545) | (-1.0573) | (-1.8682) | (-1.7677) | (-1.2920) | (-2.5432) |
| 30.province | -7.6162^***^ | -6.5837^***^ | -4.0687^***^ | -13.0744 | -11.3546 | -5.4417 |
|  | (-4.1512) | (-4.0946) | (-3.0867) | (-1.2359) | (-1.3527) | (-1.0152) |
| 31.province | -8.0334^**^ | -5.3434^*^ | -6.3088^**^ | -23.3701^*^ | -20.9295^*^ | -11.2609 |
|  | (-2.1908) | (-1.8476) | (-2.0237) | (-1.7032) | (-1.7462) | (-1.5091) |
| _cons | -1.1e+02^***^ | -89.2711^***^ | -69.5681^***^ | -5.1e+02^***^ | -4.3e+02^***^ | -2.0e+02^***^ |
|  | (-5.9170) | (-6.9592) | (-3.8882) | (-3.7754) | (-3.4198) | (-4.0214) |
| / |  |  |  |  |  |  |
| var(e.PAT) | 208.4403^***^ |  |  | 8.2e+03^***^ |  |  |
|  | (5.3129) |  |  | (3.0556) |  |  |
| var(e.INPAT) |  | 124.7721^***^ |  |  | 4.9e+03^**^ |  |
|  |  | (5.2960) |  |  | (2.4705) |  |
| var(e.UPAT) |  |  | 105.4684^***^ |  |  | 1.5e+03^***^ |
|  |  |  | (2.6579) |  |  | (3.2818) |
| *N* | 12397 | 12397 | 12397 | 10384 | 10384 | 10384 |
| pseudo *R*^2^ | 0.0380 | 0.0412 | 0.0444 | 0.0249 | 0.0262 | 0.0369 |

*t* statistics in parentheses

^*^ *p* < 0.1, ^**^ *p* < 0.05, ^***^ *p* < 0.01

|  | (1) | (2) | (3) | (4) | (5) | (6) |
| --- | --- | --- | --- | --- | --- | --- |
|  | PAT | INPAT | UPAT | PAT | INPAT | UPAT |
| main |  |  |  |  |  |  |
| DIFB | 0.0658^***^ | 0.0714^***^ | 0.0341^***^ | 0.0705 | 0.0848 | 0.0115 |
|  | (3.9989) | (5.1803) | (2.8310) | (0.7697) | (1.0530) | (0.2711) |
| Size | 5.0368^***^ | 4.0521^***^ | 2.9938^***^ | 22.8448^***^ | 17.4822^***^ | 9.2393^***^ |
|  | (7.0017) | (7.1792) | (4.9822) | (3.5303) | (3.2010) | (3.6300) |
| Age | -4.1498^***^ | -3.1242^***^ | -2.7735^***^ | -39.3637^***^ | -26.0572^***^ | -18.2190^***^ |
|  | (-3.1194) | (-2.9373) | (-2.8929) | (-3.2656) | (-3.0480) | (-3.1087) |
| lev | 0.3613 | -1.5522 | 2.1167^**^ | -6.3322 | -11.0104 | 9.8888 |
|  | (0.2337) | (-1.1198) | (2.0342) | (-0.3760) | (-0.8320) | (1.4086) |
| Top1 | -10.2312^**^ | -9.3019^**^ | -5.1504^*^ | 15.1558 | -4.2270 | 23.9681^**^ |
|  | (-2.1674) | (-2.1028) | (-1.7877) | (0.6371) | (-0.2250) | (2.3121) |
| Mfee | -1.5687^*^ | -0.3904 | -2.1538^**^ | -13.4299 | -5.6609 | -9.1807 |
|  | (-1.7060) | (-0.7412) | (-2.5090) | (-1.0370) | (-1.0932) | (-1.2538) |
| Growth | 0.0948 | 0.1024 | -0.0421 | 1.3801 | 1.3575 | -0.4765 |
|  | (0.2782) | (0.3971) | (-0.1710) | (0.5017) | (0.5926) | (-0.3890) |
| ROE | 1.0537 | 0.3923 | 1.2986^*^ | 11.2702 | 11.4839 | 5.2430 |
|  | (1.2104) | (0.5338) | (1.8515) | (1.2048) | (1.5488) | (1.1576) |
| TobinQ | 0.1152 | 0.0367 | -0.0551 | 4.0577 | 2.9407 | 0.8432 |
|  | (0.3297) | (0.1331) | (-0.1469) | (0.6123) | (0.6896) | (0.2274) |
| Mshare | -4.9302 | -1.6106 | -4.0416 | 18.0955 | 22.6255 | 2.7835 |
|  | (-1.0521) | (-0.4255) | (-1.0480) | (0.4759) | (0.8158) | (0.1286) |
| Indep | -1.8997 | -3.8712 | 1.2973 | 90.0169^*^ | 58.5594^*^ | 44.7705^*^ |
|  | (-0.2140) | (-0.7274) | (0.1642) | (1.7602) | (1.6642) | (1.6705) |
| Dual | 0.1962 | 0.4465 | -0.2399 | 9.4444 | 8.7800^**^ | 2.9476 |
|  | (0.3019) | (0.8355) | (-0.4867) | (1.6016) | (1.9718) | (1.1071) |
| SOE | -0.4784 | 0.3554 | -0.9992^*^ | 4.3610 | 4.4745 | 0.4172 |
|  | (-0.5390) | (0.4539) | (-1.6466) | (0.8380) | (1.0598) | (0.1773) |
| Board | 0.8078 | 0.0209 | 1.2955 | 7.9456 | 14.4356 | -4.6371 |
|  | (0.3328) | (0.0122) | (0.6636) | (0.5111) | (1.0687) | (-0.7556) |
| 2011.year | 0.0000 | 0.0000 | 0.0000 | 0.0000 | 0.0000 | 0.0000 |
|  | (.) | (.) | (.) | (.) | (.) | (.) |
| 2012.year | -1.2143 | -1.3609^**^ | -0.5557 | 3.0824 | 1.8003 | 2.4789 |
|  | (-1.4307) | (-1.9717) | (-0.8134) | (0.6580) | (0.4488) | (1.0812) |
| 2013.year | -3.0965^**^ | -3.5723^***^ | -1.5441 | 5.4202 | 5.6309 | 3.3442 |
|  | (-2.3713) | (-3.2962) | (-1.5234) | (0.7482) | (0.9065) | (0.9675) |
| 2014.year | -3.5018^*^ | -4.6178^***^ | -1.5624 | 10.2684 | 7.9018 | 6.4664 |
|  | (-1.7975) | (-3.1355) | (-0.9646) | (0.9733) | (0.8990) | (1.2698) |
| 2015.year | -3.7886 | -5.7283^***^ | -0.9388 | 12.8411 | 8.4933 | 8.9548 |
|  | (-1.6125) | (-3.3155) | (-0.4579) | (1.0270) | (0.8163) | (1.4715) |
| 2016.year | -3.5177 | -5.6879^***^ | -1.0923 | 21.5445 | 15.0883 | 12.7218^*^ |
|  | (-1.4990) | (-3.0410) | (-0.5622) | (1.5025) | (1.2841) | (1.8249) |
| 2017.year | -3.2437 | -5.5685^***^ | -1.2128 | 30.7032^*^ | 21.0821 | 18.0178^**^ |
|  | (-1.2070) | (-2.6214) | (-0.5544) | (1.8695) | (1.5763) | (2.2356) |
| 2018.year | -3.4839 | -6.4602^***^ | -1.0201 | 37.2704^**^ | 24.9048^*^ | 20.1901^**^ |
|  | (-1.1710) | (-2.7550) | (-0.4160) | (2.0578) | (1.6911) | (2.2690) |
| 2019.year | -4.0379 | -8.2044^***^ | -0.4444 | 40.3955^**^ | 24.6090 | 25.0988^**^ |
|  | (-1.2129) | (-3.1195) | (-0.1585) | (2.0248) | (1.5171) | (2.5351) |
| 2020.year | -9.9200^***^ | -10.4115^***^ | -7.4188^***^ | 18.6113 | 16.7267 | 3.5409 |
|  | (-2.8003) | (-3.6402) | (-2.8767) | (0.8785) | (0.9608) | (0.3590) |
| 2021.year | -21.5989^***^ | -19.7180^***^ | -15.0201^***^ | -67.4305^**^ | -48.1705^**^ | -36.6192^***^ |
|  | (-4.9450) | (-5.2139) | (-4.9872) | (-2.4282) | (-2.1130) | (-2.6252) |
| 1.province | 0.0000 | 0.0000 | 0.0000 | 0.0000 | 0.0000 | 0.0000 |
|  | (.) | (.) | (.) | (.) | (.) | (.) |
| 2.province | -6.4033^***^ | -5.1579^**^ | -4.0298^***^ | 13.9218 | 7.8744 | 4.2510 |
|  | (-2.7825) | (-2.5273) | (-2.6487) | (0.9272) | (0.6802) | (0.6610) |
| 3.province | -5.5981^**^ | -5.2162^**^ | -2.9282^*^ | -26.1646^*^ | -22.5861^*^ | -8.7339 |
|  | (-2.1443) | (-2.2106) | (-1.7629) | (-1.8665) | (-1.9119) | (-1.3580) |
| 4.province | -6.4757 | -5.8028^**^ | -0.9394 | -30.8005^*^ | -29.7569^*^ | -10.5418 |
|  | (-1.5920) | (-1.9634) | (-0.3278) | (-1.8720) | (-1.8783) | (-1.3548) |
| 5.province | -2.9003 | -3.6900^**^ | -0.6683 | -13.0229 | -8.7082 | -7.4210 |
|  | (-1.4960) | (-2.3048) | (-0.4757) | (-1.0800) | (-0.9105) | (-1.2836) |
| 6.province | -14.6407^***^ | -10.3848^***^ | -10.6948^***^ | -27.7955^*^ | -16.9315 | -14.7280^**^ |
|  | (-5.3791) | (-4.6200) | (-4.1169) | (-1.8514) | (-1.3401) | (-1.9939) |
| 7.province | -3.6971 | -2.1144 | -2.7712 | -36.1433^*^ | -25.0062^*^ | -20.1528^*^ |
|  | (-1.2336) | (-0.8508) | (-1.1993) | (-1.9309) | (-1.6763) | (-1.9466) |
| 8.province | -12.7911^***^ | -10.5738^***^ | -7.9612^***^ | -24.3706 | -21.8423 | -15.3713^*^ |
|  | (-3.8364) | (-3.3743) | (-2.9488) | (-1.2883) | (-1.6269) | (-1.6543) |
| 9.province | -3.2557 | -2.1529 | -0.7652 | -28.4017^**^ | -23.1785^**^ | -10.9712 |
|  | (-1.0699) | (-0.8879) | (-0.3615) | (-2.0115) | (-2.0510) | (-1.5821) |
| 10.province | -7.1577^***^ | -5.9412^***^ | -3.2225^*^ | 0.3361 | -6.8233 | -2.3487 |
|  | (-2.7653) | (-2.7896) | (-1.6964) | (0.0250) | (-0.5261) | (-0.4130) |
| 11.province | -6.3170^*^ | -5.4324^*^ | -2.5614 | -44.5012^**^ | -30.6554^**^ | -23.8758^**^ |
|  | (-1.9018) | (-1.9230) | (-1.0999) | (-2.3275) | (-2.2189) | (-2.2560) |
| 12.province | -4.8302^**^ | -3.5877^*^ | -2.1579 | -10.5951 | -8.3197 | -3.4006 |
|  | (-2.1025) | (-1.7653) | (-1.3638) | (-0.8715) | (-0.8610) | (-0.5650) |
| 13.province | -4.9260^**^ | -3.4760^*^ | -3.0318^*^ | -22.7204^*^ | -17.6178 | -12.7046^*^ |
|  | (-2.2307) | (-1.8116) | (-1.8889) | (-1.6630) | (-1.6119) | (-1.6684) |
| 14.province | -10.9797^***^ | -8.1265^***^ | -6.0449^**^ | -44.7504^***^ | -36.6761^**^ | -23.2817^***^ |
|  | (-3.5039) | (-3.3651) | (-2.5510) | (-2.7632) | (-2.5439) | (-2.9258) |
| 15.province | -5.9914^***^ | -5.0520^***^ | -2.8851^**^ | -8.2674 | -5.2597 | -5.0330 |
|  | (-3.2461) | (-3.1353) | (-2.2309) | (-0.6514) | (-0.4943) | (-0.8962) |
| 16.province | -4.8120^*^ | -5.3159^**^ | -0.9533 | -22.2240^*^ | -21.7782^*^ | -9.6434 |
|  | (-1.7882) | (-2.3667) | (-0.4789) | (-1.7082) | (-1.8346) | (-1.4934) |
| 17.province | -8.5331^***^ | -7.7268^***^ | -3.6147 | -24.8471^*^ | -18.5946 | -12.4092^*^ |
|  | (-2.6359) | (-3.1427) | (-1.4527) | (-1.7267) | (-1.4952) | (-1.9110) |
| 18.province | -11.9818^***^ | -7.8365^**^ | -8.3990^***^ | -24.6302^*^ | -22.6230^**^ | -5.9356 |
|  | (-3.2121) | (-2.0198) | (-2.9658) | (-1.8987) | (-2.2190) | (-0.9502) |
| 19.province | -13.1345^***^ | -9.0800^**^ | -11.2631^***^ | -28.9631 | -24.4018 | -11.6077 |
|  | (-3.1140) | (-2.5308) | (-2.7485) | (-0.7647) | (-0.9284) | (-0.7834) |
| 20.province | -11.1415^**^ | -5.5395 | -11.2083^***^ | -29.6038^**^ | -21.2986^*^ | -12.5009 |
|  | (-2.5093) | (-1.5102) | (-2.8981) | (-2.0151) | (-1.7474) | (-1.5117) |
| 21.province | -2.7519 | -1.5600 | -2.1392 | -4.3887 | -8.9449 | -0.2887 |
|  | (-1.3546) | (-0.8582) | (-1.5951) | (-0.3411) | (-0.8277) | (-0.0483) |
| 22.province | -9.8306^***^ | -10.0258^***^ | -4.7703^***^ | -43.0108^***^ | -37.3180^***^ | -16.9912^**^ |
|  | (-4.0789) | (-3.9217) | (-2.7599) | (-2.9101) | (-2.8347) | (-2.5581) |
| 23.province | -1.1353 | -3.7142 | 0.6432 | -29.0775^**^ | -24.3151^**^ | -14.4717^*^ |
|  | (-0.3801) | (-1.5523) | (0.3206) | (-2.1618) | (-2.3189) | (-1.9396) |
| 24.province | -8.2003^***^ | -6.8389^***^ | -4.3160^***^ | -33.3283^**^ | -27.1969^**^ | -14.4932^**^ |
|  | (-3.9369) | (-3.8878) | (-2.7558) | (-2.4608) | (-2.3792) | (-2.3165) |
| 25.province | -3.8554^*^ | -2.9742 | -1.4655 | -3.1287 | -1.8391 | -5.5582 |
|  | (-1.7294) | (-1.6346) | (-0.9198) | (-0.2532) | (-0.1886) | (-0.8812) |
| 26.province | -9.6955^***^ | -8.3882^***^ | -4.4060^**^ | -39.5453^**^ | -28.5232^**^ | -14.5550^*^ |
|  | (-3.1703) | (-3.1030) | (-1.9847) | (-2.4157) | (-2.2306) | (-1.7779) |
| 27.province | -21.3998^***^ | -15.2942^***^ | -17.4641^***^ | -48.6179^*^ | -35.0134 | -31.5620^***^ |
|  | (-6.0925) | (-4.4906) | (-3.3980) | (-1.6769) | (-1.4148) | (-2.7684) |
| 28.province | -18.2651^***^ | -14.5913^***^ | -11.3574^***^ | -15.9482 | -15.0774 | -5.0726 |
|  | (-5.6851) | (-5.9911) | (-3.8958) | (-0.7579) | (-0.9489) | (-0.5181) |
| 29.province | -5.4495^*^ | -4.2038 | -4.2270^**^ | -23.1287^*^ | -15.7239 | -15.8282^**^ |
|  | (-1.7243) | (-1.2314) | (-2.0060) | (-1.8008) | (-1.3536) | (-2.5253) |
| 30.province | -7.4154^***^ | -6.3107^***^ | -4.0130^***^ | -13.3138 | -11.2537 | -5.8067 |
|  | (-4.0674) | (-3.9599) | (-3.0535) | (-1.2630) | (-1.3432) | (-1.0888) |
| 31.province | -8.4689^**^ | -5.8373^**^ | -6.5143^**^ | -23.6634^*^ | -21.4247^*^ | -11.1996 |
|  | (-2.3101) | (-2.0126) | (-2.0953) | (-1.7249) | (-1.7880) | (-1.5019) |
| _cons | -1.1e+02^***^ | -87.9184^***^ | -69.0724^***^ | -5.1e+02^***^ | -4.3e+02^***^ | -2.0e+02^***^ |
|  | (-5.8417) | (-6.8959) | (-3.8377) | (-3.7680) | (-3.4091) | (-4.0295) |
| / |  |  |  |  |  |  |
| var(e.PAT) | 208.4682^***^ |  |  | 8.2e+03^***^ |  |  |
|  | (5.3101) |  |  | (3.0558) |  |  |
| var(e.INPAT) |  | 124.8340^***^ |  |  | 4.9e+03^**^ |  |
|  |  | (5.2985) |  |  | (2.4709) |  |
| var(e.UPAT) |  |  | 105.4751^***^ |  |  | 1.5e+03^***^ |
|  |  |  | (2.6560) |  |  | (3.2817) |
| *N* | 12397 | 12397 | 12397 | 10384 | 10384 | 10384 |
| pseudo *R*^2^ | 0.0380 | 0.0412 | 0.0445 | 0.0249 | 0.0262 | 0.0369 |

*t* statistics in parentheses

^*^ *p* < 0.1, ^**^ *p* < 0.05, ^***^ *p* < 0.01

|  | (1) | (2) | (3) | (4) | (5) | (6) |
| --- | --- | --- | --- | --- | --- | --- |
|  | PAT | INPAT | UPAT | PAT | INPAT | UPAT |
| main |  |  |  |  |  |  |
| DIFD | 0.0689^***^ | 0.0816^***^ | 0.0312^*^ | 0.0442 | 0.0770 | -0.0125 |
|  | (3.0801) | (4.4065) | (1.7665) | (0.4073) | (0.8122) | (-0.2483) |
| Size | 4.9985^***^ | 4.0076^***^ | 2.9753^***^ | 22.8352^***^ | 17.4704^***^ | 9.2389^***^ |
|  | (6.9560) | (7.1317) | (4.9448) | (3.5264) | (3.1964) | (3.6289) |
| Age | -4.3868^***^ | -3.3756^***^ | -2.9001^***^ | -39.2141^***^ | -25.9089^***^ | -18.1714^***^ |
|  | (-3.2689) | (-3.1288) | (-3.0124) | (-3.2513) | (-3.0367) | (-3.0967) |
| lev | 0.5357 | -1.3756 | 2.2232^**^ | -5.7805 | -10.4742 | 10.0740 |
|  | (0.3460) | (-0.9946) | (2.1287) | (-0.3473) | (-0.8019) | (1.4485) |
| Top1 | -9.6842^**^ | -8.8036^**^ | -4.8243^*^ | 15.5182 | -3.8153 | 24.0255^**^ |
|  | (-2.0545) | (-1.9960) | (-1.6717) | (0.6548) | (-0.2040) | (2.3216) |
| Mfee | -1.5518^*^ | -0.3889 | -2.1116^**^ | -13.3422 | -5.5815 | -9.1725 |
|  | (-1.7099) | (-0.7351) | (-2.5196) | (-1.0324) | (-1.0979) | (-1.2536) |
| Growth | 0.1568 | 0.1681 | -0.0070 | 1.3814 | 1.3506 | -0.4742 |
|  | (0.4630) | (0.6680) | (-0.0286) | (0.5021) | (0.5896) | (-0.3873) |
| ROE | 1.0764 | 0.4493 | 1.2898^*^ | 11.5172 | 11.8156 | 5.2613 |
|  | (1.2220) | (0.6137) | (1.8368) | (1.2236) | (1.5792) | (1.1599) |
| TobinQ | 0.0803 | -0.0029 | -0.0776 | 4.0507 | 2.9427 | 0.8367 |
|  | (0.2262) | (-0.0103) | (-0.2049) | (0.6115) | (0.6897) | (0.2261) |
| Mshare | -5.3387 | -2.0051 | -4.2730 | 17.7663 | 22.1640 | 2.6445 |
|  | (-1.1273) | (-0.5222) | (-1.0999) | (0.4704) | (0.8068) | (0.1227) |
| Indep | -1.4035 | -3.3425 | 1.5648 | 89.5981^*^ | 58.0641^*^ | 44.6473^*^ |
|  | (-0.1603) | (-0.6279) | (0.2003) | (1.7508) | (1.6494) | (1.6654) |
| Dual | 0.2857 | 0.5359 | -0.1877 | 9.4295 | 8.7723^**^ | 2.9481 |
|  | (0.4371) | (1.0014) | (-0.3780) | (1.5974) | (1.9658) | (1.1068) |
| SOE | -0.5135 | 0.3154 | -1.0200^*^ | 4.3644 | 4.4793 | 0.4211 |
|  | (-0.5808) | (0.4042) | (-1.6900) | (0.8379) | (1.0597) | (0.1788) |
| Board | 0.9087 | 0.1545 | 1.3260 | 7.7418 | 14.2425 | -4.7106 |
|  | (0.3772) | (0.0907) | (0.6836) | (0.4987) | (1.0569) | (-0.7682) |
| 2011.year | 0.0000 | 0.0000 | 0.0000 | 0.0000 | 0.0000 | 0.0000 |
|  | (.) | (.) | (.) | (.) | (.) | (.) |
| 2012.year | -1.5920 | -2.0901^**^ | -0.5377 | 4.1118 | 1.9442 | 3.5708 |
|  | (-1.3892) | (-2.2500) | (-0.5623) | (0.7317) | (0.3991) | (1.3093) |
| 2013.year | -4.5602^**^ | -5.7929^***^ | -1.9010 | 6.4717 | 4.7371 | 5.3248 |
|  | (-2.1799) | (-3.3591) | (-1.1122) | (0.6246) | (0.5249) | (1.0786) |
| 2014.year | -2.0594 | -3.6155^**^ | -0.4583 | 14.0860 | 10.5818 | 8.6967^*^ |
|  | (-1.0283) | (-2.3669) | (-0.2567) | (1.4302) | (1.2558) | (1.8163) |
| 2015.year | -2.5367 | -5.0632^***^ | 0.1538 | 17.1168 | 11.1994 | 11.6824^**^ |
|  | (-0.9947) | (-2.6561) | (0.0651) | (1.3952) | (1.0677) | (1.9662) |
| 2016.year | -4.4127 | -7.6361^***^ | -0.9140 | 24.7538 | 15.4962 | 16.1315^*^ |
|  | (-1.3370) | (-2.8786) | (-0.3218) | (1.4322) | (1.0562) | (1.9328) |
| 2017.year | -6.6538 | -10.6465^***^ | -2.0878 | 32.7539 | 18.7565 | 22.3359^**^ |
|  | (-1.4654) | (-2.9184) | (-0.5473) | (1.4000) | (0.9396) | (1.9853) |
| 2018.year | -5.2231 | -9.6793^***^ | -1.0549 | 40.9130^*^ | 24.6523 | 24.6334^**^ |
|  | (-1.1881) | (-2.7535) | (-0.2802) | (1.7893) | (1.2735) | (2.2246) |
| 2019.year | -5.2030 | -10.8787^***^ | -0.1368 | 44.9287^*^ | 25.1916 | 29.8845^**^ |
|  | (-1.1166) | (-2.9112) | (-0.0337) | (1.8797) | (1.2453) | (2.5363) |
| 2020.year | -10.7942^**^ | -12.8525^***^ | -6.9233^*^ | 23.6932 | 17.7196 | 8.6231 |
|  | (-2.2202) | (-3.2462) | (-1.8146) | (0.9570) | (0.8394) | (0.7413) |
| 2021.year | -20.9435^***^ | -20.5622^***^ | -13.6790^***^ | -60.1385^**^ | -44.9011^*^ | -30.8644^**^ |
|  | (-3.9546) | (-4.5445) | (-3.5362) | (-2.0219) | (-1.8202) | (-2.1457) |
| 1.province | 0.0000 | 0.0000 | 0.0000 | 0.0000 | 0.0000 | 0.0000 |
|  | (.) | (.) | (.) | (.) | (.) | (.) |
| 2.province | -4.9059^**^ | -3.6797^*^ | -3.1314^**^ | 16.1113 | 9.9946 | 5.0186 |
|  | (-2.1922) | (-1.8412) | (-2.1327) | (1.1043) | (0.8753) | (0.8233) |
| 3.province | -4.4504^*^ | -4.0213^*^ | -2.2683 | -24.6748^*^ | -21.0042^*^ | -8.3350 |
|  | (-1.6812) | (-1.6759) | (-1.3687) | (-1.8004) | (-1.8199) | (-1.3246) |
| 4.province | -3.1675 | -1.8783 | 0.6168 | -29.2599^*^ | -26.8222^*^ | -11.2092 |
|  | (-0.7603) | (-0.6227) | (0.2095) | (-1.7296) | (-1.6739) | (-1.4003) |
| 5.province | -1.1773 | -1.8268 | 0.3034 | -10.6860 | -6.1342 | -6.8428 |
|  | (-0.6277) | (-1.1644) | (0.2291) | (-0.9306) | (-0.6881) | (-1.2329) |
| 6.province | -13.5289^***^ | -8.9966^***^ | -10.1883^***^ | -27.3324^*^ | -15.7692 | -15.1012^**^ |
|  | (-4.9458) | (-4.0842) | (-3.7719) | (-1.7903) | (-1.2218) | (-2.0169) |
| 7.province | -1.2612 | 0.8045 | -1.6066 | -34.4327^*^ | -22.0966 | -20.5807^*^ |
|  | (-0.3966) | (0.3082) | (-0.6481) | (-1.8083) | (-1.4467) | (-1.9573) |
| 8.province | -10.1203^***^ | -7.5413^**^ | -6.6292^**^ | -21.8279 | -18.3969 | -15.2689^*^ |
|  | (-2.9916) | (-2.4344) | (-2.3494) | (-1.1641) | (-1.3837) | (-1.6576) |
| 9.province | -2.6175 | -1.2218 | -0.5600 | -28.1898^*^ | -22.2513^*^ | -11.4935 |
|  | (-0.8371) | (-0.4920) | (-0.2540) | (-1.9552) | (-1.9186) | (-1.6302) |
| 10.province | -5.1951^*^ | -3.6027 | -2.2808 | 1.5067 | -4.7186 | -2.7022 |
|  | (-1.8773) | (-1.6125) | (-1.1030) | (0.1114) | (-0.3623) | (-0.4692) |
| 11.province | -4.8195 | -3.6378 | -1.8670 | -44.1526^**^ | -29.4749^**^ | -24.4976^**^ |
|  | (-1.4133) | (-1.2608) | (-0.7743) | (-2.2787) | (-2.0986) | (-2.2862) |
| 12.province | -3.8991^*^ | -2.5360 | -1.6649 | -9.6772 | -7.1597 | -3.3040 |
|  | (-1.6842) | (-1.2377) | (-1.0511) | (-0.8105) | (-0.7532) | (-0.5583) |
| 13.province | -4.2689^*^ | -2.6390 | -2.6876 | -22.3404 | -16.9443 | -12.8009^*^ |
|  | (-1.9033) | (-1.3640) | (-1.6354) | (-1.6425) | (-1.5597) | (-1.6850) |
| 14.province | -9.5561^***^ | -6.3673^***^ | -5.3963^**^ | -44.1424^***^ | -35.1848^**^ | -23.8243^***^ |
|  | (-2.9668) | (-2.6045) | (-2.1488) | (-2.6510) | (-2.3715) | (-2.9313) |
| 15.province | -4.9431^***^ | -3.9789^**^ | -2.2554^*^ | -6.8301 | -3.8169 | -4.5609 |
|  | (-2.7174) | (-2.5311) | (-1.7700) | (-0.5443) | (-0.3611) | (-0.8348) |
| 16.province | -4.9047^*^ | -5.2999^**^ | -1.0272 | -22.0909^*^ | -21.5257^*^ | -9.7422 |
|  | (-1.8419) | (-2.3415) | (-0.5228) | (-1.6856) | (-1.7986) | (-1.5024) |
| 17.province | -7.4062^**^ | -6.3849^***^ | -3.0769 | -23.8590 | -17.0178 | -12.5736^*^ |
|  | (-2.2640) | (-2.6235) | (-1.2045) | (-1.6438) | (-1.3624) | (-1.9140) |
| 18.province | -9.5468^**^ | -4.8621 | -7.3377^**^ | -22.8152^*^ | -19.4167^*^ | -6.4859 |
|  | (-2.5508) | (-1.3167) | (-2.4091) | (-1.6754) | (-1.7948) | (-0.9929) |
| 19.province | -10.2274^**^ | -5.6483 | -9.9307^**^ | -26.2804 | -20.3192 | -11.9102 |
|  | (-2.3964) | (-1.5819) | (-2.3444) | (-0.6933) | (-0.7729) | (-0.8007) |
| 20.province | -9.3968^**^ | -3.1739 | -10.5386^**^ | -28.1258^*^ | -18.3145 | -13.2495 |
|  | (-2.2314) | (-0.9648) | (-2.4616) | (-1.8324) | (-1.4232) | (-1.5490) |
| 21.province | -1.7245 | -0.3515 | -1.6391 | -3.6707 | -7.7451 | -0.4518 |
|  | (-0.8300) | (-0.1885) | (-1.1869) | (-0.2806) | (-0.7040) | (-0.0747) |
| 22.province | -7.3575^***^ | -7.0548^***^ | -3.6265^*^ | -41.4020^***^ | -34.5141^**^ | -17.4285^**^ |
|  | (-2.8119) | (-2.7132) | (-1.8155) | (-2.7332) | (-2.5407) | (-2.5574) |
| 23.province | 1.1504 | -1.0380 | 1.7629 | -27.1329^**^ | -21.5138^**^ | -14.5492^*^ |
|  | (0.3738) | (-0.4236) | (0.8633) | (-2.0505) | (-2.0851) | (-1.9601) |
| 24.province | -8.3076^***^ | -7.2706^***^ | -4.1442^**^ | -32.3582^**^ | -27.0143^**^ | -13.5591^**^ |
|  | (-3.7684) | (-3.8566) | (-2.5675) | (-2.4058) | (-2.3865) | (-2.1502) |
| 25.province | -3.0500 | -1.9852 | -1.0857 | -2.6314 | -0.9138 | -5.7631 |
|  | (-1.3365) | (-1.0794) | (-0.6613) | (-0.2111) | (-0.0925) | (-0.9056) |
| 26.province | -8.8982^***^ | -7.4961^***^ | -3.9849^*^ | -38.9034^**^ | -27.6496^**^ | -14.5479^*^ |
|  | (-2.8900) | (-2.7645) | (-1.7767) | (-2.3821) | (-2.1695) | (-1.7791) |
| 27.province | -19.1029^***^ | -12.6074^***^ | -16.3722^***^ | -46.9524 | -32.3035 | -31.8716^***^ |
|  | (-5.1905) | (-3.6942) | (-3.0561) | (-1.6173) | (-1.3038) | (-2.7753) |
| 28.province | -15.2537^***^ | -11.0356^***^ | -9.9539^***^ | -13.6182 | -11.4340 | -5.3735 |
|  | (-4.5899) | (-4.6616) | (-3.1548) | (-0.6380) | (-0.7074) | (-0.5420) |
| 29.province | -4.4703 | -2.9724 | -3.7773^*^ | -21.4400^*^ | -13.3330 | -15.8885^***^ |
|  | (-1.4628) | (-0.9253) | (-1.7171) | (-1.7043) | (-1.1672) | (-2.5887) |
| 30.province | -7.4140^***^ | -6.5072^***^ | -3.8594^***^ | -12.4029 | -10.9251 | -5.0323 |
|  | (-3.9846) | (-3.9861) | (-2.9275) | (-1.1747) | (-1.3094) | (-0.9378) |
| 31.province | -7.5497^**^ | -4.7163 | -6.1013^*^ | -23.2784^*^ | -20.5280^*^ | -11.4911 |
|  | (-2.0413) | (-1.6181) | (-1.9327) | (-1.6822) | (-1.6945) | (-1.5315) |
| _cons | -1.1e+02^***^ | -88.1990^***^ | -68.6784^***^ | -5.1e+02^***^ | -4.3e+02^***^ | -2.0e+02^***^ |
|  | (-5.8844) | (-6.8985) | (-3.8809) | (-3.7740) | (-3.4225) | (-4.0058) |
| / |  |  |  |  |  |  |
| var(e.PAT) | 209.2705^***^ |  |  | 8.2e+03^***^ |  |  |
|  | (5.3473) |  |  | (3.0556) |  |  |
| var(e.INPAT) |  | 125.5409^***^ |  |  | 4.9e+03^**^ |  |
|  |  | (5.3072) |  |  | (2.4704) |  |
| var(e.UPAT) |  |  | 105.6197^***^ |  |  | 1.5e+03^***^ |
|  |  |  | (2.6712) |  |  | (3.2820) |
| *N* | 12397 | 12397 | 12397 | 10384 | 10384 | 10384 |
| pseudo *R*^2^ | 0.0372 | 0.0398 | 0.0439 | 0.0249 | 0.0262 | 0.0369 |

*t* statistics in parentheses

^*^ *p* < 0.1, ^**^ *p* < 0.05, ^***^ *p* < 0.01

|  | (1) | (2) | (3) | (4) | (5) | (6) |
| --- | --- | --- | --- | --- | --- | --- |
|  | PAT | INPAT | UPAT | PAT | INPAT | UPAT |
| main |  |  |  |  |  |  |
| DIFL | 0.0229^**^ | 0.0264^***^ | 0.0072 | -0.0159 | 0.0126 | -0.0293 |
|  | (2.3163) | (2.9670) | (0.9498) | (-0.3085) | (0.2822) | (-1.2678) |
| Size | 5.0053^***^ | 4.0058^***^ | 2.9812^***^ | 22.8410^***^ | 17.4600^***^ | 9.2518^***^ |
|  | (6.9636) | (7.1105) | (4.9740) | (3.5252) | (3.1945) | (3.6312) |
| Age | -4.5447^***^ | -3.5656^***^ | -2.9744^***^ | -39.1379^***^ | -25.8065^***^ | -18.1536^***^ |
|  | (-3.3700) | (-3.2909) | (-3.0891) | (-3.2437) | (-3.0265) | (-3.0915) |
| lev | 0.5612 | -1.3285 | 2.2351^**^ | -5.5233 | -10.1197 | 10.0780 |
|  | (0.3617) | (-0.9555) | (2.1364) | (-0.3319) | (-0.7767) | (1.4458) |
| Top1 | -9.4840^**^ | -8.5743^*^ | -4.7049 | 15.5305 | -3.7533 | 23.9826^**^ |
|  | (-2.0027) | (-1.9388) | (-1.6122) | (0.6548) | (-0.2006) | (2.3167) |
| Mfee | -1.3881 | -0.2636 | -2.0006^**^ | -13.3269 | -5.6118 | -9.1302 |
|  | (-1.6093) | (-0.5385) | (-2.4440) | (-1.0344) | (-1.1072) | (-1.2486) |
| Growth | 0.1804 | 0.1958 | 0.0051 | 1.3783 | 1.3566 | -0.4847 |
|  | (0.5294) | (0.7738) | (0.0207) | (0.5005) | (0.5918) | (-0.3955) |
| ROE | 0.9551 | 0.3235 | 1.2280^*^ | 11.3989 | 11.7928 | 5.1459 |
|  | (1.0924) | (0.4454) | (1.7566) | (1.2109) | (1.5742) | (1.1370) |
| TobinQ | 0.0954 | 0.0244 | -0.0759 | 4.0342 | 2.9169 | 0.8382 |
|  | (0.2667) | (0.0860) | (-0.1977) | (0.6098) | (0.6853) | (0.2268) |
| Mshare | -4.5894 | -1.1830 | -3.9625 | 17.8702 | 21.7916 | 3.0695 |
|  | (-0.9646) | (-0.3083) | (-1.0128) | (0.4729) | (0.7940) | (0.1423) |
| Indep | -1.4628 | -3.3573 | 1.5332 | 89.5853^*^ | 57.9212^*^ | 44.7866^*^ |
|  | (-0.1671) | (-0.6250) | (0.1961) | (1.7514) | (1.6465) | (1.6699) |
| Dual | 0.2970 | 0.5413 | -0.1808 | 9.4352 | 8.7829^**^ | 2.9544 |
|  | (0.4529) | (1.0080) | (-0.3630) | (1.5984) | (1.9698) | (1.1089) |
| SOE | -0.5410 | 0.2849 | -1.0331^*^ | 4.3815 | 4.4862 | 0.4398 |
|  | (-0.6099) | (0.3645) | (-1.7098) | (0.8408) | (1.0608) | (0.1867) |
| Board | 0.8337 | 0.0787 | 1.2862 | 7.6806 | 14.0980 | -4.6546 |
|  | (0.3442) | (0.0457) | (0.6595) | (0.4948) | (1.0457) | (-0.7601) |
| 2011.year | 0.0000 | 0.0000 | 0.0000 | 0.0000 | 0.0000 | 0.0000 |
|  | (.) | (.) | (.) | (.) | (.) | (.) |
| 2012.year | 0.3861 | 0.3068 | 0.5307 | 7.0550^*^ | 4.8510 | 4.5959^**^ |
|  | (0.5451) | (0.4756) | (0.9352) | (1.8696) | (1.4547) | (2.5160) |
| 2013.year | -1.1659 | -1.6566 | 0.0342 | 12.5296^*^ | 10.1989 | 7.8806^**^ |
|  | (-0.8672) | (-1.3709) | (0.0334) | (1.7446) | (1.5816) | (2.3777) |
| 2014.year | 0.8409 | -0.0684 | 1.2246 | 19.5523^***^ | 15.3567^**^ | 11.1295^***^ |
|  | (0.6580) | (-0.0599) | (1.1991) | (2.6409) | (2.3294) | (3.1493) |
| 2015.year | 0.0813 | -1.7875 | 1.9553 | 24.7416^**^ | 16.6762^*^ | 16.0683^***^ |
|  | (0.0409) | (-1.0028) | (1.2331) | (2.2721) | (1.7365) | (3.1480) |
| 2016.year | 1.0685 | -0.9725 | 2.2064 | 34.4194^***^ | 24.2022^**^ | 20.2072^***^ |
|  | (0.5171) | (-0.5347) | (1.4021) | (2.8419) | (2.3042) | (3.4753) |
| 2017.year | 2.1735 | -0.0004 | 2.6122 | 45.1751^***^ | 31.5211^***^ | 26.2628^***^ |
|  | (0.9442) | (-0.0002) | (1.5120) | (3.2995) | (2.6324) | (3.9740) |
| 2018.year | 2.3298 | -0.5211 | 3.1853 | 53.5917^***^ | 36.4360^***^ | 29.6834^***^ |
|  | (0.8925) | (-0.2257) | (1.6025) | (3.4568) | (2.7047) | (4.0130) |
| 2019.year | 2.8320 | -1.1396 | 4.3511^**^ | 58.1944^***^ | 37.6400^***^ | 35.0712^***^ |
|  | (1.0494) | (-0.4741) | (2.0906) | (3.7143) | (2.7957) | (4.4359) |
| 2020.year | -2.1402 | -2.3702 | -2.1335 | 37.4767^**^ | 30.9139^**^ | 13.7917^**^ |
|  | (-0.7590) | (-0.9560) | (-0.9583) | (2.5096) | (2.3489) | (1.9830) |
| 2021.year | -11.4362^***^ | -9.0925^***^ | -8.4703^***^ | -45.5466^**^ | -30.5275^**^ | -25.7031^***^ |
|  | (-3.5047) | (-3.2384) | (-2.9430) | (-2.2661) | (-1.9645) | (-2.5955) |
| 1.province | 0.0000 | 0.0000 | 0.0000 | 0.0000 | 0.0000 | 0.0000 |
|  | (.) | (.) | (.) | (.) | (.) | (.) |
| 2.province | -3.6179^*^ | -2.1296 | -2.5419^*^ | 17.0539 | 11.5704 | 4.7957 |
|  | (-1.6475) | (-1.0863) | (-1.7351) | (1.2015) | (1.0367) | (0.8138) |
| 3.province | -4.0049 | -3.4283 | -2.0734 | -24.3197^*^ | -20.4059^*^ | -8.4163 |
|  | (-1.4933) | (-1.4091) | (-1.2419) | (-1.7784) | (-1.7688) | (-1.3423) |
| 4.province | -6.0076 | -5.2070^*^ | -0.6721 | -31.2191^*^ | -30.5264^*^ | -10.4517 |
|  | (-1.4576) | (-1.7230) | (-0.2329) | (-1.9017) | (-1.9552) | (-1.3343) |
| 5.province | -0.6760 | -1.1883 | 0.5221 | -10.2493 | -5.3748 | -6.9767 |
|  | (-0.3604) | (-0.7561) | (0.3980) | (-0.9118) | (-0.6159) | (-1.2768) |
| 6.province | -15.2040^***^ | -10.9565^***^ | -10.9485^***^ | -28.4030^*^ | -17.6026 | -14.8484^**^ |
|  | (-5.6900) | (-4.9246) | (-4.3035) | (-1.8673) | (-1.3673) | (-1.9982) |
| 7.province | -3.7471 | -2.1354 | -2.6907 | -36.0016^*^ | -24.8318^*^ | -20.1318^*^ |
|  | (-1.1802) | (-0.8146) | (-1.1083) | (-1.9146) | (-1.6487) | (-1.9430) |
| 8.province | -11.3156^***^ | -8.9378^***^ | -7.1617^***^ | -22.5951 | -19.6489 | -15.1469^*^ |
|  | (-3.3538) | (-2.8559) | (-2.6053) | (-1.2055) | (-1.4815) | (-1.6464) |
| 9.province | -4.7463 | -3.7489 | -1.5372 | -29.5303^**^ | -24.3706^**^ | -11.3206 |
|  | (-1.5521) | (-1.5148) | (-0.7338) | (-2.0762) | (-2.1285) | (-1.6303) |
| 10.province | -6.9704^**^ | -5.6631^**^ | -3.0613 | 0.3011 | -6.8866 | -2.3293 |
|  | (-2.5553) | (-2.5024) | (-1.5519) | (0.0224) | (-0.5310) | (-0.4090) |
| 11.province | -6.7038^**^ | -5.8511^**^ | -2.6981 | -45.6022^**^ | -32.0227^**^ | -24.1494^**^ |
|  | (-1.9813) | (-2.0124) | (-1.1499) | (-2.3732) | (-2.3130) | (-2.2794) |
| 12.province | -4.2185^*^ | -2.8576 | -1.8079 | -9.7650 | -7.3948 | -3.2316 |
|  | (-1.8004) | (-1.3663) | (-1.1423) | (-0.8175) | (-0.7781) | (-0.5451) |
| 13.province | -4.8206^**^ | -3.2431^*^ | -2.9361^*^ | -22.7343^*^ | -17.4936 | -12.8071^*^ |
|  | (-2.1348) | (-1.6490) | (-1.8053) | (-1.6707) | (-1.6068) | (-1.6852) |
| 14.province | -11.7707^***^ | -8.9595^***^ | -6.4116^***^ | -45.6919^***^ | -37.6291^**^ | -23.6486^***^ |
|  | (-3.7541) | (-3.6651) | (-2.7317) | (-2.7739) | (-2.5612) | (-2.9520) |
| 15.province | -4.2217^**^ | -3.1124^**^ | -1.9238 | -6.3340 | -2.9329 | -4.7468 |
|  | (-2.3125) | (-1.9909) | (-1.4945) | (-0.5011) | (-0.2726) | (-0.8804) |
| 16.province | -5.5331^**^ | -6.0009^***^ | -1.3010 | -22.3506^*^ | -22.0795^*^ | -9.6284 |
|  | (-2.0752) | (-2.6217) | (-0.6629) | (-1.6993) | (-1.8400) | (-1.4842) |
| 17.province | -8.6514^***^ | -7.8252^***^ | -3.6405 | -24.6469^*^ | -18.2369 | -12.4762^*^ |
|  | (-2.6918) | (-3.2052) | (-1.4726) | (-1.7039) | (-1.4577) | (-1.9172) |
| 18.province | -12.9213^***^ | -8.8523^**^ | -8.8281^***^ | -24.5949^*^ | -22.8568^**^ | -5.7435 |
|  | (-3.6244) | (-2.4725) | (-3.1649) | (-1.8747) | (-2.2155) | (-0.9121) |
| 19.province | -13.6354^***^ | -9.5414^***^ | -11.5683^***^ | -28.0375 | -23.0761 | -11.6595 |
|  | (-3.4043) | (-2.7521) | (-2.9655) | (-0.7423) | (-0.8833) | (-0.7869) |
| 20.province | -13.5091^***^ | -8.0523^***^ | -12.1984^***^ | -30.4340^**^ | -22.1675^*^ | -12.8256 |
|  | (-3.7018) | (-2.8611) | (-3.0285) | (-2.0608) | (-1.8007) | (-1.5492) |
| 21.province | -2.6812 | -1.4691 | -2.0627 | -4.3381 | -8.8526 | -0.3493 |
|  | (-1.2944) | (-0.7870) | (-1.5353) | (-0.3356) | (-0.8134) | (-0.0584) |
| 22.province | -10.0066^***^ | -10.2011^***^ | -4.8010^***^ | -43.0000^***^ | -37.2521^***^ | -17.0080^**^ |
|  | (-3.9611) | (-3.8844) | (-2.6375) | (-2.8889) | (-2.7994) | (-2.5499) |
| 23.province | -0.4319 | -2.8466 | 1.0475 | -28.0173^**^ | -23.1072^**^ | -14.2530^*^ |
|  | (-0.1429) | (-1.1656) | (0.5261) | (-2.1156) | (-2.2461) | (-1.9264) |
| 24.province | -5.7278^***^ | -4.1707^**^ | -2.9703^*^ | -30.5560^**^ | -24.0177^**^ | -13.9532^**^ |
|  | (-2.8549) | (-2.5157) | (-1.9228) | (-2.4546) | (-2.3103) | (-2.3768) |
| 25.province | -4.0222^*^ | -3.1402^*^ | -1.5215 | -3.3428 | -2.1060 | -5.6438 |
|  | (-1.7601) | (-1.6853) | (-0.9461) | (-0.2693) | (-0.2153) | (-0.8929) |
| 26.province | -9.2860^***^ | -7.9350^***^ | -4.1498^*^ | -39.1525^**^ | -28.0516^**^ | -14.5208^*^ |
|  | (-3.0084) | (-2.9052) | (-1.8590) | (-2.3928) | (-2.1940) | (-1.7746) |
| 27.province | -21.3315^***^ | -15.2788^***^ | -17.3583^***^ | -48.2028^*^ | -34.6488 | -31.4413^***^ |
|  | (-5.9438) | (-4.3967) | (-3.3556) | (-1.6632) | (-1.4017) | (-2.7584) |
| 28.province | -17.7907^***^ | -13.9902^***^ | -11.1019^***^ | -15.2437 | -14.0916 | -5.1109 |
|  | (-5.5373) | (-5.7847) | (-3.8078) | (-0.7242) | (-0.8876) | (-0.5218) |
| 29.province | -6.6930^**^ | -5.5933^*^ | -4.7058^**^ | -22.1972^*^ | -14.5956 | -15.7529^**^ |
|  | (-2.3577) | (-1.8776) | (-2.2518) | (-1.7691) | (-1.2856) | (-2.5595) |
| 30.province | -5.4289^***^ | -4.1142^***^ | -2.9762^**^ | -11.0419 | -8.4789 | -5.4934 |
|  | (-3.0098) | (-2.6279) | (-2.2866) | (-1.1170) | (-1.0782) | (-1.0934) |
| 31.province | -8.8009^**^ | -6.1876^**^ | -6.6721^**^ | -24.1412^*^ | -21.9193^*^ | -11.3592 |
|  | (-2.3846) | (-2.1051) | (-2.1545) | (-1.7463) | (-1.8111) | (-1.5181) |
| _cons | -1.0e+02^***^ | -82.9563^***^ | -66.6406^***^ | -5.0e+02^***^ | -4.2e+02^***^ | -2.0e+02^***^ |
|  | (-5.5947) | (-6.6445) | (-3.6603) | (-3.7568) | (-3.3998) | (-4.0078) |
| / |  |  |  |  |  |  |
| var(e.PAT) | 210.0118^***^ |  |  | 8.2e+03^***^ |  |  |
|  | (5.3936) |  |  | (3.0559) |  |  |
| var(e.INPAT) |  | 126.4368^***^ |  |  | 4.9e+03^**^ |  |
|  |  | (5.3261) |  |  | (2.4707) |  |
| var(e.UPAT) |  |  | 105.7288^***^ |  |  | 1.5e+03^***^ |
|  |  |  | (2.6866) |  |  | (3.2819) |
| *N* | 12397 | 12397 | 12397 | 10384 | 10384 | 10384 |
| pseudo *R*^2^ | 0.0367 | 0.0383 | 0.0436 | 0.0249 | 0.0262 | 0.0369 |

*t* statistics in parentheses

^*^ *p* < 0.1, ^**^ *p* < 0.05, ^***^ *p* < 0.01

**Table 8** Sample results of high-tech/non-high-tech industries.

|  | (1) | (2) | (3) | (4) | (5) | (6) |
| --- | --- | --- | --- | --- | --- | --- |
|  | PAT | INPAT | UPAT | PAT | INPAT | UPAT |
| main |  |  |  |  |  |  |
| DIF | -0.0876 | -0.0003 | -0.0544 | 0.3459^***^ | 0.3703^***^ | 0.1219^***^ |
|  | (-0.9011) | (-0.0034) | (-1.0374) | (4.1251) | (3.6239) | (5.3596) |
| Size | 23.7073^***^ | 19.4858^***^ | 10.5268^***^ | 14.4033^***^ | 12.4366^***^ | 5.5911^***^ |
|  | (4.1043) | (3.4923) | (4.4695) | (6.2159) | (4.8496) | (8.9732) |
| Age | -39.3452^***^ | -25.5631^***^ | -20.4796^***^ | -2.9089 | -2.7036 | -1.1554 |
|  | (-3.2000) | (-2.9909) | (-3.2312) | (-1.1072) | (-1.0695) | (-1.0105) |
| lev | -6.0308 | -13.5415 | 7.0397 | 8.6734^**^ | 2.5669 | 8.8329^***^ |
|  | (-0.4383) | (-1.1018) | (1.1844) | (2.0414) | (0.6386) | (4.9294) |
| Top1 | 14.6191 | -6.7778 | 22.5027^**^ | -0.4877 | -0.6287 | 2.3432 |
|  | (0.6375) | (-0.3524) | (2.1570) | (-0.0406) | (-0.0572) | (0.6576) |
| Mfee | -18.0284^**^ | -9.4823 | -9.7575^*^ | 16.3812^**^ | 20.4568^**^ | 0.8404 |
|  | (-1.9620) | (-1.4182) | (-1.9430) | (1.9966) | (2.5051) | (0.2293) |
| Growth | 0.4578 | 0.9515 | -0.4515 | 1.6655 | 1.4448 | 0.3400 |
|  | (0.3122) | (0.7703) | (-0.5577) | (1.1043) | (1.0030) | (0.6276) |
| ROE | 3.0983 | 2.3252 | 4.4603 | 11.4732^***^ | 9.7829^**^ | 4.6374^**^ |
|  | (0.6007) | (0.5196) | (1.5941) | (2.7751) | (2.5310) | (2.4933) |
| TobinQ | 5.0518 | 3.3271 | 1.0670 | -0.4608 | -0.7668 | 0.1074 |
|  | (0.9314) | (0.8929) | (0.2769) | (-0.4220) | (-0.8148) | (0.1832) |
| Mshare | 23.5052 | 24.2027 | 3.2560 | -15.8798 | -9.9443 | -3.4013 |
|  | (0.6885) | (0.9156) | (0.1510) | (-1.2148) | (-0.9363) | (-0.5355) |
| Indep | 104.0517^**^ | 82.4691^**^ | 48.1022^*^ | -28.2267 | -28.1049^*^ | -7.7544 |
|  | (2.2204) | (2.3941) | (1.8738) | (-1.5457) | (-1.7447) | (-1.0201) |
| Dual | 8.1676 | 8.3521^*^ | 1.8042 | 1.0159 | 0.9053 | 0.3481 |
|  | (1.4934) | (1.9400) | (0.7014) | (0.7049) | (0.7439) | (0.4884) |
| SOE | 6.2976 | 5.6911 | 2.2408 | 3.6678 | 5.6583^*^ | -0.8406 |
|  | (1.4319) | (1.5252) | (1.0558) | (1.2719) | (1.8724) | (-0.9999) |
| Board | 8.3040 | 15.2888 | -2.5389 | -0.3519 | 0.5164 | -1.3766 |
|  | (0.6063) | (1.1835) | (-0.4361) | (-0.0463) | (0.0746) | (-0.5463) |
| 2011.year | 0.0000 | 0.0000 | 0.0000 | 0.0000 | 0.0000 | 0.0000 |
|  | (.) | (.) | (.) | (.) | (.) | (.) |
| 2012.year | 14.1068^**^ | 9.0673^*^ | 7.9701^**^ | -14.2842^***^ | -14.2425^***^ | -5.2051^***^ |
|  | (2.3893) | (1.8147) | (2.5127) | (-3.8041) | (-3.3308) | (-4.5872) |
| 2013.year | 21.4968^**^ | 13.6395^*^ | 10.4874^**^ | -28.2352^***^ | -29.1644^***^ | -10.0104^***^ |
|  | (2.1906) | (1.6553) | (2.0244) | (-4.0379) | (-3.5141) | (-4.9901) |
| 2014.year | 24.1684^**^ | 12.9064 | 13.0551^**^ | -29.7174^***^ | -30.8583^***^ | -10.9917^***^ |
|  | (2.0834) | (1.3776) | (2.0956) | (-3.9393) | (-3.4668) | (-4.8165) |
| 2015.year | 37.0000^**^ | 18.7736 | 21.3121^***^ | -40.8873^***^ | -43.0070^***^ | -14.4677^***^ |
|  | (2.4763) | (1.5704) | (2.6026) | (-4.0439) | (-3.5693) | (-4.8982) |
| 2016.year | 46.2168^***^ | 24.5265^*^ | 25.6585^***^ | -45.0284^***^ | -48.2464^***^ | -16.2832^***^ |
|  | (2.5961) | (1.7469) | (2.6747) | (-3.8861) | (-3.4587) | (-4.8260) |
| 2017.year | 58.5925^***^ | 33.7968^**^ | 31.2418^***^ | -52.9822^***^ | -57.7222^***^ | -18.7110^***^ |
|  | (2.7299) | (1.9709) | (2.7388) | (-3.8503) | (-3.4750) | (-4.6600) |
| 2018.year | 70.2483^***^ | 39.8041^**^ | 35.8696^***^ | -55.1348^***^ | -59.9538^***^ | -19.7172^***^ |
|  | (3.0099) | (2.1572) | (2.9143) | (-3.8504) | (-3.4887) | (-4.5054) |
| 2019.year | 78.3509^***^ | 42.0458^**^ | 43.0408^***^ | -59.6809^***^ | -66.9647^***^ | -20.4141^***^ |
|  | (3.1373) | (2.1535) | (3.1861) | (-3.8328) | (-3.5469) | (-4.3080) |
| 2020.year | 57.3811^**^ | 35.6591^*^ | 21.3805^*^ | -75.1821^***^ | -75.1989^***^ | -31.9047^***^ |
|  | (2.3183) | (1.7752) | (1.6913) | (-4.2887) | (-3.6228) | (-6.0864) |
| 2021.year | -32.5922 | -31.9838 | -21.7624 | -1.1e+02^***^ | -1.1e+02^***^ | -46.4538^***^ |
|  | (-1.2274) | (-1.4134) | (-1.4506) | (-4.8750) | (-3.9447) | (-7.3558) |
| 1.province | 0.0000 | 0.0000 | 0.0000 | 0.0000 | 0.0000 | 0.0000 |
|  | (.) | (.) | (.) | (.) | (.) | (.) |
| 2.province | 29.1487^*^ | 19.8281 | 11.5716^*^ | -15.6461^***^ | -14.0364^**^ | -6.9799^***^ |
|  | (1.8728) | (1.5673) | (1.8084) | (-2.6921) | (-2.4782) | (-3.2717) |
| 3.province | -17.0999 | -15.5657^*^ | -4.1545 | -9.5433^*^ | -9.9569^*^ | -4.0760^*^ |
|  | (-1.5621) | (-1.6594) | (-0.7499) | (-1.8344) | (-1.9453) | (-1.8479) |
| 4.province | -11.8092 | -11.1124 | 0.0767 | -1.9352 | 0.7723 | 2.6581 |
|  | (-0.8371) | (-0.8973) | (0.0111) | (-0.2926) | (0.1224) | (0.9100) |
| 5.province | -0.3011 | -1.3855 | -0.7524 | -12.9358^***^ | -11.7898^***^ | -4.8294^***^ |
|  | (-0.0279) | (-0.1569) | (-0.1416) | (-3.1619) | (-3.1169) | (-2.7385) |
| 6.province | -24.6208^**^ | -13.3956 | -14.2976^**^ | -34.7233^***^ | -21.3400^***^ | -21.3209^***^ |
|  | (-2.1160) | (-1.3607) | (-2.3179) | (-4.7826) | (-3.2818) | (-3.5514) |
| 7.province | -15.5294 | -6.4779 | -11.3503 | -14.4354^**^ | -9.4643^*^ | -7.0601^***^ |
|  | (-0.9799) | (-0.4765) | (-1.2162) | (-2.3052) | (-1.6466) | (-2.6246) |
| 8.province | -14.2369 | -12.0909 | -9.0315 | -28.8291^**^ | -27.5421^**^ | -12.6903^**^ |
|  | (-1.1211) | (-1.0953) | (-1.4262) | (-2.1736) | (-2.3146) | (-2.5478) |
| 9.province | -22.9359^*^ | -17.6399^*^ | -7.7617 | -2.9808 | -0.7050 | -0.2453 |
|  | (-1.8394) | (-1.7297) | (-1.2123) | (-0.5612) | (-0.1412) | (-0.1064) |
| 10.province | -4.0408 | -10.1482 | -2.1784 | -4.2698 | -2.6064 | -2.6824 |
|  | (-0.4314) | (-1.1179) | (-0.4407) | (-0.5480) | (-0.3493) | (-1.1765) |
| 11.province | -16.7338 | -12.9676 | -7.4723 | -19.0201^***^ | -14.9678^**^ | -6.6386^**^ |
|  | (-1.0425) | (-1.0172) | (-0.8388) | (-2.6337) | (-2.2353) | (-2.0504) |
| 12.province | 0.6080 | 1.1980 | 2.9870 | -9.1921^*^ | -6.2625 | -3.3040 |
|  | (0.0578) | (0.1379) | (0.5408) | (-1.9247) | (-1.4746) | (-1.5087) |
| 13.province | -20.9938^*^ | -13.2748 | -11.1066^*^ | -7.8997^*^ | -6.0547 | -3.4108 |
|  | (-1.8504) | (-1.4287) | (-1.7247) | (-1.6873) | (-1.5582) | (-1.4585) |
| 14.province | -31.0457^**^ | -24.2974^**^ | -16.2463^**^ | -21.9106^***^ | -14.6539^**^ | -8.9002^**^ |
|  | (-2.1349) | (-1.9663) | (-2.1740) | (-2.9496) | (-2.3302) | (-2.5440) |
| 15.province | -0.8587 | 0.7650 | 0.4176 | -11.8961^**^ | -9.7899^**^ | -5.7442^***^ |
|  | (-0.0955) | (0.1026) | (0.0904) | (-2.5014) | (-2.4294) | (-3.1496) |
| 16.province | -4.1240 | -6.6661 | 0.0704 | -15.2928^**^ | -15.1258^***^ | -4.6074^*^ |
|  | (-0.3484) | (-0.6037) | (0.0117) | (-2.5151) | (-2.7907) | (-1.8401) |
| 17.province | -26.2595^**^ | -17.5718 | -13.0881^**^ | -11.4470^*^ | -11.7383^**^ | -2.5762 |
|  | (-1.9914) | (-1.4831) | (-2.1049) | (-1.9076) | (-2.3081) | (-0.9547) |
| 18.province | -11.6724 | -9.7455 | -1.6200 | -19.6928^*^ | -15.6942^**^ | -3.3137 |
|  | (-1.0569) | (-1.1393) | (-0.2865) | (-1.9257) | (-2.0474) | (-0.6763) |
| 19.province | -23.8810 | -15.1573 | -16.8964 | -45.8332^***^ | -29.9397^**^ | -72.8886^***^ |
|  | (-1.2262) | (-0.9426) | (-1.5747) | (-3.1635) | (-2.4300) | (-12.0734) |
| 20.province | -22.3736^*^ | -9.2591 | -10.6784 |  |  |  |
|  | (-1.7217) | (-0.8920) | (-1.3109) |  |  |  |
| 21.province | 1.1493 | 0.0280 | 0.6752 | -4.5619 | -4.3071 | -1.7550 |
|  | (0.1144) | (0.0034) | (0.1319) | (-0.9651) | (-1.0385) | (-0.8555) |
| 22.province | -24.0087^**^ | -26.1460^**^ | -7.7153 | -25.1849^***^ | -24.9803^***^ | -8.9529^***^ |
|  | (-1.9969) | (-2.2311) | (-1.4014) | (-4.5425) | (-4.2043) | (-3.8766) |
| 23.province | 0.9800 | -7.2865 | 0.3986 | -11.9373^*^ | -15.1948^***^ | -2.9557 |
|  | (0.0913) | (-0.8166) | (0.0702) | (-1.7867) | (-2.6649) | (-0.9131) |
| 24.province | -11.3857 | -9.6893 | -5.0807 | -22.2739^***^ | -20.7884^***^ | -8.1711^***^ |
|  | (-1.0692) | (-1.0862) | (-0.9557) | (-3.8269) | (-3.5256) | (-3.5274) |
| 25.province | 11.6249 | 11.0655 | 5.6243 | -7.0651 | -4.9621 | -4.6117^*^ |
|  | (1.2018) | (1.4199) | (1.0950) | (-1.1786) | (-0.8519) | (-1.8898) |
| 26.province | -23.6996 | -17.9379 | -5.9382 | -23.1758^***^ | -21.0025^***^ | -8.1184^***^ |
|  | (-1.5643) | (-1.3862) | (-0.7939) | (-3.8909) | (-3.7715) | (-3.1536) |
| 27.province | -53.3676^**^ | -32.5442 | -38.9985^***^ | -35.0363^***^ | -38.2518^***^ | -8.1138^***^ |
|  | (-2.0968) | (-1.5816) | (-2.7515) | (-5.5124) | (-4.8113) | (-3.9925) |
| 28.province | -35.5744^***^ | -26.6483^**^ | -15.2207^**^ | 41.8516 | 22.7977 | 19.3557 |
|  | (-2.8574) | (-2.4459) | (-2.4627) | (0.9596) | (0.8639) | (1.0855) |
| 29.province | -7.3455 | -5.7136 | -3.0184 | -15.8492 | -8.7332 | -14.8750^***^ |
|  | (-0.7048) | (-0.6084) | (-0.5594) | (-1.6004) | (-0.8461) | (-3.6813) |
| 30.province | -1.0613 | 0.1551 | -0.1924 | -18.9900^***^ | -17.6199^***^ | -7.2684^***^ |
|  | (-0.1228) | (0.0218) | (-0.0424) | (-4.3950) | (-4.1948) | (-3.9755) |
| 31.province | -7.3863 | -5.5026 | -4.9986 | -6.6896 | -5.3881 | -1.5095 |
|  | (-0.6210) | (-0.5465) | (-0.7662) | (-0.4371) | (-0.3697) | (-0.2927) |
| _cons | -5.5e+02^***^ | -5.0e+02^***^ | -2.3e+02^***^ | -3.3e+02^***^ | -3.0e+02^***^ | -1.3e+02^***^ |
|  | (-4.3192) | (-3.5897) | (-5.0621) | (-5.2154) | (-4.2202) | (-7.9092) |
| / |  |  |  |  |  |  |
| var(e.PAT) | 7.9e+03^***^ |  |  | 1.2e+03^***^ |  |  |
|  | (2.7407) |  |  | (3.0176) |  |  |
| var(e.INPAT) |  | 4.8e+03^**^ |  |  | 924.0678^**^ |  |
|  |  | (2.1342) |  |  | (2.3806) |  |
| var(e.UPAT) |  |  | 1.7e+03^***^ |  |  | 171.8367^***^ |
|  |  |  | (3.1365) |  |  | (6.4882) |
| *N* | 13816 | 13816 | 13816 | 8965 | 8965 | 8965 |
| pseudo *R*^2^ | 0.0384 | 0.0432 | 0.0540 | 0.0411 | 0.0429 | 0.0662 |

*t* statistics in parentheses

^*^ *p* < 0.1, ^**^ *p* < 0.05, ^***^ *p* < 0.01

|  | (1) | (2) | (3) | (4) | (5) | (6) |
| --- | --- | --- | --- | --- | --- | --- |
|  | PAT | INPAT | UPAT | PAT | INPAT | UPAT |
| main |  |  |  |  |  |  |
| DIFB | -0.0567 | 0.0058 | -0.0351 | 0.2442^***^ | 0.2563^***^ | 0.0906^***^ |
|  | (-0.7652) | (0.0929) | (-0.8731) | (4.2962) | (3.7491) | (5.3653) |
| Size | 23.6947^***^ | 19.4837^***^ | 10.5184^***^ | 14.3899^***^ | 12.4187^***^ | 5.5851^***^ |
|  | (4.1052) | (3.4937) | (4.4687) | (6.2136) | (4.8466) | (8.9840) |
| Age | -39.3385^***^ | -25.5701^***^ | -20.4729^***^ | -2.8073 | -2.6100 | -1.1136 |
|  | (-3.2004) | (-2.9913) | (-3.2312) | (-1.0714) | (-1.0360) | (-0.9750) |
| lev | -6.0428 | -13.5872 | 7.0337 | 8.6099^**^ | 2.5356 | 8.7847^***^ |
|  | (-0.4369) | (-1.1001) | (1.1786) | (2.0306) | (0.6324) | (4.9144) |
| Top1 | 14.6742 | -6.8257 | 22.5347^**^ | -0.6931 | -0.8588 | 2.2549 |
|  | (0.6380) | (-0.3537) | (2.1558) | (-0.0575) | (-0.0779) | (0.6301) |
| Mfee | -18.0577^**^ | -9.4861 | -9.7800^*^ | 16.1026^**^ | 20.2266^**^ | 0.6562 |
|  | (-1.9628) | (-1.4182) | (-1.9435) | (1.9893) | (2.5148) | (0.1795) |
| Growth | 0.4629 | 0.9483 | -0.4481 | 1.6572 | 1.4391 | 0.3366 |
|  | (0.3161) | (0.7685) | (-0.5550) | (1.0942) | (0.9952) | (0.6205) |
| ROE | 3.1643 | 2.3214 | 4.5069 | 11.0523^***^ | 9.3573^**^ | 4.4857^**^ |
|  | (0.6146) | (0.5191) | (1.6118) | (2.7095) | (2.4701) | (2.4263) |
| TobinQ | 5.0633 | 3.3309 | 1.0777 | -0.4090 | -0.6954 | 0.1303 |
|  | (0.9337) | (0.8942) | (0.2799) | (-0.3751) | (-0.7441) | (0.2229) |
| Mshare | 23.3318 | 24.2592 | 3.1713 | -14.9185 | -9.0423 | -3.0885 |
|  | (0.6829) | (0.9156) | (0.1470) | (-1.1348) | (-0.8457) | (-0.4839) |
| Indep | 104.0488^**^ | 82.4883^**^ | 48.1014^*^ | -28.5088 | -28.2678^*^ | -7.8306 |
|  | (2.2205) | (2.3943) | (1.8738) | (-1.5622) | (-1.7564) | (-1.0309) |
| Dual | 8.1659 | 8.3466^*^ | 1.8044 | 0.9949 | 0.8892 | 0.3376 |
|  | (1.4950) | (1.9410) | (0.7022) | (0.6909) | (0.7314) | (0.4741) |
| SOE | 6.2895 | 5.6889 | 2.2341 | 3.7181 | 5.7296^*^ | -0.8229 |
|  | (1.4308) | (1.5252) | (1.0531) | (1.2876) | (1.8880) | (-0.9785) |
| Board | 8.3178 | 15.2911 | -2.5303 | -0.2951 | 0.5510 | -1.3230 |
|  | (0.6071) | (1.1835) | (-0.4346) | (-0.0389) | (0.0797) | (-0.5262) |
| 2011.year | 0.0000 | 0.0000 | 0.0000 | 0.0000 | 0.0000 | 0.0000 |
|  | (.) | (.) | (.) | (.) | (.) | (.) |
| 2012.year | 12.5231^***^ | 8.8015^**^ | 6.9867^***^ | -8.8247^***^ | -8.1950^***^ | -3.4813^***^ |
|  | (2.6005) | (2.1544) | (2.6762) | (-3.6200) | (-3.1264) | (-4.0451) |
| 2013.year | 17.9988^**^ | 13.1955^**^ | 8.3121^**^ | -15.8786^***^ | -15.5419^***^ | -5.9841^***^ |
|  | (2.5649) | (2.2374) | (2.2525) | (-4.0515) | (-3.4870) | (-4.6296) |
| 2014.year | 21.3573^**^ | 12.2674 | 11.3051^**^ | -20.8601^***^ | -20.8265^***^ | -8.3483^***^ |
|  | (2.1784) | (1.5759) | (2.1383) | (-3.9576) | (-3.4741) | (-4.6343) |
| 2015.year | 32.7199^***^ | 18.0123^*^ | 18.6489^***^ | -26.6442^***^ | -27.1142^***^ | -10.0119^***^ |
|  | (2.7684) | (1.9524) | (2.8320) | (-4.1139) | (-3.6323) | (-4.6808) |
| 2016.year | 40.8112^***^ | 23.6879^**^ | 22.2929^***^ | -26.7150^***^ | -27.9340^***^ | -10.4529^***^ |
|  | (2.9624) | (2.2460) | (2.9803) | (-3.8298) | (-3.4268) | (-4.5261) |
| 2017.year | 51.6711^***^ | 32.8443^***^ | 26.9342^***^ | -28.9075^***^ | -31.1168^***^ | -10.9405^***^ |
|  | (3.2134) | (2.6222) | (3.1387) | (-3.7447) | (-3.4435) | (-4.2279) |
| 2018.year | 63.1845^***^ | 38.7398^***^ | 31.4704^***^ | -30.6760^***^ | -32.8269^***^ | -11.8952^***^ |
|  | (3.5135) | (2.8007) | (3.3129) | (-3.7322) | (-3.4625) | (-4.0559) |
| 2019.year | 71.0869^***^ | 40.8695^***^ | 38.5185^***^ | -34.6875^***^ | -39.1415^***^ | -12.4986^***^ |
|  | (3.6248) | (2.7585) | (3.5927) | (-3.7236) | (-3.5793) | (-3.8262) |
| 2020.year | 49.9410^***^ | 34.3985^**^ | 16.7409^*^ | -49.7138^***^ | -46.8026^***^ | -23.8811^***^ |
|  | (2.5815) | (2.2433) | (1.7217) | (-4.5141) | (-3.7295) | (-6.3088) |
| 2021.year | -40.1790^*^ | -33.4893^*^ | -26.4956^**^ | -83.9971^***^ | -77.5345^***^ | -38.8005^***^ |
|  | (-1.7725) | (-1.7792) | (-2.0001) | (-5.3162) | (-4.1993) | (-7.7175) |
| 1.province | 0.0000 | 0.0000 | 0.0000 | 0.0000 | 0.0000 | 0.0000 |
|  | (.) | (.) | (.) | (.) | (.) | (.) |
| 2.province | 29.0192^*^ | 19.5571 | 11.4955^*^ | -15.8639^***^ | -14.0605^**^ | -7.2489^***^ |
|  | (1.8609) | (1.5571) | (1.7715) | (-2.7474) | (-2.5125) | (-3.3782) |
| 3.province | -17.0758 | -15.7342^*^ | -4.1345 | -10.1796^*^ | -10.5070^**^ | -4.4158^**^ |
|  | (-1.5434) | (-1.6552) | (-0.7403) | (-1.9443) | (-2.0341) | (-1.9970) |
| 4.province | -10.6857 | -11.1303 | 0.7684 | -6.2858 | -3.9607 | 1.1480 |
|  | (-0.7575) | (-0.8974) | (0.1110) | (-0.9858) | (-0.6685) | (0.4028) |
| 5.province | -0.1703 | -1.6161 | -0.6642 | -14.0803^***^ | -12.8756^***^ | -5.4177^***^ |
|  | (-0.0158) | (-0.1818) | (-0.1244) | (-3.3514) | (-3.2813) | (-3.0054) |
| 6.province | -24.0434^**^ | -13.3607 | -13.9399^**^ | -35.4340^***^ | -22.2019^***^ | -21.5200^***^ |
|  | (-2.0756) | (-1.3606) | (-2.2728) | (-4.8688) | (-3.4010) | (-3.6218) |
| 7.province | -14.5036 | -6.4773 | -10.7195 | -17.3493^***^ | -12.5821^**^ | -8.1287^***^ |
|  | (-0.9186) | (-0.4772) | (-1.1550) | (-2.6842) | (-2.0954) | (-3.0100) |
| 8.province | -13.5610 | -12.2536 | -8.6130 | -32.1451^**^ | -30.8863^**^ | -13.9624^***^ |
|  | (-1.0526) | (-1.0924) | (-1.3428) | (-2.3902) | (-2.5200) | (-2.7879) |
| 9.province | -22.2002^*^ | -17.5540^*^ | -7.3062 | -5.1465 | -3.1750 | -0.8868 |
|  | (-1.7980) | (-1.7360) | (-1.1499) | (-0.9763) | (-0.6474) | (-0.3857) |
| 10.province | -3.3469 | -10.1666 | -1.7471 | -6.8493 | -5.4092 | -3.5894 |
|  | (-0.3561) | (-1.1144) | (-0.3529) | (-0.8665) | (-0.7091) | (-1.5745) |
| 11.province | -16.0517 | -12.9280 | -7.0360 | -20.9487^***^ | -17.1264^**^ | -7.2260^**^ |
|  | (-1.0040) | (-1.0163) | (-0.7934) | (-2.8941) | (-2.5418) | (-2.2558) |
| 12.province | 0.7646 | 1.0996 | 3.0860 | -9.8508^**^ | -6.9676 | -3.5623 |
|  | (0.0720) | (0.1255) | (0.5531) | (-2.0559) | (-1.6340) | (-1.6233) |
| 13.province | -20.6984^*^ | -13.3091 | -10.9217^*^ | -8.6872^*^ | -6.9464^*^ | -3.6850 |
|  | (-1.8257) | (-1.4292) | (-1.6988) | (-1.8354) | (-1.7543) | (-1.5720) |
| 14.province | -30.1958^**^ | -24.2474^**^ | -15.7135^**^ | -24.7387^***^ | -17.8690^***^ | -9.8091^***^ |
|  | (-2.0898) | (-1.9726) | (-2.1167) | (-3.3030) | (-2.7440) | (-2.8575) |
| 15.province | -0.8788 | 0.5794 | 0.4072 | -12.3227^**^ | -10.1323^**^ | -6.0106^***^ |
|  | (-0.0966) | (0.0768) | (0.0871) | (-2.5582) | (-2.4824) | (-3.2657) |
| 16.province | -3.9732 | -6.6688 | 0.1598 | -15.3680^**^ | -15.3346^***^ | -4.5448^*^ |
|  | (-0.3356) | (-0.6043) | (0.0266) | (-2.5247) | (-2.8119) | (-1.8092) |
| 17.province | -25.7030^*^ | -17.5949 | -12.7433^**^ | -13.2683^**^ | -13.6548^***^ | -3.1906 |
|  | (-1.9586) | (-1.4859) | (-2.0599) | (-2.1987) | (-2.6239) | (-1.1821) |
| 18.province | -10.6943 | -9.7274 | -1.0089 | -24.3400^**^ | -20.8613^**^ | -4.9344 |
|  | (-0.9734) | (-1.1414) | (-0.1789) | (-2.2991) | (-2.5127) | (-1.0102) |
| 19.province | -22.4294 | -15.1375 | -15.9799 | -49.9996^***^ | -34.3517^***^ | -77.0504^***^ |
|  | (-1.1578) | (-0.9425) | (-1.4987) | (-3.4891) | (-2.8203) | (-12.3120) |
| 20.province | -21.0722^*^ | -9.1585 | -9.8897 |  |  |  |
|  | (-1.6506) | (-0.8919) | (-1.2247) |  |  |  |
| 21.province | 1.5664 | -0.0040 | 0.9334 | -6.0043 | -5.8819 | -2.2537 |
|  | (0.1557) | (-0.0005) | (0.1823) | (-1.2625) | (-1.3943) | (-1.0978) |
| 22.province | -23.0653^*^ | -26.1544^**^ | -7.1418 | -29.1117^***^ | -29.0928^***^ | -10.3083^***^ |
|  | (-1.9254) | (-2.2370) | (-1.3015) | (-4.8742) | (-4.3381) | (-4.4378) |
| 23.province | 1.6693 | -7.3693 | 0.8291 | -14.6793^**^ | -18.0999^***^ | -3.9788 |
|  | (0.1541) | (-0.8157) | (0.1447) | (-2.1313) | (-2.9596) | (-1.2156) |
| 24.province | -12.1150 | -9.9366 | -5.5259 | -20.2553^***^ | -18.3837^***^ | -7.6582^***^ |
|  | (-1.1339) | (-1.1087) | (-1.0484) | (-3.7296) | (-3.4583) | (-3.3814) |
| 25.province | 12.0511 | 11.0857 | 5.8864 | -8.1395 | -6.1408 | -4.9907^**^ |
|  | (1.2435) | (1.4212) | (1.1450) | (-1.3544) | (-1.0520) | (-2.0315) |
| 26.province | -23.4785 | -17.9836 | -5.8009 | -23.9680^***^ | -21.8712^***^ | -8.3960^***^ |
|  | (-1.5476) | (-1.3867) | (-0.7745) | (-3.9883) | (-3.8477) | (-3.2665) |
| 27.province | -52.5860^**^ | -32.5874 | -38.5023^***^ | -40.4176^***^ | -44.2960^***^ | -9.9898^***^ |
|  | (-2.0692) | (-1.5818) | (-2.7264) | (-5.5975) | (-4.7294) | (-5.0593) |
| 28.province | -34.4602^***^ | -26.7146^**^ | -14.5311^**^ | 37.5321 | 18.1559 | 17.8028 |
|  | (-2.7771) | (-2.4385) | (-2.3632) | (0.8605) | (0.6844) | (1.0019) |
| 29.province | -6.6733 | -5.7386 | -2.6090 | -17.9259^*^ | -10.9777 | -15.5660^***^ |
|  | (-0.6384) | (-0.6062) | (-0.4821) | (-1.7692) | (-1.0447) | (-3.7213) |
| 30.province | -1.5434 | -0.0633 | -0.4861 | -17.8997^***^ | -16.2653^***^ | -7.0090^***^ |
|  | (-0.1788) | (-0.0089) | (-0.1076) | (-4.3101) | (-4.1619) | (-3.8843) |
| 31.province | -6.9078 | -5.4770 | -4.7062 | -8.4168 | -7.3905 | -2.0205 |
|  | (-0.5811) | (-0.5441) | (-0.7224) | (-0.5528) | (-0.5089) | (-0.3941) |
| _cons | -5.5e+02^***^ | -5.0e+02^***^ | -2.4e+02^***^ | -3.2e+02^***^ | -2.9e+02^***^ | -1.2e+02^***^ |
|  | (-4.3131) | (-3.5830) | (-5.0609) | (-5.2266) | (-4.2327) | (-7.8258) |
| / |  |  |  |  |  |  |
| var(e.PAT) | 7.9e+03^***^ |  |  | 1.2e+03^***^ |  |  |
|  | (2.7404) |  |  | (3.0113) |  |  |
| var(e.INPAT) |  | 4.8e+03^**^ |  |  | 925.4346^**^ |  |
|  |  | (2.1342) |  |  | (2.3748) |  |
| var(e.UPAT) |  |  | 1.7e+03^***^ |  |  | 171.6377^***^ |
|  |  |  | (3.1364) |  |  | (6.5071) |
| *N* | 13816 | 13816 | 13816 | 8965 | 8965 | 8965 |
| pseudo *R*^2^ | 0.0384 | 0.0432 | 0.0540 | 0.0411 | 0.0427 | 0.0665 |

*t* statistics in parentheses

^*^ *p* < 0.1, ^**^ *p* < 0.05, ^***^ *p* < 0.01

|  | (1) | (2) | (3) | (4) | (5) | (6) |
| --- | --- | --- | --- | --- | --- | --- |
|  | PAT | INPAT | UPAT | PAT | INPAT | UPAT |
| main |  |  |  |  |  |  |
| DIFD | -0.0851 | 0.0059 | -0.0524 | 0.2707^***^ | 0.2973^***^ | 0.0860^***^ |
|  | (-0.8775) | (0.0726) | (-1.0321) | (3.5257) | (3.2705) | (4.0548) |
| Size | 23.7077^***^ | 19.4836^***^ | 10.5268^***^ | 14.4642^***^ | 12.4786^***^ | 5.6176^***^ |
|  | (4.1033) | (3.4906) | (4.4712) | (6.1865) | (4.8284) | (8.9532) |
| Age | -39.3883^***^ | -25.5654^***^ | -20.5110^***^ | -3.2923 | -3.0874 | -1.2682 |
|  | (-3.2004) | (-2.9896) | (-3.2330) | (-1.2271) | (-1.1873) | (-1.1042) |
| lev | -6.2652 | -13.5586 | 6.8929 | 9.0025^**^ | 2.9718 | 8.9842^***^ |
|  | (-0.4563) | (-1.1056) | (1.1642) | (2.1130) | (0.7429) | (4.9802) |
| Top1 | 14.3338 | -6.7879 | 22.3361^**^ | -0.1032 | -0.3616 | 2.4344 |
|  | (0.6258) | (-0.3531) | (2.1466) | (-0.0087) | (-0.0333) | (0.6889) |
| Mfee | -18.0099^**^ | -9.4878 | -9.7530^*^ | 17.5382^**^ | 21.4828^**^ | 1.4363 |
|  | (-1.9631) | (-1.4189) | (-1.9465) | (2.0876) | (2.5441) | (0.3959) |
| Growth | 0.4332 | 0.9510 | -0.4699 | 1.8288 | 1.6078 | 0.3925 |
|  | (0.2952) | (0.7704) | (-0.5774) | (1.1950) | (1.0935) | (0.7199) |
| ROE | 3.0453 | 2.3303 | 4.4344 | 11.6857^***^ | 10.0527^**^ | 4.7024^**^ |
|  | (0.5895) | (0.5201) | (1.5855) | (2.7634) | (2.5209) | (2.4905) |
| TobinQ | 5.0597 | 3.3301 | 1.0685 | -0.4881 | -0.7810 | 0.1052 |
|  | (0.9320) | (0.8933) | (0.2768) | (-0.4465) | (-0.8300) | (0.1810) |
| Mshare | 23.5300 | 24.2260 | 3.3121 | -16.9201 | -10.7515 | -3.8539 |
|  | (0.6874) | (0.9163) | (0.1531) | (-1.2815) | (-0.9972) | (-0.6048) |
| Indep | 104.1053^**^ | 82.4754^**^ | 48.1428^*^ | -28.2165 | -28.3954^*^ | -7.9229 |
|  | (2.2208) | (2.3942) | (1.8743) | (-1.5259) | (-1.7412) | (-1.0287) |
| Dual | 8.1377 | 8.3507^*^ | 1.7835 | 1.1065 | 1.0003 | 0.3839 |
|  | (1.4876) | (1.9397) | (0.6929) | (0.7623) | (0.8147) | (0.5350) |
| SOE | 6.2731 | 5.6914 | 2.2297 | 3.4546 | 5.4265^*^ | -0.9103 |
|  | (1.4274) | (1.5255) | (1.0512) | (1.2072) | (1.8209) | (-1.0801) |
| Board | 8.2804 | 15.2930 | -2.5422 | -0.6439 | 0.1588 | -1.5393 |
|  | (0.6050) | (1.1842) | (-0.4368) | (-0.0841) | (0.0228) | (-0.6056) |
| 2011.year | 0.0000 | 0.0000 | 0.0000 | 0.0000 | 0.0000 | 0.0000 |
|  | (.) | (.) | (.) | (.) | (.) | (.) |
| 2012.year | 14.0022^**^ | 8.7788^*^ | 7.8885^**^ | -11.0174^***^ | -11.0260^***^ | -3.6119^***^ |
|  | (2.3594) | (1.7215) | (2.5245) | (-3.1656) | (-2.8942) | (-3.3287) |
| 2013.year | 21.6472^**^ | 13.0765 | 10.5470^**^ | -22.9299^***^ | -24.1355^***^ | -7.3077^***^ |
|  | (2.1204) | (1.5063) | (2.0036) | (-3.4144) | (-3.1251) | (-3.6899) |
| 2014.year | 22.2787^**^ | 12.3986 | 11.8495^**^ | -17.0407^***^ | -17.8929^***^ | -5.7849^***^ |
|  | (2.2631) | (1.5361) | (2.2911) | (-3.1493) | (-2.9362) | (-3.2689) |
| 2015.year | 34.3513^***^ | 18.1308^*^ | 19.6322^***^ | -23.8555^***^ | -25.4883^***^ | -7.5299^***^ |
|  | (2.7507) | (1.7886) | (2.9368) | (-3.3330) | (-3.1205) | (-3.3552) |
| 2016.year | 45.4716^***^ | 23.6215^*^ | 25.1399^***^ | -32.4789^***^ | -35.8170^***^ | -10.5254^***^ |
|  | (2.6265) | (1.7038) | (2.7676) | (-3.1916) | (-3.0310) | (-3.4004) |
| 2017.year | 59.9621^**^ | 32.5587^*^ | 32.0115^***^ | -45.0275^***^ | -50.6711^***^ | -14.0339^***^ |
|  | (2.5709) | (1.7138) | (2.6572) | (-3.1990) | (-3.0812) | (-3.3160) |
| 2018.year | 69.8451^***^ | 38.6018^**^ | 35.5398^***^ | -40.5093^***^ | -45.7046^***^ | -12.7461^***^ |
|  | (2.9812) | (2.0508) | (2.9655) | (-3.1232) | (-3.0470) | (-3.0744) |
| 2019.year | 77.5101^***^ | 40.7821^**^ | 42.4349^***^ | -42.5731^***^ | -50.1346^***^ | -12.4737^***^ |
|  | (3.1525) | (2.0900) | (3.2763) | (-3.1011) | (-3.1206) | (-2.8334) |
| 2020.year | 56.3996^**^ | 34.3351^*^ | 20.6927^*^ | -56.8147^***^ | -57.1545^***^ | -23.4230^***^ |
|  | (2.3476) | (1.7285) | (1.7351) | (-3.6706) | (-3.2249) | (-4.9376) |
| 2021.year | -34.6214 | -33.4144 | -23.1098^*^ | -85.1099^***^ | -81.7570^***^ | -35.8948^***^ |
|  | (-1.3655) | (-1.5435) | (-1.6512) | (-4.4318) | (-3.6656) | (-6.6113) |
| 1.province | 0.0000 | 0.0000 | 0.0000 | 0.0000 | 0.0000 | 0.0000 |
|  | (.) | (.) | (.) | (.) | (.) | (.) |
| 2.province | 28.1531^*^ | 19.7005 | 10.9236^*^ | -11.2878^**^ | -9.5272^*^ | -5.2243^**^ |
|  | (1.8505) | (1.5726) | (1.7799) | (-2.1005) | (-1.9041) | (-2.5257) |
| 3.province | -17.9648^*^ | -15.6218^*^ | -4.7263 | -6.3599 | -6.5235 | -2.8815 |
|  | (-1.6590) | (-1.6872) | (-0.8643) | (-1.2221) | (-1.3018) | (-1.2956) |
| 4.province | -14.5641 | -10.8519 | -1.6291 | 4.5504 | 7.9550 | 4.5592 |
|  | (-0.9941) | (-0.8447) | (-0.2294) | (0.6063) | (1.1952) | (1.3749) |
| 5.province | -1.6046 | -1.4444 | -1.5994 | -7.9625^**^ | -6.4005^**^ | -2.9328^*^ |
|  | (-0.1516) | (-0.1685) | (-0.3066) | (-2.1236) | (-1.9988) | (-1.7237) |
| 6.province | -25.7691^**^ | -13.2495 | -15.0212^**^ | -33.6805^***^ | -20.0765^***^ | -21.1045^***^ |
|  | (-2.1651) | (-1.3222) | (-2.3858) | (-4.6966) | (-3.1523) | (-3.4624) |
| 7.province | -17.8218 | -6.2443 | -12.7703 | -8.3482 | -2.7089 | -5.1401^*^ |
|  | (-1.0922) | (-0.4480) | (-1.3324) | (-1.3337) | (-0.4711) | (-1.8533) |
| 8.province | -16.4765 | -11.9980 | -10.4246 | -21.5941 | -19.6924^*^ | -10.2951^**^ |
|  | (-1.2944) | (-1.0880) | (-1.6332) | (-1.6130) | (-1.6832) | (-2.0089) |
| 9.province | -23.9856^*^ | -17.4545^*^ | -8.4161 | -2.9716 | -0.4175 | -0.5045 |
|  | (-1.8754) | (-1.6705) | (-1.2891) | (-0.5556) | (-0.0831) | (-0.2163) |
| 10.province | -5.8015 | -9.9873 | -3.2682 | -0.0431 | 2.1769 | -1.4191 |
|  | (-0.6061) | (-1.0779) | (-0.6457) | (-0.0054) | (0.2899) | (-0.5923) |
| 11.province | -18.1836 | -12.7904 | -8.3750 | -16.6092^**^ | -12.2826^*^ | -6.0069^*^ |
|  | (-1.1132) | (-0.9858) | (-0.9254) | (-2.2719) | (-1.8750) | (-1.7812) |
| 12.province | -0.2821 | 1.2022 | 2.4223 | -7.3331 | -4.1221 | -2.6908 |
|  | (-0.0270) | (0.1393) | (0.4445) | (-1.5286) | (-0.9626) | (-1.2276) |
| 13.province | -21.6962^*^ | -13.2260 | -11.5630^*^ | -7.3553 | -5.2750 | -3.2654 |
|  | (-1.8943) | (-1.4166) | (-1.7794) | (-1.5796) | (-1.3730) | (-1.3886) |
| 14.province | -32.4907^**^ | -24.1030^*^ | -17.1617^**^ | -20.3278^***^ | -12.7209^*^ | -8.5993^**^ |
|  | (-2.1790) | (-1.9067) | (-2.2418) | (-2.5905) | (-1.9307) | (-2.2998) |
| 15.province | -1.6352 | 0.6901 | -0.0880 | -9.3728^*^ | -7.1586^*^ | -4.7075^***^ |
|  | (-0.1866) | (0.0949) | (-0.0195) | (-1.8949) | (-1.6637) | (-2.5993) |
| 16.province | -4.5670 | -6.6261 | -0.2168 | -16.7366^***^ | -16.4913^***^ | -5.2442^**^ |
|  | (-0.3863) | (-0.5990) | (-0.0361) | (-2.7212) | (-2.9475) | (-2.1091) |
| 17.province | -27.4394^**^ | -17.4676 | -13.8347^**^ | -9.3473 | -9.2321^*^ | -1.9493 |
|  | (-2.0329) | (-1.4547) | (-2.1724) | (-1.5936) | (-1.9126) | (-0.7259) |
| 18.province | -14.3247 | -9.4788 | -3.2665 | -13.3251 | -8.4648 | -1.4537 |
|  | (-1.2036) | (-1.0187) | (-0.5454) | (-1.3346) | (-1.1534) | (-0.2928) |
| 19.province | -26.4989 | -14.8669 | -18.5491^*^ | -38.3101^***^ | -21.6192^**^ | -70.3691^***^ |
|  | (-1.3176) | (-0.9011) | (-1.6872) | (-3.0282) | (-2.0980) | (-11.6995) |
| 20.province | -24.7054^*^ | -8.9353 | -12.1115 |  |  |  |
|  | (-1.7753) | (-0.8105) | (-1.4299) |  |  |  |
| 21.province | 0.1192 | 0.1073 | 0.0287 | -2.8396 | -2.3437 | -1.2678 |
|  | (0.0117) | (0.0127) | (0.0055) | (-0.5933) | (-0.5634) | (-0.6090) |
| 22.province | -26.2547^**^ | -25.9296^**^ | -9.1092 | -19.7823^***^ | -18.4801^***^ | -7.3876^***^ |
|  | (-2.0962) | (-2.1385) | (-1.5881) | (-3.7783) | (-3.5723) | (-3.0345) |
| 23.province | -1.0032 | -7.1571 | -0.8587 | -6.3273 | -9.0032^*^ | -1.1558 |
|  | (-0.0927) | (-0.7929) | (-0.1494) | (-0.9776) | (-1.7409) | (-0.3601) |
| 24.province | -11.0607 | -9.9358 | -4.9175 | -21.8654^***^ | -20.7168^***^ | -7.6313^***^ |
|  | (-1.0483) | (-1.1222) | (-0.9219) | (-3.6094) | (-3.3501) | (-3.2266) |
| 25.province | 10.8235 | 11.1653 | 5.1191 | -4.9525 | -2.6628 | -3.9444 |
|  | (1.1114) | (1.4195) | (0.9899) | (-0.8303) | (-0.4643) | (-1.6270) |
| 26.province | -24.3908 | -17.9066 | -6.3814 | -21.7435^***^ | -19.4543^***^ | -7.6395^***^ |
|  | (-1.6059) | (-1.3821) | (-0.8530) | (-3.6634) | (-3.5867) | (-2.9240) |
| 27.province | -55.5443^**^ | -32.3668 | -40.3538^***^ | -30.3503^***^ | -33.0602^***^ | -6.7507^***^ |
|  | (-2.1520) | (-1.5575) | (-2.8062) | (-5.3751) | (-4.9534) | (-3.1169) |
| 28.province | -38.1714^***^ | -26.4336^**^ | -16.8267^***^ | 48.7658 | 30.4514 | 21.4804 |
|  | (-2.8839) | (-2.3110) | (-2.5822) | (1.1056) | (1.1296) | (1.1894) |
| 29.province | -8.8685 | -5.5781 | -3.9743 | -12.8611 | -5.4690 | -13.8374^***^ |
|  | (-0.8301) | (-0.5874) | (-0.7208) | (-1.4463) | (-0.5873) | (-3.7629) |
| 30.province | -0.8687 | -0.0430 | -0.1053 | -19.0568^***^ | -17.8734^***^ | -7.0194^***^ |
|  | (-0.1001) | (-0.0060) | (-0.0230) | (-4.2267) | (-4.0305) | (-3.7500) |
| 31.province | -8.2653 | -5.3900 | -5.5498 | -5.3323 | -3.7746 | -1.1934 |
|  | (-0.6882) | (-0.5307) | (-0.8427) | (-0.3486) | (-0.2597) | (-0.2315) |
| _cons | -5.5e+02^***^ | -5.0e+02^***^ | -2.3e+02^***^ | -3.2e+02^***^ | -2.9e+02^***^ | -1.2e+02^***^ |
|  | (-4.3361) | (-3.6032) | (-5.0722) | (-5.1449) | (-4.1701) | (-7.8507) |
| / |  |  |  |  |  |  |
| var(e.PAT) | 7.9e+03^***^ |  |  | 1.2e+03^***^ |  |  |
|  | (2.7409) |  |  | (3.0185) |  |  |
| var(e.INPAT) |  | 4.8e+03^**^ |  |  | 930.9220^**^ |  |
|  |  | (2.1342) |  |  | (2.3806) |  |
| var(e.UPAT) |  |  | 1.7e+03^***^ |  |  | 173.1899^***^ |
|  |  |  | (3.1365) |  |  | (6.4925) |
| *N* | 13816 | 13816 | 13816 | 8965 | 8965 | 8965 |
| pseudo *R*^2^ | 0.0384 | 0.0432 | 0.0540 | 0.0399 | 0.0412 | 0.0647 |

*t* statistics in parentheses

^*^ *p* < 0.1, ^**^ *p* < 0.05, ^***^ *p* < 0.01

|  | (1) | (2) | (3) | (4) | (5) | (6) |
| --- | --- | --- | --- | --- | --- | --- |
|  | PAT | INPAT | UPAT | PAT | INPAT | UPAT |
| main |  |  |  |  |  |  |
| DIFL | -0.0392 | -0.0317 | -0.0245 | 0.1129^**^ | 0.1454^**^ | 0.0209 |
|  | (-0.8369) | (-0.7504) | (-1.0401) | (2.1781) | (2.4444) | (1.5637) |
| Size | 23.6807^***^ | 19.4949^***^ | 10.5109^***^ | 14.3828^***^ | 12.3586^***^ | 5.5953^***^ |
|  | (4.1043) | (3.4925) | (4.4734) | (6.1757) | (4.8234) | (8.8960) |
| Age | -39.3828^***^ | -25.5456^***^ | -20.4955^***^ | -3.4667 | -3.3159 | -1.3087 |
|  | (-3.1995) | (-2.9873) | (-3.2315) | (-1.2840) | (-1.2608) | (-1.1428) |
| lev | -6.4058 | -13.4873 | 6.8053 | 10.0952^**^ | 4.2585 | 9.3196^***^ |
|  | (-0.4653) | (-1.0999) | (1.1463) | (2.4102) | (1.1011) | (5.1419) |
| Top1 | 14.1702 | -6.8127 | 22.2310^**^ | -0.7100 | -1.1775 | 2.2648 |
|  | (0.6177) | (-0.3537) | (2.1371) | (-0.0593) | (-0.1073) | (0.6393) |
| Mfee | -18.0723^**^ | -9.4671 | -9.7833^*^ | 18.8707^**^ | 22.7259^***^ | 1.9477 |
|  | (-1.9656) | (-1.4175) | (-1.9481) | (2.2101) | (2.6071) | (0.5473) |
| Growth | 0.4178 | 0.9484 | -0.4822 | 1.9584 | 1.7658 | 0.4334 |
|  | (0.2847) | (0.7684) | (-0.5896) | (1.2558) | (1.1677) | (0.7874) |
| ROE | 3.0791 | 2.2648 | 4.4565 | 11.5556^***^ | 9.9743^**^ | 4.6512^**^ |
|  | (0.5951) | (0.5048) | (1.5859) | (2.7180) | (2.4706) | (2.4608) |
| TobinQ | 5.0820 | 3.3146 | 1.0944 | -0.4338 | -0.6878 | 0.1299 |
|  | (0.9374) | (0.8892) | (0.2846) | (-0.3985) | (-0.7328) | (0.2263) |
| Mshare | 24.1608 | 24.5686 | 3.8866 | -14.7270 | -8.5742 | -3.2205 |
|  | (0.6993) | (0.9254) | (0.1782) | (-1.1103) | (-0.7922) | (-0.5025) |
| Indep | 104.2992^**^ | 82.5444^**^ | 48.2831^*^ | -28.9000 | -29.1152^*^ | -8.2955 |
|  | (2.2226) | (2.3957) | (1.8762) | (-1.5380) | (-1.7536) | (-1.0620) |
| Dual | 8.1082 | 8.3528^*^ | 1.7685 | 1.0075 | 0.9125 | 0.3576 |
|  | (1.4826) | (1.9419) | (0.6868) | (0.6903) | (0.7364) | (0.4977) |
| SOE | 6.2988 | 5.7168 | 2.2455 | 3.3450 | 5.3659^*^ | -0.9501 |
|  | (1.4310) | (1.5287) | (1.0579) | (1.1672) | (1.7947) | (-1.1161) |
| Board | 8.3710 | 15.3057 | -2.4692 | -1.4001 | -0.7373 | -1.7720 |
|  | (0.6115) | (1.1843) | (-0.4248) | (-0.1833) | (-0.1066) | (-0.6922) |
| 2011.year | 0.0000 | 0.0000 | 0.0000 | 0.0000 | 0.0000 | 0.0000 |
|  | (.) | (.) | (.) | (.) | (.) | (.) |
| 2012.year | 12.2159^***^ | 10.8064^**^ | 6.8002^***^ | -4.5332 | -5.0805^*^ | -0.7115 |
|  | (2.7618) | (2.5164) | (2.9884) | (-1.6306) | (-1.7507) | (-0.8046) |
| 2013.year | 18.8568^**^ | 17.6301^**^ | 8.8536^**^ | -12.5377^**^ | -15.3372^**^ | -2.1269 |
|  | (2.5303) | (2.3954) | (2.4295) | (-1.9750) | (-2.1991) | (-1.1741) |
| 2014.year | 20.0474^***^ | 16.6469^**^ | 10.4954^***^ | -8.3449 | -10.7823^*^ | -1.2845 |
|  | (2.6063) | (2.3122) | (2.7178) | (-1.5540) | (-1.8874) | (-0.7376) |
| 2015.year | 33.2867^***^ | 24.9280^**^ | 19.0206^***^ | -18.0591^*^ | -23.1315^**^ | -2.8078 |
|  | (2.9057) | (2.3390) | (3.2964) | (-1.9121) | (-2.2303) | (-1.0038) |
| 2016.year | 40.9606^***^ | 30.8966^***^ | 22.4160^***^ | -15.8195 | -21.6925^**^ | -2.2447 |
|  | (3.2033) | (2.6591) | (3.4488) | (-1.6314) | (-2.0440) | (-0.7735) |
| 2017.year | 51.4891^***^ | 40.7508^***^ | 26.8493^***^ | -15.5776 | -22.8768^**^ | -1.3937 |
|  | (3.4829) | (2.9332) | (3.6829) | (-1.4817) | (-1.9954) | (-0.4366) |
| 2018.year | 63.3105^***^ | 47.8564^***^ | 31.5753^***^ | -17.0834 | -25.3978^**^ | -1.4585 |
|  | (3.7306) | (3.0538) | (3.8595) | (-1.4373) | (-1.9715) | (-0.3945) |
| 2019.year | 70.5104^***^ | 50.3825^***^ | 38.1847^***^ | -17.3826 | -28.1049^**^ | -0.4734 |
|  | (4.0005) | (3.1740) | (4.2636) | (-1.4191) | (-2.0861) | (-0.1211) |
| 2020.year | 48.6939^***^ | 44.0943^***^ | 16.0022^**^ | -29.1507^**^ | -32.4468^**^ | -10.5643^***^ |
|  | (3.0838) | (2.9014) | (2.1809) | (-2.2202) | (-2.2681) | (-2.7797) |
| 2021.year | -43.4352^**^ | -23.3251 | -28.4878^***^ | -53.9312^***^ | -53.3714^***^ | -21.8572^***^ |
|  | (-2.2881) | (-1.5742) | (-2.6483) | (-3.4277) | (-3.0077) | (-5.3458) |
| 1.province | 0.0000 | 0.0000 | 0.0000 | 0.0000 | 0.0000 | 0.0000 |
|  | (.) | (.) | (.) | (.) | (.) | (.) |
| 2.province | 26.5014^*^ | 19.8838 | 9.8993^*^ | -6.0831 | -3.7568 | -3.5683^*^ |
|  | (1.7964) | (1.6140) | (1.6702) | (-1.2244) | (-0.8568) | (-1.7573) |
| 3.province | -18.6707^*^ | -15.5378^*^ | -5.1688 | -4.8789 | -4.6340 | -2.4510 |
|  | (-1.7187) | (-1.6811) | (-0.9435) | (-0.9064) | (-0.8996) | (-1.0763) |
| 4.province | -10.8407 | -11.0760 | 0.6657 | -7.7336 | -5.5401 | 0.6338 |
|  | (-0.7695) | (-0.8924) | (0.0962) | (-1.0338) | (-1.1416) | (0.1771) |
| 5.province | -2.3702 | -1.4020 | -2.0765 | -5.6488 | -3.6973 | -2.2435 |
|  | (-0.2269) | (-0.1667) | (-0.4012) | (-1.4911) | (-1.1553) | (-1.3034) |
| 6.province | -23.7718^**^ | -13.4308 | -13.8108^**^ | -39.3421^***^ | -26.3803^***^ | -22.7692^***^ |
|  | (-2.0483) | (-1.3656) | (-2.2500) | (-5.0846) | (-3.8255) | (-3.6643) |
| 7.province | -14.5808 | -6.5346 | -10.8036 | -16.5483^**^ | -11.8478^**^ | -7.6336^***^ |
|  | (-0.9256) | (-0.4810) | (-1.1655) | (-2.5510) | (-2.0244) | (-2.7158) |
| 8.province | -15.1909 | -12.1587 | -9.6585 | -26.4698^*^ | -24.9878^**^ | -11.8598^**^ |
|  | (-1.2001) | (-1.1074) | (-1.5245) | (-1.9598) | (-2.0746) | (-2.3193) |
| 9.province | -21.6637^*^ | -17.8470^*^ | -7.0108 | -10.3955^**^ | -8.3269^*^ | -2.9791 |
|  | (-1.7544) | (-1.7529) | (-1.1037) | (-1.9797) | (-1.7151) | (-1.3062) |
| 10.province | -3.4392 | -10.1046 | -1.8460 | -7.3004 | -5.7415 | -3.7122 |
|  | (-0.3636) | (-1.1103) | (-0.3697) | (-0.9020) | (-0.7427) | (-1.5494) |
| 11.province | -15.7176 | -12.9656 | -6.8626 | -24.6903^***^ | -21.3185^***^ | -8.5352^**^ |
|  | (-0.9832) | (-1.0182) | (-0.7731) | (-3.1538) | (-2.9245) | (-2.5014) |
| 12.province | -0.1296 | 1.2513 | 2.5006 | -9.0016^*^ | -5.8631 | -3.2367 |
|  | (-0.0123) | (0.1451) | (0.4551) | (-1.8089) | (-1.3119) | (-1.4551) |
| 13.province | -21.0948^*^ | -13.3560 | -11.2292^*^ | -9.1606^*^ | -7.0151^*^ | -3.9010 |
|  | (-1.8493) | (-1.4342) | (-1.7330) | (-1.9045) | (-1.7294) | (-1.6431) |
| 14.province | -30.0502^**^ | -24.5175^**^ | -15.6749^**^ | -29.1971^***^ | -22.2924^***^ | -11.5591^***^ |
|  | (-2.0726) | (-1.9804) | (-2.1063) | (-3.3491) | (-2.8999) | (-2.9994) |
| 15.province | -2.6869 | 0.7517 | -0.7533 | -6.4865 | -3.8891 | -3.8151^**^ |
|  | (-0.3108) | (0.1050) | (-0.1694) | (-1.1877) | (-0.7803) | (-2.0700) |
| 16.province | -3.9809 | -6.6296 | 0.1148 | -19.0439^***^ | -18.9291^***^ | -6.0032^**^ |
|  | (-0.3367) | (-0.6001) | (0.0191) | (-3.0429) | (-3.2372) | (-2.3955) |
| 17.province | -26.1306^**^ | -17.7184 | -13.0483^**^ | -13.7491^**^ | -13.7155^***^ | -3.3878 |
|  | (-1.9795) | (-1.4914) | (-2.0938) | (-2.3301) | (-2.6803) | (-1.2685) |
| 18.province | -10.2227 | -9.5087 | -0.7706 | -24.7289^**^ | -20.9048^**^ | -5.1368 |
|  | (-0.9334) | (-1.1161) | (-0.1365) | (-2.3222) | (-2.5297) | (-1.0435) |
| 19.province | -22.7784 | -15.5387 | -16.2804 | -48.6733^***^ | -32.9803^***^ | -73.7887^***^ |
|  | (-1.1677) | (-0.9640) | (-1.5200) | (-3.3424) | (-2.5886) | (-12.1349) |
| 20.province | -20.4016 | -9.4350 | -9.6192 |  |  |  |
|  | (-1.6013) | (-0.9223) | (-1.2044) |  |  |  |
| 21.province | 1.1394 | -0.0373 | 0.6223 | -6.7542 | -6.5598 | -2.5413 |
|  | (0.1131) | (-0.0045) | (0.1211) | (-1.3721) | (-1.4947) | (-1.2089) |
| 22.province | -23.1890^*^ | -26.1615^**^ | -7.2577 | -29.6731^***^ | -29.0804^***^ | -10.5963^***^ |
|  | (-1.9309) | (-2.2351) | (-1.3179) | (-4.5579) | (-3.9943) | (-4.2001) |
| 23.province | 0.9111 | -7.2819 | 0.3005 | -12.0050^*^ | -14.9885^**^ | -3.0049 |
|  | (0.0842) | (-0.8163) | (0.0524) | (-1.7741) | (-2.5130) | (-0.9409) |
| 24.province | -14.2740 | -9.5301 | -6.9004 | -11.8421^**^ | -9.6539^**^ | -4.4508^**^ |
|  | (-1.4326) | (-1.1613) | (-1.3975) | (-2.5022) | (-2.2887) | (-2.0479) |
| 25.province | 12.0649 | 10.9711 | 5.8500 | -7.6566 | -5.7953 | -4.7625^*^ |
|  | (1.2411) | (1.4069) | (1.1339) | (-1.3026) | (-1.0472) | (-1.9517) |
| 26.province | -23.9939 | -17.9747 | -6.1605 | -23.2636^***^ | -20.9737^***^ | -8.1333^***^ |
|  | (-1.5809) | (-1.3879) | (-0.8219) | (-3.8138) | (-3.6981) | (-3.0849) |
| 27.province | -52.9028^**^ | -32.4379 | -38.7626^***^ | -35.6833^***^ | -38.3038^***^ | -8.8671^***^ |
|  | (-2.0800) | (-1.5764) | (-2.7369) | (-5.7450) | (-5.0553) | (-4.2863) |
| 28.province | -35.3543^***^ | -26.8448^**^ | -15.1526^**^ | 39.5440 | 20.5217 | 18.4648 |
|  | (-2.8399) | (-2.4489) | (-2.4583) | (0.8821) | (0.7385) | (1.0118) |
| 29.province | -7.0293 | -5.8046 | -2.8929 | -18.6325^**^ | -11.9231 | -15.4558^***^ |
|  | (-0.6674) | (-0.6151) | (-0.5308) | (-2.2922) | (-1.4057) | (-4.5271) |
| 30.province | -3.6607 | 0.1219 | -1.8276 | -11.2395^***^ | -9.0631^***^ | -4.6121^***^ |
|  | (-0.4476) | (0.0181) | (-0.4294) | (-2.9296) | (-2.7770) | (-2.6200) |
| 31.province | -6.8281 | -5.6028 | -4.6841 | -10.2432 | -9.1155 | -2.7658 |
|  | (-0.5724) | (-0.5552) | (-0.7164) | (-0.6728) | (-0.6292) | (-0.5406) |
| _cons | -5.5e+02^***^ | -5.0e+02^***^ | -2.4e+02^***^ | -3.1e+02^***^ | -2.7e+02^***^ | -1.2e+02^***^ |
|  | (-4.3327) | (-3.5934) | (-5.0633) | (-5.1054) | (-4.1525) | (-7.5841) |
| / |  |  |  |  |  |  |
| var(e.PAT) | 7.9e+03^***^ |  |  | 1.2e+03^***^ |  |  |
|  | (2.7406) |  |  | (3.0135) |  |  |
| var(e.INPAT) |  | 4.8e+03^**^ |  |  | 937.7389^**^ |  |
|  |  | (2.1344) |  |  | (2.3772) |  |
| var(e.UPAT) |  |  | 1.7e+03^***^ |  |  | 174.0151^***^ |
|  |  |  | (3.1359) |  |  | (6.5028) |
| *N* | 13816 | 13816 | 13816 | 8965 | 8965 | 8965 |
| pseudo *R*^2^ | 0.0384 | 0.0432 | 0.0540 | 0.0390 | 0.0398 | 0.0638 |

*t* statistics in parentheses

^*^ *p* < 0.1, ^**^ *p* < 0.05, ^***^ *p* < 0.01

**Table 9. Test results of mediating mechanism: information asymmetry reduction.**

|  | (1) | (2) | (3) | (4) |
| --- | --- | --- | --- | --- |
|  | PAT | ASY | PAT | PAT |
| DIF | 0.0313^***^ | -0.0009^***^ |  | 0.0187^***^ |
|  | (7.0883) | (-14.1697) |  | (4.1634) |
|  |  |  |  |  |
| Size | 8.1154^***^ | -0.2564^***^ | 4.7640^***^ | 4.5431^***^ |
|  | (12.5762) | (-31.5685) | (10.7245) | (10.4924) |
|  |  |  |  |  |
| Age | -13.6774^***^ | 0.0527^***^ | -11.1946^***^ | -12.9431^***^ |
|  | (-7.6385) | (4.7335) | (-7.2501) | (-7.3819) |
|  |  |  |  |  |
| lev | 0.2798 | 0.4086^***^ | 5.9037^***^ | 5.9733^***^ |
|  | (0.1584) | (15.9915) | (4.2492) | (4.2677) |
|  |  |  |  |  |
| Top1 | 12.2071^**^ | 0.5130^***^ | 19.3474^***^ | 19.3542^***^ |
|  | (3.1316) | (16.1489) | (4.8995) | (4.9027) |
|  |  |  |  |  |
| Mfee | 0.0037^***^ | 0.0001 | 0.0035^**^ | 0.0044^***^ |
|  | (3.3422) | (0.8571) | (2.8453) | (3.7408) |
|  |  |  |  |  |
| Growth | 0.4388 | -0.0002 | 0.4361 | 0.4366 |
|  | (1.8886) | (-0.0132) | (1.6771) | (1.7417) |
|  |  |  |  |  |
| ROE | 1.5939^*^ | -0.1869^***^ | -1.5422 | -1.0097 |
|  | (2.1068) | (-4.0271) | (-1.7856) | (-1.2131) |
|  |  |  |  |  |
| TobinQ | -1.7457^***^ | -0.1341^***^ | -3.2494^***^ | -3.6145^***^ |
|  | (-4.6938) | (-11.2540) | (-7.1932) | (-7.5258) |
|  |  |  |  |  |
| Mshare | -22.9440^***^ | 0.1560 | -19.4900^***^ | -20.7704^***^ |
|  | (-5.8281) | (1.7574) | (-4.8799) | (-5.0984) |
|  |  |  |  |  |
| Indep | 40.0440^***^ | -0.5546^***^ | 31.7705^***^ | 32.3172^***^ |
|  | (4.1228) | (-6.1272) | (3.3574) | (3.4012) |
|  |  |  |  |  |
| Dual | 2.5448^**^ | -0.0207^*^ | 2.3310^**^ | 2.2564^**^ |
|  | (3.1065) | (-2.4813) | (3.0127) | (2.8891) |
|  |  |  |  |  |
| SOE | 1.2175^*^ | 0.0467^***^ | 1.4892^*^ | 1.8678^**^ |
|  | (2.0381) | (5.5208) | (2.3985) | (2.9909) |
|  |  |  |  |  |
| Board | -0.4595 | -0.0327 | -1.7738 | -0.9156 |
|  | (-0.1909) | (-1.1861) | (-0.7477) | (-0.3888) |
|  |  |  |  |  |
| ASY |  |  | -14.1724^***^ | -13.9329^***^ |
|  |  |  | (-7.8536) | (-7.6638) |
|  |  |  |  |  |
| _cons | -1.6e+02^***^ | 5.6298^***^ | -81.4697^***^ | -77.5154^***^ |
|  | (-11.9723) | (36.6899) | (-7.8876) | (-7.5698) |
| F | 23.2996 | 243.7511 | 24.5772 | 23.2654 |
| r2_a | 0.0696 | 0.3344 | 0.0907 | 0.0912 |
| N | 2.3e+04 | 2.3e+04 | 2.3e+04 | 2.3e+04 |

*t* statistics in parentheses

^*^ *p* < 0.05, ^**^ *p* < 0.01, ^***^ *p* < 0.001

. sgmediation PAT , mv( ASY ) iv( DIF) cv( Size Age lev Top1 Mfee Growth ROE TobinQ Mshare Indep Dual SOE Board)

Model with dv regressed on iv (path c)

Source SS df MS Number of obs = 22,781

F(14, 22766) = 122.80

Model 3753753.94 14 268125.281 Prob > F = 0.0000

Residual 49706254.8 22,766 2183.35477 R-squared = 0.0702

Adj R-squared = 0.0696

Total 53460008.7 22,780 2346.79582 Root MSE = 46.726

PAT Coefficient Std. err. t P>t [95% conf. interval]

DIF .0312686 .0050584 6.18 0.000 .0213539 .0411833

Size 8.115412 .2781676 29.17 0.000 7.570185 8.66064

Age -13.67738 1.041053 -13.14 0.000 -15.71792 -11.63685

lev .2797599 1.424866 0.20 0.844 -2.513074 3.072594

Top1 12.20709 2.170809 5.62 0.000 7.95216 16.46203

Mfee .0036814 .0220919 0.17 0.868 -.0396202 .0469829

Growth .4388225 .3660334 1.20 0.231 -.2786279 1.156273

ROE 1.593928 1.414874 1.13 0.260 -1.179322 4.367179

TobinQ -1.745722 .4290897 -4.07 0.000 -2.586767 -.9046774

Mshare -22.94398 6.738755 -3.40 0.001 -36.1524 -9.735563

Indep 40.04403 6.519793 6.14 0.000 27.26479 52.82326

Dual 2.544813 .7705386 3.30 0.001 1.034505 4.055121

SOE 1.217482 .7176025 1.70 0.090 -.1890676 2.624032

Board -.4595285 1.900468 -0.24 0.809 -4.184575 3.265518

_cons -155.9543 7.594825 -20.53 0.000 -170.8407 -141.0679

Model with mediator regressed on iv (path a)

Source SS df MS Number of obs = 22,781

F(14, 22766) = 818.50

Model 2989.14212 14 213.510152 Prob > F = 0.0000

Residual 5938.65484 22,766 .260856314 R-squared = 0.3348

Adj R-squared = 0.3344

Total 8927.79696 22,780 .391913826 Root MSE = .51074

ASY Coefficient Std. err. t P>t [95% conf. interval]

DIF -.0009019 .0000553 -16.31 0.000 -.0010103 -.0007936

Size -.256396 .0030405 -84.33 0.000 -.2623555 -.2504364

Age .0527033 .0113792 4.63 0.000 .0303993 .0750072

lev .4086377 .0155744 26.24 0.000 .3781107 .4391647

Top1 .5129667 .0237279 21.62 0.000 .4664583 .5594751

Mfee .0000522 .0002415 0.22 0.829 -.0004211 .0005255

Growth -.0001587 .0040009 -0.04 0.968 -.0080008 .0076834

ROE -.1868676 .0154652 -12.08 0.000 -.2171805 -.1565547

TobinQ -.1341283 .0046901 -28.60 0.000 -.1433213 -.1249353

Mshare .1560063 .0736577 2.12 0.034 .0116322 .3003804

Indep -.5545775 .0712643 -7.78 0.000 -.6942604 -.4148945

Dual -.0206971 .0084223 -2.46 0.014 -.0372055 -.0041888

SOE .0466784 .0078437 5.95 0.000 .0313042 .0620526

Board -.0327301 .020773 -1.58 0.115 -.0734465 .0079864

_cons 5.629772 .083015 67.82 0.000 5.467057 5.792486

Model with dv regressed on mediator and iv (paths b and c')

Source SS df MS Number of obs = 22,781

F(15, 22765) = 153.37

Model 4906594.71 15 327106.314 Prob > F = 0.0000

Residual 48553414 22,765 2132.80975 R-squared = 0.0918

Adj R-squared = 0.0912

Total 53460008.7 22,780 2346.79582 Root MSE = 46.182

PAT Coefficient Std. err. t P>t [95% conf. interval]

ASY -13.93287 .5992831 -23.25 0.000 -15.10751 -12.75824

DIF .0187021 .0050286 3.72 0.000 .0088457 .0285585

Size 4.54308 .314953 14.42 0.000 3.925751 5.16041

Age -12.94307 1.029417 -12.57 0.000 -14.9608 -10.92535

lev 5.973256 1.42941 4.18 0.000 3.171515 8.774997

Top1 19.35419 2.167446 8.93 0.000 15.10585 23.60254

Mfee .0044083 .0218347 0.20 0.840 -.0383891 .0472058

Growth .4366115 .3617718 1.21 0.227 -.2724858 1.145709

ROE -1.009674 1.402878 -0.72 0.472 -3.759411 1.740062

TobinQ -3.614514 .4316441 -8.37 0.000 -4.460566 -2.768462

Mshare -20.77037 6.660952 -3.12 0.002 -33.82629 -7.714445

Indep 32.31717 6.452449 5.01 0.000 19.66993 44.96441

Dual 2.256442 .7616683 2.96 0.003 .7635205 3.749364

SOE 1.867846 .709799 2.63 0.009 .4765921 3.259101

Board -.9155522 1.878444 -0.49 0.626 -4.59743 2.766325

_cons -77.5154 8.229748 -9.42 0.000 -93.64627 -61.38453

Sobel-Goodman Mediation Tests

Coef Std Err Z P>Z

Sobel .01256649 .00094106 13.35 0

Goodman-1 (Aroian) .01256649 .00094164 13.35 0

Goodman-2 .01256649 .00094048 13.36 0

Coef Std Err Z P>Z

a coefficient = -.000902 .000055 -16.3127 0

b coefficient = -13.9329 .599283 -23.2492 0

Indirect effect = .012566 .000941 13.3535 0

Direct effect = .018702 .005029 3.71915 .0002

Total effect = .031269 .005058 6.18157 6.3e-10

Proportion of total effect that is mediated: .40188844

Ratio of indirect to direct effect: .67192888

Ratio of total to direct effect: 1.6719289

.

. outreg2 using ASY1.rtf, replace tstat bdec(4) tdec(4) sym(***, **, *) ctitle(satisfaction) e( r2 F )

ASY1.rtf

dir : seeout

.

. bootstrap r(ind_eff) r(dir_eff),reps(1000) : sgmediation PAT , mv( ASY ) iv( DIF) cv( Size Age lev Top1 Mfee Growth ROE TobinQ

> Mshare Indep Dual SOE Board) quietly

(running sgmediation on estimation sample)

Bootstrap replications (1,000)

1 ---+--- 2 ---+--- 3 ---+--- 4 ---+--- 5

.................................................. 50

.................................................. 100

.................................................. 150

.................................................. 200

.................................................. 250

.................................................. 300

.................................................. 350

.................................................. 400

.................................................. 450

.................................................. 500

.................................................. 550

.................................................. 600

.................................................. 650

.................................................. 700

.................................................. 750

.................................................. 800

.................................................. 850

.................................................. 900

.................................................. 950

.................................................. 1,000

Bootstrap results Number of obs = 22,781

Replications = 1,000

Command: sgmediation PAT, mv( ASY ) iv( DIF) cv( Size Age lev Top1 Mfee Growth ROE TobinQ Mshare Indep Dual SOE Board)

quietly

_bs_1: r(ind_eff)

_bs_2: r(dir_eff)

Observed Bootstrap Normal-based

coefficient std. err. z P>z [95% conf. interval]

_bs_1 .0125665 .0019548 6.43 0.000 .0087352 .0163977

_bs_2 .0187021 .0044466 4.21 0.000 .009987 .0274172

.

. estat bootstrap, percentile bc

Bootstrap results Number of obs = 22,781

Replications = 1000

Command: sgmediation PAT, mv( ASY ) iv( DIF) cv( Size Age lev Top1 Mfee Growth ROE TobinQ Mshare Indep Dual SOE Board)

quietly

_bs_1: r(ind_eff)

_bs_2: r(dir_eff)

Observed Bootstrap

coefficient Bias std. err. [95% conf. interval]

_bs_1 .01256649 .000077 .00195475 .0091121 .0167007 (P)

.0090325 .0166394 (BC)

_bs_2 .01870212 -.0002606 .00444657 .0099368 .0271148 (P)

.0101978 .0274282 (BC)

Key: P: Percentile

BC: Bias-corrected

**Table 10. Test results of mediating mechanism: consumption structure optimization.**

|  | (1) | (2) | (3) | (4) |
| --- | --- | --- | --- | --- |
|  | PAT | struct | PAT | PAT |
| DIF | 0.0186^***^ | 135.4656^***^ |  | 0.0092^***^ |
|  | (11.1305) | (110.9647) |  | (4.3799) |
|  |  |  |  |  |
| Size | 4.1842^***^ | 560.0970^***^ | 4.2197^***^ | 4.1453^***^ |
|  | (20.9486) | (7.4748) | (21.6741) | (20.8302) |
|  |  |  |  |  |
| Age | -5.2247^***^ | -3.9e+03^***^ | -4.3106^***^ | -4.9522^***^ |
|  | (-11.9460) | (-17.8263) | (-10.3081) | (-11.7184) |
|  |  |  |  |  |
| lev | 1.4406^**^ | -1.4e+03^***^ | 1.5204^**^ | 1.5379^**^ |
|  | (2.9716) | (-4.3928) | (3.1658) | (3.1830) |
|  |  |  |  |  |
| Top1 | 1.4172 | -3.5e+03^***^ | 1.7045 | 1.6583 |
|  | (1.2939) | (-6.5394) | (1.5541) | (1.5094) |
|  |  |  |  |  |
| Mfee | 0.0008 | -2.0399^**^ | 0.0007 | 0.0010^*^ |
|  | (1.8704) | (-3.0806) | (1.6299) | (2.2057) |
|  |  |  |  |  |
| Growth | 0.1270 | 1.0e+03^***^ | 0.0292 | 0.0552 |
|  | (1.1601) | (5.2728) | (0.2803) | (0.5231) |
|  |  |  |  |  |
| ROE | 1.4586^***^ | 877.8716 | 1.2230^***^ | 1.3976^***^ |
|  | (3.8778) | (1.2402) | (3.5045) | (3.8720) |
|  |  |  |  |  |
| TobinQ | -1.3231^***^ | -1.4e+02 | -1.1858^***^ | -1.3136^***^ |
|  | (-8.2853) | (-1.2065) | (-7.9283) | (-8.1830) |
|  |  |  |  |  |
| Mshare | -8.4127^***^ | 2.7e+03 | -8.2830^***^ | -8.6035^***^ |
|  | (-4.4977) | (1.7230) | (-4.3973) | (-4.5745) |
|  |  |  |  |  |
| Indep | 14.9400^***^ | -6.6e+02 | 14.8730^***^ | 14.9860^***^ |
|  | (5.2012) | (-0.4338) | (5.1822) | (5.2246) |
|  |  |  |  |  |
| Dual | 1.2389^***^ | 1.1e+03^***^ | 1.1614^***^ | 1.1636^***^ |
|  | (4.4963) | (6.3788) | (4.1865) | (4.1957) |
|  |  |  |  |  |
| SOE | -0.0364 | 484.1998^**^ | -0.2042 | -0.0701 |
|  | (-0.1480) | (2.8515) | (-0.8338) | (-0.2856) |
|  |  |  |  |  |
| Board | 1.0380 | -1.1e+03^*^ | 0.8754 | 1.1142 |
|  | (1.2378) | (-2.4838) | (1.0425) | (1.3261) |
|  |  |  |  |  |
| struct |  |  | 0.0001^***^ | 0.0001^***^ |
|  |  |  | (9.9015) | (5.7971) |
|  |  |  |  |  |
| _cons | -84.9128^***^ | 4.7e+03^*^ | -87.0219^***^ | -85.2428^***^ |
|  | (-19.9255) | (2.4621) | (-20.9770) | (-19.9393) |
| F | 70.7043 | 1.3e+03 | 62.2080 | 68.7575 |
| r2_a | 0.1367 | 0.4638 | 0.1379 | 0.1385 |
| N | 2.3e+04 | 2.3e+04 | 2.3e+04 | 2.3e+04 |

*t* statistics in parentheses

^*^ *p* < 0.05, ^**^ *p* < 0.01, ^***^ *p* < 0.001

. sgmediation PAT , mv( struct ) iv( DIF) cv( Size Age lev Top1 Mfee Growth ROE TobinQ Mshare Indep Dual SOE Board)

Model with dv regressed on iv (path c)

Source SS df MS Number of obs = 22,781

F(14, 22766) = 258.62

Model 951723.583 14 67980.2559 Prob > F = 0.0000

Residual 5984288.01 22,766 262.860758 R-squared = 0.1372

Adj R-squared = 0.1367

Total 6936011.59 22,780 304.478121 Root MSE = 16.213

PAT Coefficient Std. err. t P>t [95% conf. interval]

DIF .0185916 .0017551 10.59 0.000 .0151515 .0220318

Size 4.184213 .0965178 43.35 0.000 3.995032 4.373395

Age -5.224706 .3612214 -14.46 0.000 -5.932725 -4.516687

lev 1.440602 .4943957 2.91 0.004 .4715531 2.409652

Top1 1.417167 .753221 1.88 0.060 -.0591974 2.893532

Mfee .000849 .0076654 0.11 0.912 -.0141757 .0158736

Growth .1270188 .1270052 1.00 0.317 -.12192 .3759577

ROE 1.458636 .4909289 2.97 0.003 .4963821 2.42089

TobinQ -1.32311 .1488843 -8.89 0.000 -1.614934 -1.031287

Mshare -8.412719 2.338193 -3.60 0.000 -12.99574 -3.829701

Indep 14.93997 2.262218 6.60 0.000 10.50587 19.37407

Dual 1.238947 .2673592 4.63 0.000 .7149052 1.76299

SOE -.0364102 .2489916 -0.15 0.884 -.5244507 .4516302

Board 1.038026 .6594188 1.57 0.115 -.2544793 2.330532

_cons -84.91277 2.63523 -32.22 0.000 -90.078 -79.74754

Model with mediator regressed on iv (path a)

Source SS df MS Number of obs = 22,781

F(14, 22766) = 1408.48

Model 2.3160e+12 14 1.6543e+11 Prob > F = 0.0000

Residual 2.6738e+12 22,766 117449100 R-squared = 0.4641

Adj R-squared = 0.4638

Total 4.9898e+12 22,780 219043029 Root MSE = 10837

struct Coefficient Std. err. t P>t [95% conf. interval]

DIF 135.4656 1.173201 115.47 0.000 133.166 137.7651

Size 560.097 64.51627 8.68 0.000 433.6407 686.5532

Age -3921.096 241.4546 -16.24 0.000 -4394.363 -3447.828

lev -1399.392 330.4735 -4.23 0.000 -2047.142 -751.641

Top1 -3469.509 503.4825 -6.89 0.000 -4456.369 -2482.649

Mfee -2.039873 5.123833 -0.40 0.691 -12.08294 8.00319

Growth 1033.268 84.89526 12.17 0.000 866.8676 1199.669

ROE 877.8716 328.1562 2.68 0.007 234.6631 1521.08

TobinQ -136.2418 99.52009 -1.37 0.171 -331.308 58.82434

Mshare 2744.46 1562.94 1.76 0.079 -319.0095 5807.929

Indep -662.5055 1512.156 -0.44 0.661 -3626.434 2301.423

Dual 1083.985 178.7134 6.07 0.000 733.6949 1434.276

SOE 484.1998 166.4358 2.91 0.004 157.9743 810.4252

Board -1096.748 440.7814 -2.49 0.013 -1960.709 -232.7859

_cons 4748.674 1761.491 2.70 0.007 1296.031 8201.317

Model with dv regressed on mediator and iv (paths b and c')

Source SS df MS Number of obs = 22,781

F(15, 22765) = 245.17

Model 964638.102 15 64309.2068 Prob > F = 0.0000

Residual 5971373.49 22,765 262.305007 R-squared = 0.1391

Adj R-squared = 0.1385

Total 6936011.59 22,780 304.478121 Root MSE = 16.196

PAT Coefficient Std. err. t P>t [95% conf. interval]

struct .0000695 9.90e-06 7.02 0.000 .0000501 .0000889

DIF .0091771 .0022078 4.16 0.000 .0048497 .0135045

Size 4.145288 .0965751 42.92 0.000 3.955994 4.334582

Age -4.952199 .3629233 -13.65 0.000 -5.663553 -4.240844

lev 1.537857 .4940672 3.11 0.002 .5694515 2.506262

Top1 1.65829 .7532086 2.20 0.028 .18195 3.134631

Mfee .0009907 .0076573 0.13 0.897 -.0140181 .0159995

Growth .055209 .127283 0.43 0.664 -.1942743 .3046923

ROE 1.397626 .4904867 2.85 0.004 .4362386 2.359014

TobinQ -1.313642 .1487329 -8.83 0.000 -1.605168 -1.022115

Mshare -8.603453 2.335878 -3.68 0.000 -13.18193 -4.024972

Indep 14.98601 2.259835 6.63 0.000 10.55658 19.41544

Dual 1.163613 .2672921 4.35 0.000 .6397021 1.687524

SOE -.0700611 .2487745 -0.28 0.778 -.5576759 .4175538

Board 1.114248 .6588109 1.69 0.091 -.1770663 2.405562

_cons -85.24279 2.632863 -32.38 0.000 -90.40338 -80.0822

Sobel-Goodman Mediation Tests

Coef Std Err Z P>Z

Sobel .00941456 .0013442 7.004 2.491e-12

Goodman-1 (Aroian) .00941456 .00134425 7.004 2.495e-12

Goodman-2 .00941456 .00134415 7.004 2.486e-12

Coef Std Err Z P>Z

a coefficient = 135.466 1.1732 115.467 0

b coefficient = .000069 9.9e-06 7.01675 2.3e-12

Indirect effect = .009415 .001344 7.00383 2.5e-12

Direct effect = .009177 .002208 4.15674 .000032

Total effect = .018592 .001755 10.5927 0

Proportion of total effect that is mediated: .50638629

Ratio of indirect to direct effect: 1.0258757

Ratio of total to direct effect: 2.0258757

.

. outreg2 using struct1.rtf, replace tstat bdec(4) tdec(4) sym(***, **, *) ctitle(satisfaction) e( r2 F )

struct1.rtf

dir : seeout

.

. bootstrap r(ind_eff) r(dir_eff),reps(1000) : sgmediation PAT , mv( struct ) iv( DIF) cv( Size Age lev Top1 Mfee Growth ROE Tobi

> nQ Mshare Indep Dual SOE Board) quietly

(running sgmediation on estimation sample)

Bootstrap replications (1,000)

1 ---+--- 2 ---+--- 3 ---+--- 4 ---+--- 5

.................................................. 50

.................................................. 100

.................................................. 150

.................................................. 200

.................................................. 250

.................................................. 300

.................................................. 350

.................................................. 400

.................................................. 450

.................................................. 500

.................................................. 550

.................................................. 600

.................................................. 650

.................................................. 700

.................................................. 750

.................................................. 800

.................................................. 850

.................................................. 900

.................................................. 950

.................................................. 1,000

Bootstrap results Number of obs = 22,781

Replications = 1,000

Command: sgmediation PAT, mv( struct ) iv( DIF) cv( Size Age lev Top1 Mfee Growth ROE TobinQ Mshare Indep Dual SOE Board)

quietly

_bs_1: r(ind_eff)

_bs_2: r(dir_eff)

Observed Bootstrap Normal-based

coefficient std. err. z P>z [95% conf. interval]

_bs_1 .0094146 .00162 5.81 0.000 .0062394 .0125897

_bs_2 .0091771 .0020801 4.41 0.000 .0051001 .0132541

.

. estat bootstrap, percentile bc

Bootstrap results Number of obs = 22,781

Replications = 1000

Command: sgmediation PAT, mv( struct ) iv( DIF) cv( Size Age lev Top1 Mfee Growth ROE TobinQ Mshare Indep Dual SOE Board)

quietly

_bs_1: r(ind_eff)

_bs_2: r(dir_eff)

Observed Bootstrap

coefficient Bias std. err. [95% conf. interval]

_bs_1 .00941456 -.0000533 .00161999 .0062553 .0126176 (P)

.0064807 .0127145 (BC)

_bs_2 .00917709 -7.57e-06 .00208012 .0049939 .01333 (P)

.0049854 .0132948 (BC)

Key: P: Percentile

BC: Bias-corrected

**Table 11. Test results of mediating mechanism: total consumption increased.**

|  | (1) | (2) | (3) | (4) |
| --- | --- | --- | --- | --- |
|  | PAT | total | PAT | PAT |
| DIF | 0.0186^***^ | 81.0888^***^ |  | 0.0085^***^ |
|  | (11.1305) | (110.1486) |  | (4.0808) |
|  |  |  |  |  |
| Size | 4.1842^***^ | 322.9818^***^ | 4.2158^***^ | 4.1439^***^ |
|  | (20.9486) | (6.9499) | (21.6880) | (20.8193) |
|  |  |  |  |  |
| Age | -5.2247^***^ | -2.4e+03^***^ | -4.3188^***^ | -4.9233^***^ |
|  | (-11.9460) | (-17.2275) | (-10.3692) | (-11.6535) |
|  |  |  |  |  |
| lev | 1.4406^**^ | -9.0e+02^***^ | 1.5358^**^ | 1.5524^**^ |
|  | (2.9716) | (-4.5432) | (3.2039) | (3.2190) |
|  |  |  |  |  |
| Top1 | 1.4172 | -2.0e+03^***^ | 1.7091 | 1.6722 |
|  | (1.2939) | (-6.3628) | (1.5607) | (1.5245) |
|  |  |  |  |  |
| Mfee | 0.0008 | -1.6385^***^ | 0.0008 | 0.0011^*^ |
|  | (1.8704) | (-3.6517) | (1.8006) | (2.3266) |
|  |  |  |  |  |
| Growth | 0.1270 | 713.5912^***^ | 0.0116 | 0.0380 |
|  | (1.1601) | (5.3409) | (0.1111) | (0.3615) |
|  |  |  |  |  |
| ROE | 1.4586^***^ | 590.7497 | 1.2184^***^ | 1.3850^***^ |
|  | (3.8778) | (1.2399) | (3.4868) | (3.8473) |
|  |  |  |  |  |
| TobinQ | -1.3231^***^ | -1.2e+02^*^ | -1.1854^***^ | -1.3079^***^ |
|  | (-8.2853) | (-1.9892) | (-7.9907) | (-8.1810) |
|  |  |  |  |  |
| Mshare | -8.4127^***^ | 1.1e+03 | -8.2149^***^ | -8.5449^***^ |
|  | (-4.4977) | (1.1642) | (-4.3683) | (-4.5504) |
|  |  |  |  |  |
| Indep | 14.9400^***^ | -69.4059 | 14.8283^***^ | 14.9486^***^ |
|  | (5.2012) | (-0.0734) | (5.1690) | (5.2144) |
|  |  |  |  |  |
| Dual | 1.2389^***^ | 804.8435^***^ | 1.1325^***^ | 1.1386^***^ |
|  | (4.4963) | (7.6436) | (4.0855) | (4.1085) |
|  |  |  |  |  |
| SOE | -0.0364 | 291.1055^**^ | -0.1992 | -0.0727 |
|  | (-0.1480) | (2.7475) | (-0.8138) | (-0.2963) |
|  |  |  |  |  |
| Board | 1.0380 | -7.1e+02^**^ | 0.9010 | 1.1271 |
|  | (1.2378) | (-2.5923) | (1.0732) | (1.3416) |
|  |  |  |  |  |
| total |  |  | 0.0002^***^ | 0.0001^***^ |
|  |  |  | (10.6382) | (6.6021) |
|  |  |  |  |  |
| _cons | -84.9128^***^ | 6.2e+03^***^ | -87.4946^***^ | -85.6904^***^ |
|  | (-19.9255) | (5.1605) | (-21.0872) | (-19.9690) |
| F | 70.7043 | 1.3e+03 | 62.3932 | 68.0055 |
| r2_a | 0.1367 | 0.4452 | 0.1384 | 0.1390 |
| N | 2.3e+04 | 2.3e+04 | 2.3e+04 | 2.3e+04 |

*t* statistics in parentheses

^*^ *p* < 0.05, ^**^ *p* < 0.01, ^***^ *p* < 0.001

. sgmediation PAT , mv( total ) iv( DIF) cv( Size Age lev Top1 Mfee Growth ROE TobinQ Mshare Indep Dual SOE Board)

Model with dv regressed on iv (path c)

Source SS df MS Number of obs = 22,781

F(14, 22766) = 258.62

Model 951723.583 14 67980.2559 Prob > F = 0.0000

Residual 5984288.01 22,766 262.860758 R-squared = 0.1372

Adj R-squared = 0.1367

Total 6936011.59 22,780 304.478121 Root MSE = 16.213

PAT Coefficient Std. err. t P>t [95% conf. interval]

DIF .0185916 .0017551 10.59 0.000 .0151515 .0220318

Size 4.184213 .0965178 43.35 0.000 3.995032 4.373395

Age -5.224706 .3612214 -14.46 0.000 -5.932725 -4.516687

lev 1.440602 .4943957 2.91 0.004 .4715531 2.409652

Top1 1.417167 .753221 1.88 0.060 -.0591974 2.893532

Mfee .000849 .0076654 0.11 0.912 -.0141757 .0158736

Growth .1270188 .1270052 1.00 0.317 -.12192 .3759577

ROE 1.458636 .4909289 2.97 0.003 .4963821 2.42089

TobinQ -1.32311 .1488843 -8.89 0.000 -1.614934 -1.031287

Mshare -8.412719 2.338193 -3.60 0.000 -12.99574 -3.829701

Indep 14.93997 2.262218 6.60 0.000 10.50587 19.37407

Dual 1.238947 .2673592 4.63 0.000 .7149052 1.76299

SOE -.0364102 .2489916 -0.15 0.884 -.5244507 .4516302

Board 1.038026 .6594188 1.57 0.115 -.2544793 2.330532

_cons -84.91277 2.63523 -32.22 0.000 -90.078 -79.74754

Model with mediator regressed on iv (path a)

Source SS df MS Number of obs = 22,781

F(14, 22766) = 1306.79

Model 8.2763e+11 14 5.9116e+10 Prob > F = 0.0000

Residual 1.0299e+12 22,766 45237818.1 R-squared = 0.4456

Adj R-squared = 0.4452

Total 1.8575e+12 22,780 81541453.5 Root MSE = 6725.9

total Coefficient Std. err. t P>t [95% conf. interval]

DIF 81.08883 .7281121 111.37 0.000 79.66168 82.51598

Size 322.9818 40.04011 8.07 0.000 244.5004 401.4631

Age -2416.872 149.8516 -16.13 0.000 -2710.592 -2123.153

lev -896.7206 205.0986 -4.37 0.000 -1298.728 -494.7134

Top1 -2044.989 312.4715 -6.54 0.000 -2657.455 -1432.524

Mfee -1.638545 3.179955 -0.52 0.606 -7.871474 4.594383

Growth 713.5912 52.68772 13.54 0.000 610.3197 816.8627

ROE 590.7497 203.6604 2.90 0.004 191.5615 989.938

TobinQ -122.1944 61.76419 -1.98 0.048 -243.2565 -1.132416

Mshare 1059.549 969.9924 1.09 0.275 -841.7019 2960.801

Indep -69.40585 938.4745 -0.07 0.941 -1908.88 1770.068

Dual 804.8435 110.9132 7.26 0.000 587.4462 1022.241

SOE 291.1055 103.2934 2.82 0.005 88.6434 493.5676

Board -714.3635 273.5579 -2.61 0.009 -1250.556 -178.1714

_cons 6234.579 1093.217 5.70 0.000 4091.799 8377.36

Model with dv regressed on mediator and iv (paths b and c')

Source SS df MS Number of obs = 22,781

F(15, 22765) = 246.09

Model 967744.503 15 64516.3002 Prob > F = 0.0000

Residual 5968267.09 22,765 262.168552 R-squared = 0.1395

Adj R-squared = 0.1390

Total 6936011.59 22,780 304.478121 Root MSE = 16.192

PAT Coefficient Std. err. t P>t [95% conf. interval]

total .0001247 .000016 7.82 0.000 .0000935 .000156

DIF .0084779 .0021786 3.89 0.000 .0042078 .0127481

Size 4.14393 .0965283 42.93 0.000 3.954728 4.333132

Age -4.923264 .3628006 -13.57 0.000 -5.634378 -4.212151

lev 1.552445 .4939516 3.14 0.002 .5842661 2.520624

Top1 1.672226 .7529358 2.22 0.026 .1964204 3.148032

Mfee .0010533 .0076553 0.14 0.891 -.0139516 .0160583

Growth .038017 .1273478 0.30 0.765 -.2115934 .2876274

ROE 1.384956 .4903727 2.82 0.005 .4237917 2.34612

TobinQ -1.30787 .1487009 -8.80 0.000 -1.599334 -1.016406

Mshare -8.54487 2.335174 -3.66 0.000 -13.12197 -3.96777

Indep 14.94863 2.259238 6.62 0.000 10.52037 19.37689

Dual 1.138564 .2673156 4.26 0.000 .6146075 1.662521

SOE -.0727181 .2487069 -0.29 0.770 -.5602005 .4147644

Board 1.127125 .6586486 1.71 0.087 -.1638715 2.418121

_cons -85.69037 2.633637 -32.54 0.000 -90.85248 -80.52826

Sobel-Goodman Mediation Tests

Coef Std Err Z P>Z

Sobel .01011371 .00129695 7.798 6.217e-15

Goodman-1 (Aroian) .01011371 .00129701 7.798 6.217e-15

Goodman-2 .01011371 .0012969 7.798 6.217e-15

Coef Std Err Z P>Z

a coefficient = 81.0888 .728112 111.369 0

b coefficient = .000125 .000016 7.81724 5.3e-15

Indirect effect = .010114 .001297 7.79805 6.2e-15

Direct effect = .008478 .002179 3.89149 .0001

Total effect = .018592 .001755 10.5927 0

Proportion of total effect that is mediated: .54399222

Ratio of indirect to direct effect: 1.192945

Ratio of total to direct effect: 2.192945

.

. outreg2 using total1.rtf, replace tstat bdec(4) tdec(4) sym(***, **, *) ctitle(satisfaction) e( r2 F )

total1.rtf

dir : seeout

.

. bootstrap r(ind_eff) r(dir_eff),reps(1000) : sgmediation PAT , mv( total ) iv( DIF) cv( Size Age lev Top1 Mfee Growth ROE Tobin

> Q Mshare Indep Dual SOE Board) quietly

(running sgmediation on estimation sample)

Bootstrap replications (1,000)

1 ---+--- 2 ---+--- 3 ---+--- 4 ---+--- 5

.................................................. 50

.................................................. 100

.................................................. 150

.................................................. 200

.................................................. 250

.................................................. 300

.................................................. 350

.................................................. 400

.................................................. 450

.................................................. 500

.................................................. 550

.................................................. 600

.................................................. 650

.................................................. 700

.................................................. 750

.................................................. 800

.................................................. 850

.................................................. 900

.................................................. 950

.................................................. 1,000

Bootstrap results Number of obs = 22,781

Replications = 1,000

Command: sgmediation PAT, mv( total ) iv( DIF) cv( Size Age lev Top1 Mfee Growth ROE TobinQ Mshare Indep Dual SOE Board)

quietly

_bs_1: r(ind_eff)

_bs_2: r(dir_eff)

Observed Bootstrap Normal-based

coefficient std. err. z P>z [95% conf. interval]

_bs_1 .0101137 .0014893 6.79 0.000 .0071948 .0130326

_bs_2 .0084779 .0020586 4.12 0.000 .0044431 .0125127

.

. estat bootstrap, percentile bc

Bootstrap results Number of obs = 22,781

Replications = 1000

Command: sgmediation PAT, mv( total ) iv( DIF) cv( Size Age lev Top1 Mfee Growth ROE TobinQ Mshare Indep Dual SOE Board)

quietly

_bs_1: r(ind_eff)

_bs_2: r(dir_eff)

Observed Bootstrap

coefficient Bias std. err. [95% conf. interval]

_bs_1 .01011371 -.0000402 .00148926 .0071226 .0130336 (P)

.0072708 .0130787 (BC)

_bs_2 .00847794 .0000356 .00205861 .0043991 .0125586 (P)

.004193 .0123943 (BC)

Key: P: Percentile

BC: Bias-corrected

**Table 12. Test results of mediating mechanism: factor market distortion mitigation.**

|  | (1) | (2) | (3) | (4) |
| --- | --- | --- | --- | --- |
|  | PAT | Dist | PAT | PAT |
| DIF | 0.0313^***^ | -0.0117^***^ |  | 0.0301^***^ |
|  | (7.0883) | (-8.4393) |  | (7.0359) |
|  |  |  |  |  |
| Size | 8.1154^***^ | -0.1563^*^ | 8.5569^***^ | 8.1000^***^ |
|  | (12.5762) | (-1.9902) | (13.0801) | (12.5693) |
|  |  |  |  |  |
| Age | -13.6774^***^ | -1.7861^***^ | -11.0515^***^ | -13.8538^***^ |
|  | (-7.6385) | (-6.1383) | (-6.8627) | (-7.5919) |
|  |  |  |  |  |
| lev | 0.2798 | 1.8678^***^ | 0.2084 | 0.4643 |
|  | (0.1584) | (4.6870) | (0.1185) | (0.2614) |
|  |  |  |  |  |
| Top1 | 12.2071^**^ | 2.5060^***^ | 12.2657^**^ | 12.4547^**^ |
|  | (3.1316) | (4.1302) | (3.1363) | (3.1774) |
|  |  |  |  |  |
| Mfee | 0.0037^***^ | -0.0037^***^ | 0.0018 | 0.0033^**^ |
|  | (3.3422) | (-5.0224) | (1.6904) | (3.1670) |
|  |  |  |  |  |
| Growth | 0.4388 | -0.9792^***^ | 0.3329 | 0.3421 |
|  | (1.8886) | (-6.4549) | (1.5144) | (1.5502) |
|  |  |  |  |  |
| ROE | 1.5939^*^ | 0.6982 | 0.8772 | 1.6629^*^ |
|  | (2.1068) | (1.3792) | (1.2017) | (2.2572) |
|  |  |  |  |  |
| TobinQ | -1.7457^***^ | -0.1407 | -1.1159^***^ | -1.7596^***^ |
|  | (-4.6938) | (-1.3637) | (-3.4654) | (-4.7340) |
|  |  |  |  |  |
| Mshare | -22.9440^***^ | -3.7953^*^ | -21.3338^***^ | -23.3189^***^ |
|  | (-5.8281) | (-2.4277) | (-5.5405) | (-5.8691) |
|  |  |  |  |  |
| Indep | 40.0440^***^ | 0.9431 | 39.4736^***^ | 40.1372^***^ |
|  | (4.1228) | (0.5288) | (4.0709) | (4.1306) |
|  |  |  |  |  |
| Dual | 2.5448^**^ | 1.0256^***^ | 2.7840^***^ | 2.6461^**^ |
|  | (3.1065) | (4.8702) | (3.4510) | (3.2638) |
|  |  |  |  |  |
| SOE | 1.2175^*^ | -2.9981^***^ | 0.2628 | 0.9213 |
|  | (2.0381) | (-15.8264) | (0.4668) | (1.5801) |
|  |  |  |  |  |
| Board | -0.4595 | 1.4340^**^ | -1.6860 | -0.3179 |
|  | (-0.1909) | (2.7759) | (-0.6898) | (-0.1316) |
|  |  |  |  |  |
| Dist |  |  | -0.1074^***^ | -0.0988^***^ |
|  |  |  | (-4.0957) | (-3.8467) |
|  |  |  |  |  |
| _cons | -1.6e+02^***^ | 21.5859^***^ | -1.6e+02^***^ | -1.5e+02^***^ |
|  | (-11.9723) | (10.3468) | (-12.5865) | (-11.9501) |
| F | 23.2996 | 62.4946 | 24.4411 | 23.8649 |
| r2_a | 0.0696 | 0.0352 | 0.0688 | 0.0702 |
| N | 2.3e+04 | 2.3e+04 | 2.3e+04 | 2.3e+04 |

*t* statistics in parentheses

^*^ *p* < 0.05, ^**^ *p* < 0.01, ^***^ *p* < 0.001

. sgmediation PAT , mv( Dist ) iv( DIF) cv( Size Age lev Top1 Mfee Growth ROE TobinQ Mshare Indep Dual SOE Board)

Model with dv regressed on iv (path c)

Source SS df MS Number of obs = 22,781

F(14, 22766) = 122.80

Model 3753753.94 14 268125.281 Prob > F = 0.0000

Residual 49706254.8 22,766 2183.35477 R-squared = 0.0702

Adj R-squared = 0.0696

Total 53460008.7 22,780 2346.79582 Root MSE = 46.726

PAT Coefficient Std. err. t P>t [95% conf. interval]

DIF .0312686 .0050584 6.18 0.000 .0213539 .0411833

Size 8.115412 .2781676 29.17 0.000 7.570185 8.66064

Age -13.67738 1.041053 -13.14 0.000 -15.71792 -11.63685

lev .2797599 1.424866 0.20 0.844 -2.513074 3.072594

Top1 12.20709 2.170809 5.62 0.000 7.95216 16.46203

Mfee .0036814 .0220919 0.17 0.868 -.0396202 .0469829

Growth .4388225 .3660334 1.20 0.231 -.2786279 1.156273

ROE 1.593928 1.414874 1.13 0.260 -1.179322 4.367179

TobinQ -1.745722 .4290897 -4.07 0.000 -2.586767 -.9046774

Mshare -22.94398 6.738755 -3.40 0.001 -36.1524 -9.735563

Indep 40.04403 6.519793 6.14 0.000 27.26479 52.82326

Dual 2.544813 .7705386 3.30 0.001 1.034505 4.055121

SOE 1.217482 .7176025 1.70 0.090 -.1890676 2.624032

Board -.4595285 1.900468 -0.24 0.809 -4.184575 3.265518

_cons -155.9543 7.594825 -20.53 0.000 -170.8407 -141.0679

Model with mediator regressed on iv (path a)

Source SS df MS Number of obs = 22,781

F(14, 22766) = 60.32

Model 130128.183 14 9294.87019 Prob > F = 0.0000

Residual 3508191.06 22,766 154.097824 R-squared = 0.0358

Adj R-squared = 0.0352

Total 3638319.24 22,780 159.715507 Root MSE = 12.414

Dist Coefficient Std. err. t P>t [95% conf. interval]

DIF -.0117422 .0013438 -8.74 0.000 -.0143762 -.0091082

Size -.1563337 .0738997 -2.12 0.034 -.3011822 -.0114853

Age -1.786105 .2765724 -6.46 0.000 -2.328206 -1.244005

lev 1.867759 .3785385 4.93 0.000 1.125798 2.60972

Top1 2.506026 .5767104 4.35 0.000 1.375635 3.636418

Mfee -.0036889 .0058691 -0.63 0.530 -.0151927 .0078148

Growth -.9791781 .0972427 -10.07 0.000 -1.16978 -.7885758

ROE .6981927 .3758841 1.86 0.063 -.0385659 1.434951

TobinQ -.1407032 .1139946 -1.23 0.217 -.3641403 .082734

Mshare -3.795314 1.790259 -2.12 0.034 -7.304343 -.2862857

Indep .9430657 1.732088 0.54 0.586 -2.451944 4.338076

Dual 1.025632 .204706 5.01 0.000 .6243944 1.42687

SOE -2.998148 .1906426 -15.73 0.000 -3.371821 -2.624476

Board 1.433951 .5048899 2.84 0.005 .444332 2.423569

_cons 21.58591 2.017687 10.70 0.000 17.63111 25.54072

Model with dv regressed on mediator and iv (paths b and c')

Source SS df MS Number of obs = 22,781

F(15, 22765) = 115.74

Model 3787997.16 15 252533.144 Prob > F = 0.0000

Residual 49672011.5 22,765 2181.94648 R-squared = 0.0709

Adj R-squared = 0.0702

Total 53460008.7 22,780 2346.79582 Root MSE = 46.711

PAT Coefficient Std. err. t P>t [95% conf. interval]

Dist -.0987974 .0249391 -3.96 0.000 -.1476797 -.0499151

DIF .0301085 .0050652 5.94 0.000 .0201804 .0400366

Size 8.099967 .2781052 29.13 0.000 7.554862 8.645072

Age -13.85384 1.04167 -13.30 0.000 -15.89559 -11.8121

lev .4642897 1.425168 0.33 0.745 -2.329136 3.257715

Top1 12.45468 2.171009 5.74 0.000 8.199358 16.71001

Mfee .0033169 .0220849 0.15 0.881 -.039971 .0466049

Growth .3420822 .3667293 0.93 0.351 -.3767322 1.060897

ROE 1.662908 1.414525 1.18 0.240 -1.109658 4.435474

TobinQ -1.759624 .4289656 -4.10 0.000 -2.600425 -.9188217

Mshare -23.31895 6.737246 -3.46 0.001 -36.52441 -10.11349

Indep 40.1372 6.517732 6.16 0.000 27.362 52.9124

Dual 2.646143 .7707146 3.43 0.001 1.135489 4.156796

SOE .9212728 .7212571 1.28 0.202 -.4924404 2.334986

Board -.3178578 1.900192 -0.17 0.867 -4.042363 3.406647

_cons -153.8216 7.611437 -20.21 0.000 -168.7406 -138.9027

Sobel-Goodman Mediation Tests

Coef Std Err Z P>Z

Sobel .0011601 .00032153 3.608 .00030851

Goodman-1 (Aroian) .0011601 .00032327 3.589 .00033245

Goodman-2 .0011601 .00031978 3.628 .00028584

Coef Std Err Z P>Z

a coefficient = -.011742 .001344 -8.73785 0

b coefficient = -.098797 .024939 -3.96155 .000074

Indirect effect = .00116 .000322 3.60805 .000309

Direct effect = .030109 .005065 5.94419 2.8e-09

Total effect = .031269 .005058 6.18157 6.3e-10

Proportion of total effect that is mediated: .03710116

Ratio of indirect to direct effect: .0385307

Ratio of total to direct effect: 1.0385307

.

. outreg2 using Dist1.rtf, replace tstat bdec(4) tdec(4) sym(***, **, *) ctitle(satisfaction) e( r2 F )

Dist1.rtf

dir : seeout

.

. bootstrap r(ind_eff) r(dir_eff),reps(1000) : sgmediation PAT , mv( Dist ) iv( DIF) cv( Size Age lev Top1 Mfee Growth ROE TobinQ

> Mshare Indep Dual SOE Board) quietly

(running sgmediation on estimation sample)

Bootstrap replications (1,000)

1 ---+--- 2 ---+--- 3 ---+--- 4 ---+--- 5

.................................................. 50

.................................................. 100

.................................................. 150

.................................................. 200

.................................................. 250

.................................................. 300

.................................................. 350

.................................................. 400

.................................................. 450

.................................................. 500

.................................................. 550

.................................................. 600

.................................................. 650

.................................................. 700

.................................................. 750

.................................................. 800

.................................................. 850

.................................................. 900

.................................................. 950

.................................................. 1,000

Bootstrap results Number of obs = 22,781

Replications = 1,000

Command: sgmediation PAT, mv( Dist ) iv( DIF) cv( Size Age lev Top1 Mfee Growth ROE TobinQ Mshare Indep Dual SOE Board)

quietly

_bs_1: r(ind_eff)

_bs_2: r(dir_eff)

Observed Bootstrap Normal-based

coefficient std. err. z P>z [95% conf. interval]

_bs_1 .0011601 .0003203 3.62 0.000 .0005324 .0017878

_bs_2 .0301085 .0042891 7.02 0.000 .0217021 .0385149

.

. estat bootstrap, percentile bc

Bootstrap results Number of obs = 22,781

Replications = 1000

Command: sgmediation PAT, mv( Dist ) iv( DIF) cv( Size Age lev Top1 Mfee Growth ROE TobinQ Mshare Indep Dual SOE Board)

quietly

_bs_1: r(ind_eff)

_bs_2: r(dir_eff)

Observed Bootstrap

coefficient Bias std. err. [95% conf. interval]

_bs_1 .0011601 -.0000233 .00032026 .0005406 .0017987 (P)

.0006327 .0019076 (BC)

_bs_2 .03010851 -.0001495 .00428908 .0218138 .038522 (P)

.0219127 .0388083 (BC)

Key: P: Percentile

BC: Bias-corrected

**Table 13. Impact of intellectual property protection and environmental governance.**

|  | (1) | (2) | (3) |
| --- | --- | --- | --- |
|  | PAT | INPAT | UPAT |
| DIF_ipp | 7.4315^***^ | 3.9876^**^ | 3.4439^***^ |
|  | (4.0553) | (3.2631) | (4.4496) |
|  |  |  |  |
| DIF | -7.3888^***^ | -3.9614^**^ | -3.4274^***^ |
|  | (-4.0411) | (-3.2489) | (-4.4385) |
|  |  |  |  |
| ipp | -1.2e+03^**^ | -5.2e+02 | -7.0e+02^***^ |
|  | (-2.8653) | (-1.7880) | (-4.0607) |
|  |  |  |  |
| Size | 8.0968^***^ | 5.1326^***^ | 2.9643^***^ |
|  | (12.5779) | (11.1453) | (13.4738) |
|  |  |  |  |
| Age | -13.6948^***^ | -7.1346^***^ | -6.5601^***^ |
|  | (-7.6485) | (-6.9977) | (-7.4951) |
|  |  |  |  |
| lev | 0.4639 | -1.9015 | 2.3654^***^ |
|  | (0.2622) | (-1.5431) | (3.5376) |
|  |  |  |  |
| Top1 | 12.2410^**^ | 2.6351 | 9.6060^***^ |
|  | (3.1365) | (1.1003) | (5.2309) |
|  |  |  |  |
| Mfee | 0.0040^***^ | 0.0024^**^ | 0.0016^***^ |
|  | (3.6461) | (3.2167) | (3.8271) |
|  |  |  |  |
| Growth | 0.4093 | 0.3268^*^ | 0.0826 |
|  | (1.8131) | (2.0549) | (0.9759) |
|  |  |  |  |
| ROE | 1.7697^*^ | 0.5265 | 1.2432^***^ |
|  | (2.3796) | (1.1022) | (3.8867) |
|  |  |  |  |
| TobinQ | -1.7097^***^ | -0.7011^***^ | -1.0087^***^ |
|  | (-4.6253) | (-3.3563) | (-5.4951) |
|  |  |  |  |
| Mshare | -22.6286^***^ | -12.8866^***^ | -9.7420^***^ |
|  | (-5.7789) | (-5.4359) | (-5.5247) |
|  |  |  |  |
| Indep | 39.0889^***^ | 18.5911^***^ | 20.4978^***^ |
|  | (4.0539) | (3.3345) | (4.2817) |
|  |  |  |  |
| Dual | 2.5394^**^ | 2.0232^***^ | 0.5162 |
|  | (3.1026) | (3.4933) | (1.7888) |
|  |  |  |  |
| SOE | 0.9420 | 0.8755^*^ | 0.0664 |
|  | (1.6315) | (2.1454) | (0.3063) |
|  |  |  |  |
| Board | -0.4420 | 1.4748 | -1.9168^*^ |
|  | (-0.1837) | (0.8741) | (-2.1316) |
|  |  |  |  |
| _cons | 1.1e+03^*^ | 411.9147 | 648.4433^***^ |
|  | (2.4948) | (1.4216) | (3.7707) |
| F | 21.5266 | 18.4670 | 24.7870 |
| r2_a | 0.0702 | 0.0526 | 0.0776 |
| N | 2.3e+04 | 2.3e+04 | 2.3e+04 |

*t* statistics in parentheses

^*^ *p* < 0.05, ^**^ *p* < 0.01, ^***^ *p* < 0.001

|  | (1) | (2) | (3) |
| --- | --- | --- | --- |
|  | PAT | INPAT | UPAT |
| DIF_FREQ | 15.4763^***^ | 8.1936^**^ | 7.2827^***^ |
|  | (3.8678) | (2.9655) | (4.6061) |
|  |  |  |  |
| DIF | -0.0163 | -0.0059 | -0.0104^**^ |
|  | (-1.5358) | (-0.7743) | (-2.6758) |
|  |  |  |  |
| FREQ | -1.8e+03^**^ | -8.3e+02^*^ | -9.8e+02^***^ |
|  | (-3.0195) | (-2.0343) | (-3.9916) |
|  |  |  |  |
| Size | 8.0531^***^ | 5.1097^***^ | 2.9435^***^ |
|  | (12.5861) | (11.1728) | (13.4507) |
|  |  |  |  |
| Age | -13.3956^***^ | -6.9448^***^ | -6.4509^***^ |
|  | (-7.6566) | (-6.9888) | (-7.5166) |
|  |  |  |  |
| lev | 0.4443 | -1.9479 | 2.3921^***^ |
|  | (0.2504) | (-1.5769) | (3.5583) |
|  |  |  |  |
| Top1 | 12.5895^**^ | 2.8560 | 9.7335^***^ |
|  | (3.2112) | (1.1952) | (5.2470) |
|  |  |  |  |
| Mfee | 0.0042^***^ | 0.0025^***^ | 0.0017^***^ |
|  | (3.8506) | (3.3617) | (4.0429) |
|  |  |  |  |
| Growth | 0.3510 | 0.3011 | 0.0499 |
|  | (1.5717) | (1.9140) | (0.5986) |
|  |  |  |  |
| ROE | 1.4136 | 0.2885 | 1.1251^***^ |
|  | (1.8820) | (0.5832) | (3.5995) |
|  |  |  |  |
| TobinQ | -1.6652^***^ | -0.6634^**^ | -1.0017^***^ |
|  | (-4.4654) | (-3.1137) | (-5.4630) |
|  |  |  |  |
| Mshare | -22.6360^***^ | -12.8010^***^ | -9.8350^***^ |
|  | (-5.8138) | (-5.4112) | (-5.6216) |
|  |  |  |  |
| Indep | 39.7797^***^ | 19.1055^***^ | 20.6742^***^ |
|  | (4.1162) | (3.4139) | (4.3124) |
|  |  |  |  |
| Dual | 2.5329^**^ | 2.0284^***^ | 0.5045 |
|  | (3.0887) | (3.4991) | (1.7417) |
|  |  |  |  |
| SOE | 1.0827 | 0.9968^*^ | 0.0860 |
|  | (1.8375) | (2.3894) | (0.3898) |
|  |  |  |  |
| Board | -0.5043 | 1.4334 | -1.9376^*^ |
|  | (-0.2097) | (0.8506) | (-2.1540) |
|  |  |  |  |
| _cons | -1.5e+02^***^ | -1.0e+02^***^ | -48.7420^***^ |
|  | (-11.5265) | (-10.1806) | (-11.9496) |
| F | 21.3677 | 18.3372 | 24.5045 |
| r2_a | 0.0719 | 0.0536 | 0.0797 |
| N | 2.3e+04 | 2.3e+04 | 2.3e+04 |

*t* statistics in parentheses

^*^ *p* < 0.05, ^**^ *p* < 0.01, ^***^ *p* < 0.001
